# Supplementary material for: Mass spectrometry of short peptides reveals common features of metazoan peptidergic neurons
Source: Nat Ecol Evol. 2022 Aug 8;6(10):1438–48. doi: 10.1038/s41559-022-01835-7 (PMC9525235; doi:10.1038/s41559-022-01835-7)
Supplement: Supplementary file 1 — Supplementary Methods 1–9, Figs. 1–8. [file 41559_2022_1835_MOESM1_ESM.pdf]

nature portfolio

https://doi.org/10.1038/s41559-022-01835-7

Supplementary information

Mass spectrometry of short peptides reveals common features of metazoan peptidergic neurons

In the format provided by the authors and unedited

Supplementary information

Mass spectrometry of short peptides reveals common features of metazoan peptidergic neurons

Eisuke Hayakawa*, Christine Guzman, Osamu Horiguchi, Chihiro Kawano, Akira Shiraishi, Kurato Mohri, Mei Fang Lin, Ryotaro Nakamura, Ryo Nakamura, Erina Kawai, Shinya Komoto, Kei Jokura, Kogiku Shiba, Shuji Shigenobu, Honoo Satake, Kazuo Inaba & Hiroshi Watanabe*

1. Supplementary methods

1.1 Sample preparation for mass spectrometry

1.2 Mass spectrometry analysis

1.3 Peptide identification

1.4 Cleavage site analysis

1.5 Homology analysis for peptide precursors

1.6 Antibody validation

1.7 siRNA-mediated knockdown of neuropeptides in *N.vectensis* embryos

1.8 Quantitative reverse transcription polymerase chain reaction (qPCR)

1.9 Reproducibility of microscopic data

2. Supplementary figures

2.1 Supplementary Figure 1

2.2 Supplementary Figure 2

2.3 Supplementary Figure 3

2.4 Supplementary Figure 4

2.5 Supplementary Figure 5

2.6 Supplementary Figure 6

2.7 Supplementary Figure 7

2.8 Supplementary Figure 8

1. Supplementary methods

1.1 Sample preparation for mass spectrometry

For Nematostella vectensis, six young adult polyps (about 10 mm in length) were mixed with 4 ml of acidified methanol solution (90% methanol, 9% ultrapure water, and 1% formic acid) together with zirconia beads (2.0 mm diameter) and immediately subjected to homogenization. For *Ephydatia*

fluviatilis, ten animals (about 10 mm diameters) were mixed with 4 ml of acidified methanol together with the same zirconia beads and immediately subjected to homogenization. For *Bolinopsis mikado*, immediately before extraction, three animals were cut into small pieces, then a large part of the mesoglea was removed with a surgical knife. The remaining tissue was mixed with 4 ml of acidified methanol together with the same zirconia beads and immediately subjected to homogenization. Homogenizations were performed with a bead homogenizer (Bead Smash 12, Waken B Tech) for three cycles at 3,000 rpm for 30 sec at 4 °C with an intermediate 30-sec pause. After homogenization, debris and large proteins were removed from the samples by centrifugation (4 °C at 13,000 g for 20 min). The supernatant was first vacuum dried with GeneVac EZ-2 Elite (SP Scientific) and then resuspended in 500 µl of 1% methanol with 0.5% formic acid, then subjected to the solid phase extraction. Oasis HLB 1 cc cartridge (30 mg, Waters) was used for solid-phase extraction together with vacuum manifold (Extraction Manifold, Waters). The cartridges were first activated with 1 ml of solvent B (70% acetonitrile with 0.1% formic acid), followed by equilibration with 1 ml of solvent A (1% acetonitrile with 0.1% formic acid). Resuspended samples were loaded onto the cartridges and washed with 1 ml of solvent A. The bound peptides were eluted with 1 ml of solvent B. The eluents were dried with GeneVac EZ-2 Elite and stored at -80 °C until LC-MS/MS analysis. This sample preparation procedure was performed independently four times for each animal and the resultants were subjected to the following analysis separately. One of the four extracted replicates was subjected to fractionation to reduce sample complexity before LC-MS/MS analysis. Fractionation was performed according to high-pH RP protocol[1] with slight modification. Dionex UltiMate 3000 HPLC system (Thermo Fischer Scientific) was used for fractionation together with XBridge Peptide BEH C18 Column (2.1 x 100 mm, 3.5 µm particles, 300 Å pores). Mobile phase A is 5 mM ammonium hydroxide (pH 10) and B is 90% acetonitrile, 5 mM ammonium hydroxide (pH 10). The flow rate was set to 0.2 ml min^{-1} and the column temperature was maintained at 40 °C during the runs. The samples were fractionated with a 53 min gradient as follows: 1% B in 3 min, 1-25% B in 27 min, 25-40% B in 13 min, 40-80% B in 5 min, 80% B in 5 min. Fractions were made ~~for~~ every 50 sec, resulting in a total of 72 fractions. The fractions were then pooled and concatenated with other fractions to create 12 combined fractions as described by Wang *et al*.[2]. The fractions were dried with GeneVac EZ-2 Elite and stored at -80 °C until LC-MS/MS analysis.

1.2 Mass spectrometry analysis

Three non-fractionated samples and pooled fractionated samples from each animal were analyzed by means of the LC-MS/MS analysis as follows. Dried samples were resuspended in 35 µL of resuspension solution (5% methanol with 0.1% formic acid) with iRT peptides (Biognosys). Fusion Lumos (Thermo Fisher Scientific) coupled with nano-flow liquid chromatography (nanoACQUITY, Waters) was used for LC-MS/MS analysis. The samples were loaded by auto-sampler into a trap

column (nanoACQUITY UPLC 2G-V/M Trap 5μm Symmetry C18, 180 μm x 20 mm, Waters) and subsequently an analytical column (nanoACQUITY UPLC HSS T3 1.8 um, 75 μm x 150 mm). Mobile phase A is 0.1% formic acid in water and B is 0.1% formic acid in 80% acetonitrile. The flow rate was set to 500 nl min^{-1} and the column temperature was maintained at 40 °C during the runs. The samples were separated with a 65 min gradient as follows: 1-7% B in 1 min, 7-13% B in 1 min, 13-50% B in 38 min, 50-99% B in 1 min, 99% B in 9 min, 99-1% B in 1 min and 1% B in 14 min. The eluent was ionized by electrospray ionization with the following setting (RF lens: 30%; spray voltage: 2020 V at 0 min, 2020 V at 30 min, 2400 V at 40 min, 2800 V at 50 min, 2020 V at 55 min; sheath gas: 16; auxiliary gas: 2; ion transfer tube temperature: 305 °C). Fragment ion spectra of peptides were acquired in data-dependent acquisition approach using CHarge Ordered Parallel Ion aNalysis[3] with slight modification. Precursor ions were analyzed in the Orbitrap (AGC target 4 x 10^5, 50 ms maximum injection time, 120,000 mass resolution). For every survey scan, the top 10 most intense peptide ions were selected for fragmentation at a normalized collision energy of 25% (HCD) or 28% (CID) based on their intensities and their fragment ion spectra acquired in the ion trap detector. Our aim was to perform systematic peptide identifications for three target animals with similar analytical conditions. To this end, prior to the main LC-MS/MS analysis for peptide identifications, we first performed an analysis to acquire signal abundances of each sample. The relative signal abundance of samples is estimated based on the total ion current of each LC-MS run. The loading volume is adjusted so each injection has roughly similar amounts, as well as ion current in the LC-M/MS analysis.

1.3 Peptide identification

Peptide-to-spectrum matching was performed using PEAKS X software (PEAKS Studio version 10.0, Bioinformatics Solutions) and Mascot (version 2.7, Matrix Science). For PEAKS X, the raw data recorded with Xcalibur (version 4.5, Thermo Fisher Scientific) were directly used. The raw data of the acquired MS/MS spectra were processed with PEAKS X software (Bioinformatics Solutions). PEAKS DB algorithms were utilized to perform peptide-to-spectrum matchings. The search parameters are as follows: 3 ppm for precursor tolerance, 0.3 Da for fragment tolerance, and no fixed modifications. Variable modifications were oxidation of Methionine (15.99491 Da), pyroglutamation from Glutamic acid (-18.01057 Da) or Glutamine (-17.02655Da), and amidation at peptide C-termini (-0.98402 Da) for PEAKSDB or Glycine-loss amide (-58.005479) for Mascot. For Mascot, fragment ion spectra were extracted in mgf file format from the raw format using MS convert in ProteoWizard package[4] 3.0.20139. The same search parameters for mass tolerance and variable PTM were used for Mascot as well. Searches were performed against amino acid sequences translated in six frames from the transcriptome data of the respective animal species. Transcriptome sequence data for *B. Mikado* (Yokohama, Kanagawa) was prepared as described in the main

manuscript and data sets of other species were acquired from public data set[5-7]. It is known that extracted peptide fractions may contain naturally occurring protein fragments as well as protein degradation products caused during the sample processing, which are not relevant to neuropeptides. In order to remove such peptides from the peptide-to-spectrum matching result, the following filtering process was performed. HMMER[8] (version 3.2.1) was used to scan precursor proteins of detected peptides for Pfam[9] motifs version 33.1. All peptides whose precursor protein included any Pfam motifs except for neuropeptide-related motifs (PF05874,PF11109,PF14993,PF15085,PF15180,PF08257,PF15161,PF05953,PF01160,PF04736,PF08111,PF15171,PF02323,PF03858,PF05824,PF08035,PF08258,PF08259,PF08260,PF17308,PF00123,PF01581,PF07421,PF02202,PF11105,PF02044,PF08187,PF00473,PF00918,PF01147,PF03002) with an e-value cutoff of 1e-10 were removed and not processed in the following analysis.

In this study, we focus on naturally occurring peptides with C-terminal amidation as neuropeptide candidates. To this end, only peptides detected as C-terminally amidated forms were selected and further analyzed. Validation of peptide identification was performed as follows. We consider proteins that produce C-terminally amidated peptide predicted with peptide-to-spectrum matching as tentative peptide precursors. Since we had a large number of peptide candidates, we select one of peptides derived from each precursor protein~~s~~ as representative and their AA sequences were chemically synthesized. For *N. vectensis*, peptides that show the same structure or derived from previously identified neuropeptide precursors[10] were considered as valid identification, and peptides from novel precursors were selected for peptide synthesis. Peptide synthesis (>80% purity) was performed using ResPep SL (intavis). The synthesized peptides were subjected to LC-MS/MS analysis using the same settings as described above. Fragment ion spectra acquired from the synthetic peptides were manually inspected and compared to the spectra of endogenously detected peptides. The criterions are (1) the presence of the same product ions used for peptide-spectrum matching and (2) relative intensities of the matched ions. If a representative peptide was validated with synthetized peptides, other peptide-to-spectrum matching results derived from the same precursor protein were also considered valid. The raw LC-MS/MS data sets, the output of peptide-to-spectrum matching, six frame-translated AA sequence data sets used, the result of Pfam motif scan, the list of synthetic peptides, and the result of validation with synthetic peptides have been deposited to the ProteomeXchange Consortium with the data set identifier PXD030145 (https://repository.jpostdb.org/preview/194169514461ab01a6d09da, Access key : 1700).

1.4 Cleavage site analysis

Lists of endogenous peptides of, *Drosophila melanogaster*, *Lineus longissimus,* and *Homo sapiens* were acquired from the studies of mass spectrometry-based peptide identifications[11-13]. When length variants were found in the list of peptides, only the longest forms were used to annotate cleavage

sites to avoid endogenous or artificial degradation products. AA composition of N and C-terminal cleavage sites (2 AA, excluding Glycine for amide donor) of peptides were counted for each data set. For WebLogo representation, N and C-terminal flanking regions of 6 AA were collected from the data sets. In case the length of N or C-terminal flanking regions was less than 6 AA, such regions were excluded.

1.5 Homology analysis for Neuropeptide precursors

Sequence data of Ctenophora species (*Beroe abyssicola*, *Beroe sp*., *Bolinopsis ashleyi*, *Bolinopsis infundibulum*, *Coeloplana astericola*, *Dryodora glandiformis*, *Euplokamis dunlapae*, *Mertensiidae sp.*, *Mnemiopsis leidyi*, *Pukia falcata*, *Vallicula multiformis*) were acquired from Neurobase (https://neurobase.rc.ufl.edu/). Homologs of *B. mikado* neuropeptide precursors were searched against the sequences data by performing a local tblastn search (version 2.11.0+)[14]. Homologs of *N. vectensis* neuropeptide homologs were acquired by searching against NCBI transcriptome Shotgun Assembly of Cnidarian species using an online tblastn interface. Blast search results were manually validated based on the criteria (1) the presence of mature neuropeptide structurally related to the identified peptides of *N, vectensis,* or *B. mikado* and (2) the presence of Glycine at the C-terminal flanking region as amide donor. AA sequences of other neuropeptide precursors were acquired from Jekeley[15] and Thiel *et al*.[16]. The sequences of neuropeptide precursors were assembled (Supplementary data 4) and subjected to an all-against-all Basic Local Alignment Search Tool (BLAST) search (blastp, version 2.11.0+)[14]. Clustering and visualization were performed on Cytoscape software (version 3.5.1)[17] with an e-value cutoff of 1e-5.

1.6 Antibody validation

Validation of the primary antibodies against NPWa and VWYa was performed by negative control immunostaining for *B. mikado* and *V. multifomis*, respectively. The basic procedure of immunostaining was the same as the method section of the main manuscript., but negative control samples were incubated in blocking solution without the primary antibodies. Images of the negative control samples were recorded with the same laser intensity and exposure time. The recording was performed by the Olympus SD-OSR confocal microscopy (Supplementary Figure 6, 7).

1.7 siRNA-mediated knockdown of neuropeptides in *N. vectensis* embryos

In order to check the specificity of each antibody, we performed small interference RNA (siRNA)-mediated gene knockdown of neuropeptide precursor mRNAs, followed by immunostaining of neuropeptides. For this purpose, we examined at least four distinct siRNA sequences for each peptide gene and found siRNAs strongly (> 90%) disrupting the RFamide, PRGamide, and QWamide mRNAs (Supplementary Figure 8). We were not able to find the effective siRNA sequence

for HIRamide and therefore excluded this peptide from analysis. For knocking down neuropeptides of *N.vectensis*, siRNAs were electroporated to the fertilized eggs as described[19] with following modifications: Eggs were suspended in brackish water (1/3 artificial sea water) with 6% Ficoll. The eggs were electroporated with siRNAs for control (siCtr: 5’-GCAACACGCAGAGUCGUAAdTdT-3’), *NvRFamide* (si*RFa*: 5’-GCUUGGAAUCCUAAUUCAAdTdT-3’), *NvPRGamide* (si*PRGa*: 5’-GAUGAAGAAUCUUUACUUGdTdT-3’), *NvQWamide* (si*QWa*: 5’-GAAAUUCCGCCACAAGGUUdTdT-3’) at 100 ng/µl concentration. Planula larvae were collected for RNA extraction and immunostaining at 4 dpf. Knockdown efficiencies were assessed with qPCR analyses for each experiment.

1.8 Quantitative reverse transcription polymerase chain reaction (qPCR)

RT-qPCR was performed on the StepOne Plus™ Real-Time PCR System (Thermo Fisher Scientific) using PowerUp SYBR® Green Master Mix (Thermo Fisher Scientific). The expression levels of analyzed genes were normalized to *Ef1a* and *Gapdh* using ΔΔCt method. The primers sequences are listed in Supplementary Table 9.

1.9 Reproducibility of microscopic data

Figure2

a: n=16, b: n=7, c: n=21, d: n=7, e: n=3, f: n=13, g: n=22, h: n=7, i: n=15, j: n=15, k: n=8, l: n=19, m: n=6, n: n=13, o: n=7, p: n=3, q: n=18, r: n=3, s: n=11, t: n=3, u: n=15, v: n=7, w: n=5

Extended Figure 3

a whole body: RFa: n=8, RPGa: n=10, QWa: n=7, HIRa: n=9, sensory: RFa: n=8, RPGa: n=7, QWa: n=6, HIRa: n=9, multipolar: RFa: n=8, RPGa: n=10, QWa: n=7, HIRa: n=9

b whole body: RFa: n=5, RPGa: n=8, QWa: n=9, HIRa: n=8, neuroendocrine: RFa: n=5, RPGa: n=8, QWa: n=9, HIRa: n=8

Extended Figure 4

b, c: n=19, d: n=19, e: n=3, f, g: n=6, h: n=4, I, j: n=4, k: n=4, l: n=2, m: n=4, n, o: n=5, p, q: n=4

Supplementary Figure 6

a: n=8, b: n=2

Supplementary Figure 7

a: n=4, b: n=4, c: n=4, d: n=4

References

1 Batth, T. S., Francavilla, C. & Olsen, J. V. Off-line high-pH reversed-phase fractionation for in-depth phosphoproteomics. *J Proteome Res* **13**, 6176-6186, doi:10.1021/pr500893m (2014).

2 Wang, Y. *et al.* Reversed-phase chromatography with multiple fraction concatenation strategy for proteome profiling of human MCF10A cells. *Proteomics* **11**, 2019-2026, doi:10.1002/pmic.201000722 (2011).

3 Davis, S. *et al.* Expanding Proteome Coverage with CHarge Ordered Parallel Ion aNalysis (CHOPIN) Combined with Broad Specificity Proteolysis. *J Proteome Res* **16**, 1288-1299, doi:10.1021/acs.jproteome.6b00915 (2017).

4 Kessner, D., Chambers, M., Burke, R., Agus, D. & Mallick, P. ProteoWizard: open source software for rapid proteomics tools development. *Bioinformatics* **24**, 2534-2536, doi:10.1093/bioinformatics/btn323 (2008).

5 Alie, A. *et al.* The ancestral gene repertoire of animal stem cells. *Proc Natl Acad Sci U S A* **112**, E7093-7100, doi:10.1073/pnas.1514789112 (2015).

6 Tulin, S., Aguiar, D., Istrail, S. & Smith, J. A quantitative reference transcriptome for Nematostella vectensis early embryonic development: a pipeline for de novo assembly in emerging model systems. *Evodevo* **4**, 16, doi:10.1186/2041-9139-4-16 (2013).

7 Sullivan, J. C. *et al.* StellaBase: the Nematostella vectensis Genomics Database. *Nucleic Acids Res* **34**, D495-499, doi:10.1093/nar/gkj020 (2006).

8 Finn, R. D., Clements, J. & Eddy, S. R. HMMER web server: interactive sequence similarity searching. *Nucleic Acids Res* **39**, W29-37, doi:10.1093/nar/gkr367 (2011).

9 El-Gebali, S. *et al.* The Pfam protein families database in 2019. *Nucleic Acids Res* **47**, D427-D432, doi:10.1093/nar/gky995 (2019).

10 Hayakawa, E. *et al.* A combined strategy of neuropeptide prediction and tandem mass spectrometry identifies evolutionarily conserved ancient neuropeptides in the sea anemone Nematostella vectensis. *PLoS One* **14**, e0215185, doi:10.1371/journal.pone.0215185 (2019).

11 Thiel, D. *et al.* Nemertean, Brachiopod, and Phoronid Neuropeptidomics Reveals Ancestral Spiralian Signaling Systems. *Molecular Biology and Evolution* **38**, 4847-4866, doi:10.1093/molbev/msab211 (2021).

12 Wegener, C., Reinl, T., Jansch, L. & Predel, R. Direct mass spectrometric peptide profiling and fragmentation of larval peptide hormone release sites in Drosophila melanogaster reveals tagma-specific peptide expression and differential processing. *J Neurochem* **96**, 1362-1374, doi:10.1111/j.1471-4159.2005.03634.x (2006).

13 Hook, V. & Bandeira, N. Neuropeptidomics Mass Spectrometry Reveals Signaling Networks Generated by Distinct Protease Pathways in Human Systems. *J Am Soc Mass Spectrom* **26**, 1970-1980, doi:10.1007/s13361-015-1251-6 (2015).

14 Altschul, S. F., Gish, W., Miller, W., Myers, E. W. & Lipman, D. J. Basic local alignment search tool. *J Mol Biol* **215**, 403-410, doi:10.1016/S0022-2836(05)80360-2 (1990).

15 Jekely, G. Global view of the evolution and diversity of metazoan neuropeptide signaling. *Proc Natl Acad Sci U S A* **110**, 8702-8707, doi:10.1073/pnas.1221833110 (2013).

16 Thiel, D., Franz-Wachtel, M., Aguilera, F., Hejnol, A. & Wray, G. Xenacoelomorph Neuropeptidomes Reveal a Major Expansion of Neuropeptide Systems during Early Bilaterian Evolution. *Molecular Biology and Evolution* **35**, 2528-2543, doi:10.1093/molbev/msy160 (2018).

17 Lopes, C. T. *et al.* Cytoscape Web: an interactive web-based network browser. *Bioinformatics* **26**, 2347-2348, doi:10.1093/bioinformatics/btq430 (2010).

18 Pang, K. & Martindale, M. Q. Developmental expression of homeobox genes in the ctenophore Mnemiopsis leidyi. *Dev Genes Evol* **218**, 307-319, doi:10.1007/s00427-008-0222-3 (2008).

19 Masuda-Ozawa, T. *et al.* siRNA-mediated gene knockdown via electroporation in hydrozoan jellyfish embryos. *bioRxiv*, doi:10.1101/2022.03.24.485716 (2022).

20 Sebe-Pedros, A. *et al.* Early metazoan cell type diversity and the evolution of multicellular gene regulation. *Nat Ecol Evol* **2**, 1176-1188, doi:10.1038/s41559-018-0575-6 (2018).

2 Supplementary Figures

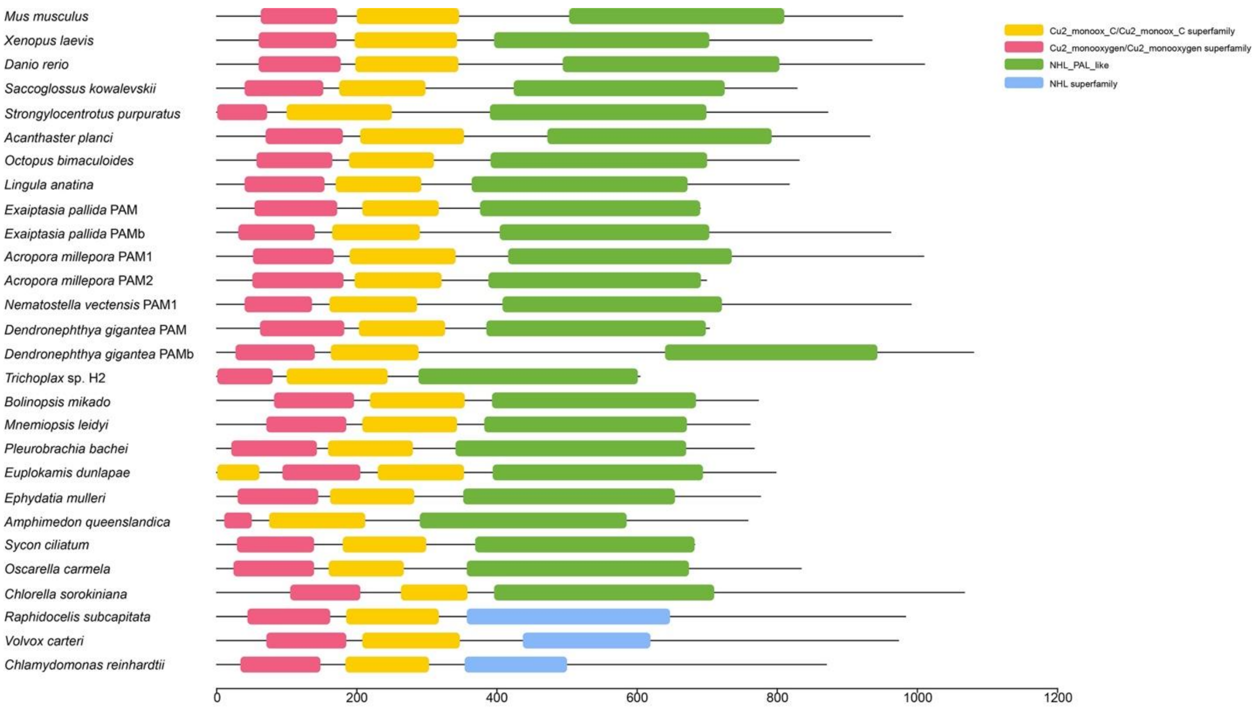

Supplementary Figure 1: Structure of the PAM genes with predicted funcitonal domains. The schematics showed the identified PAM genes from representative species in Metazoa and unicelluar organisms by NCBI conserved domain search interface and HMMER search against Pfam database v.35.0. The scale at the bottom indicates the length (number of residues) of sequences.

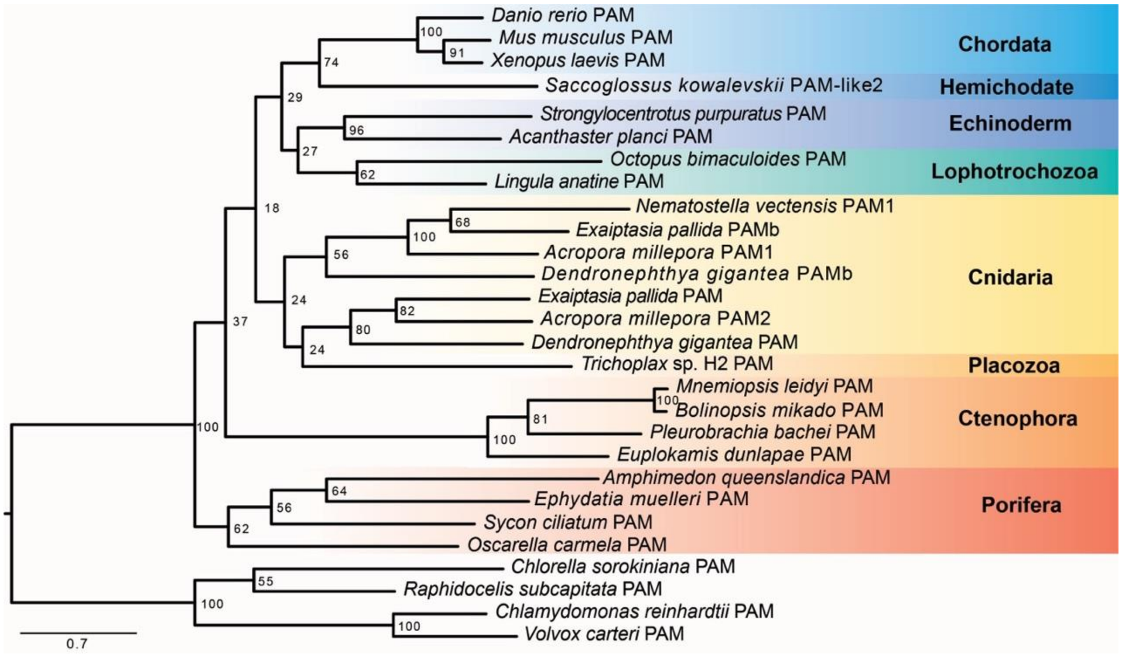

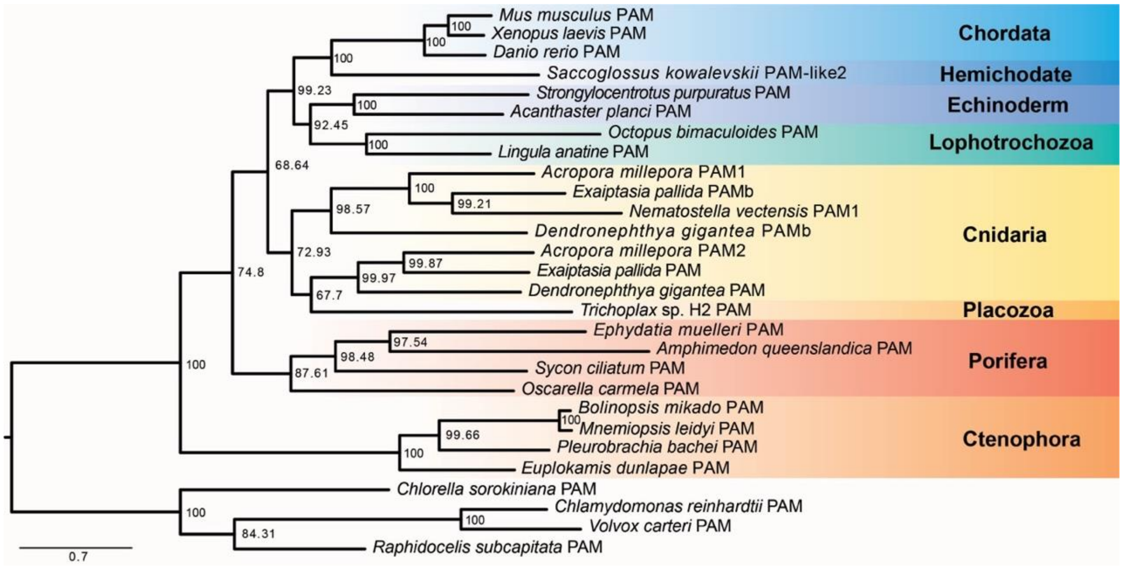

Supplementary Figure 2: Phylogenetic trees of PAM genes. Top: Maximum Likelihood tree, Bottom; Bayesian tree. The topologies were reconstructed under the WAG+I+G+F amino acid substitution model. Branching values shown represent the percentage of bootstrap supports under Maximum Likelihood analysis calculated in PhyML (top) and posterior probabilities under Bayesian inference implemented in MrBayes (bottom), respectively.

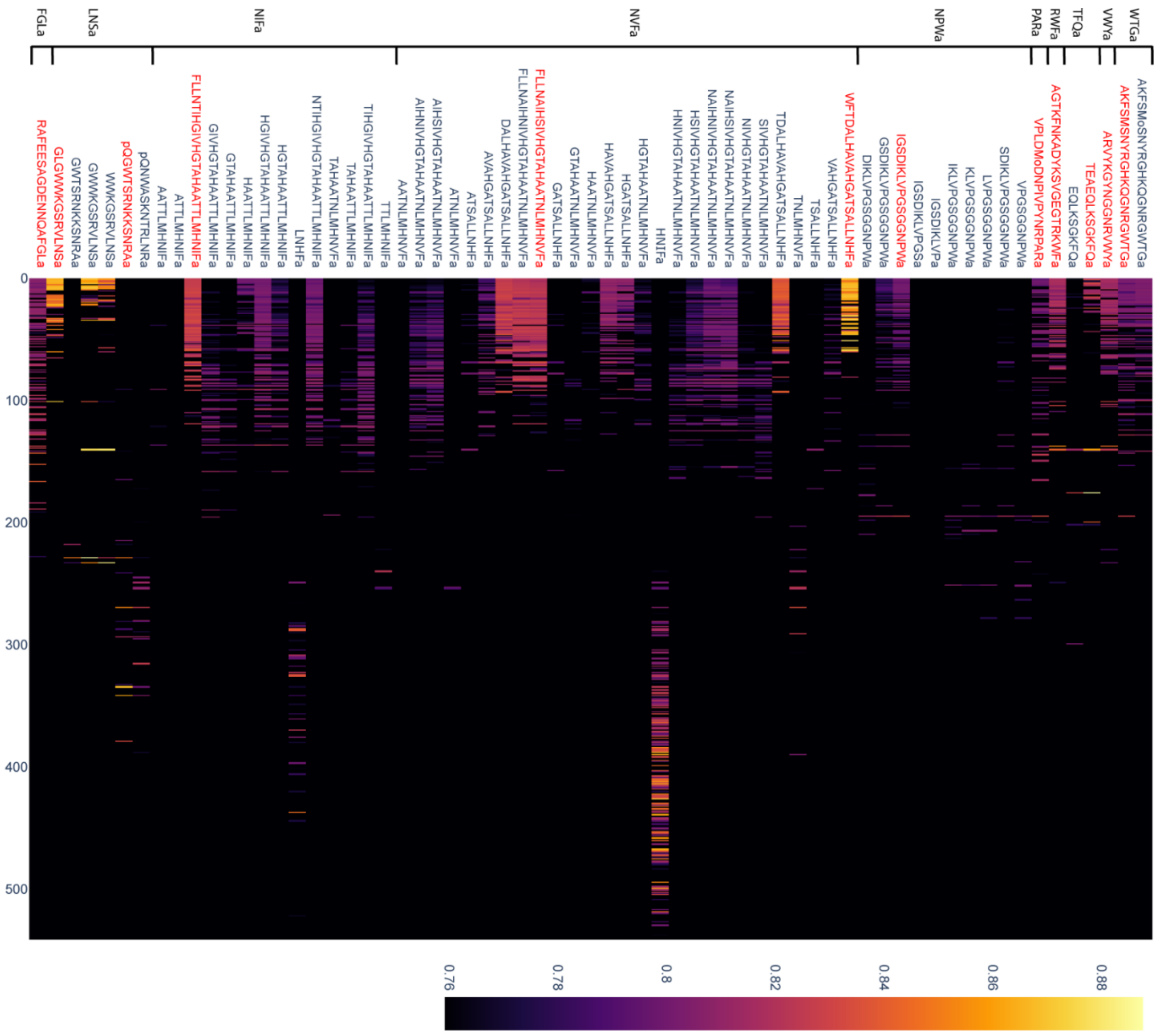

Supplementary Figure 3: Heatmap representation of peptide-GCPR pair prediction for all *M. leidyi* peptide homologs and GPCRs. Representative peptides for each gene were highlighted in red. Y-axis shows the indexes of GPCRs in the original result table (Supplementary Table 3). The order of the Y-axis was sorted by the summation of the scores.

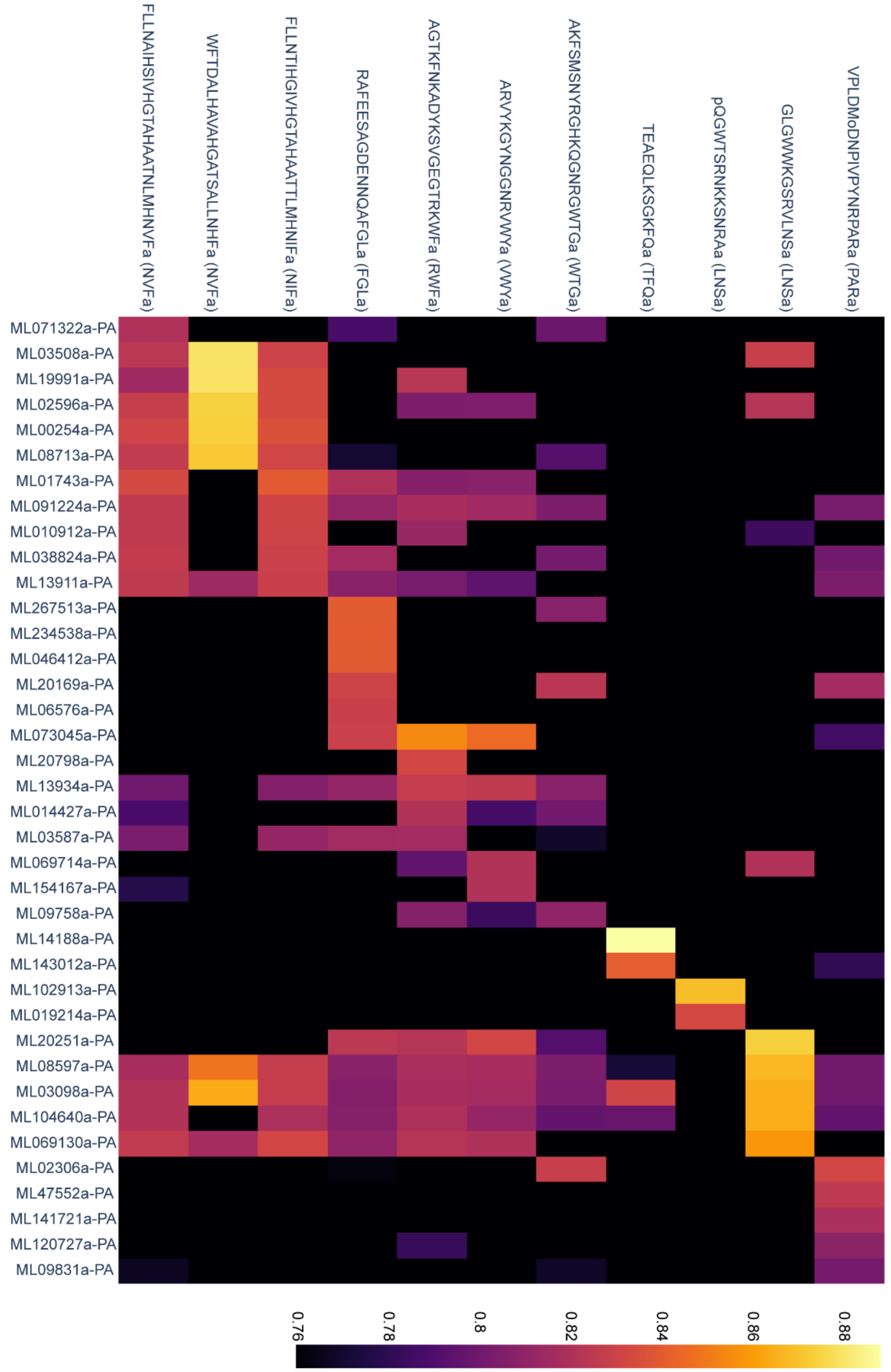

Supplementary Figure 4: Heatmap representation of peptide-GCPR pair prediction results shown in Figure 4f.

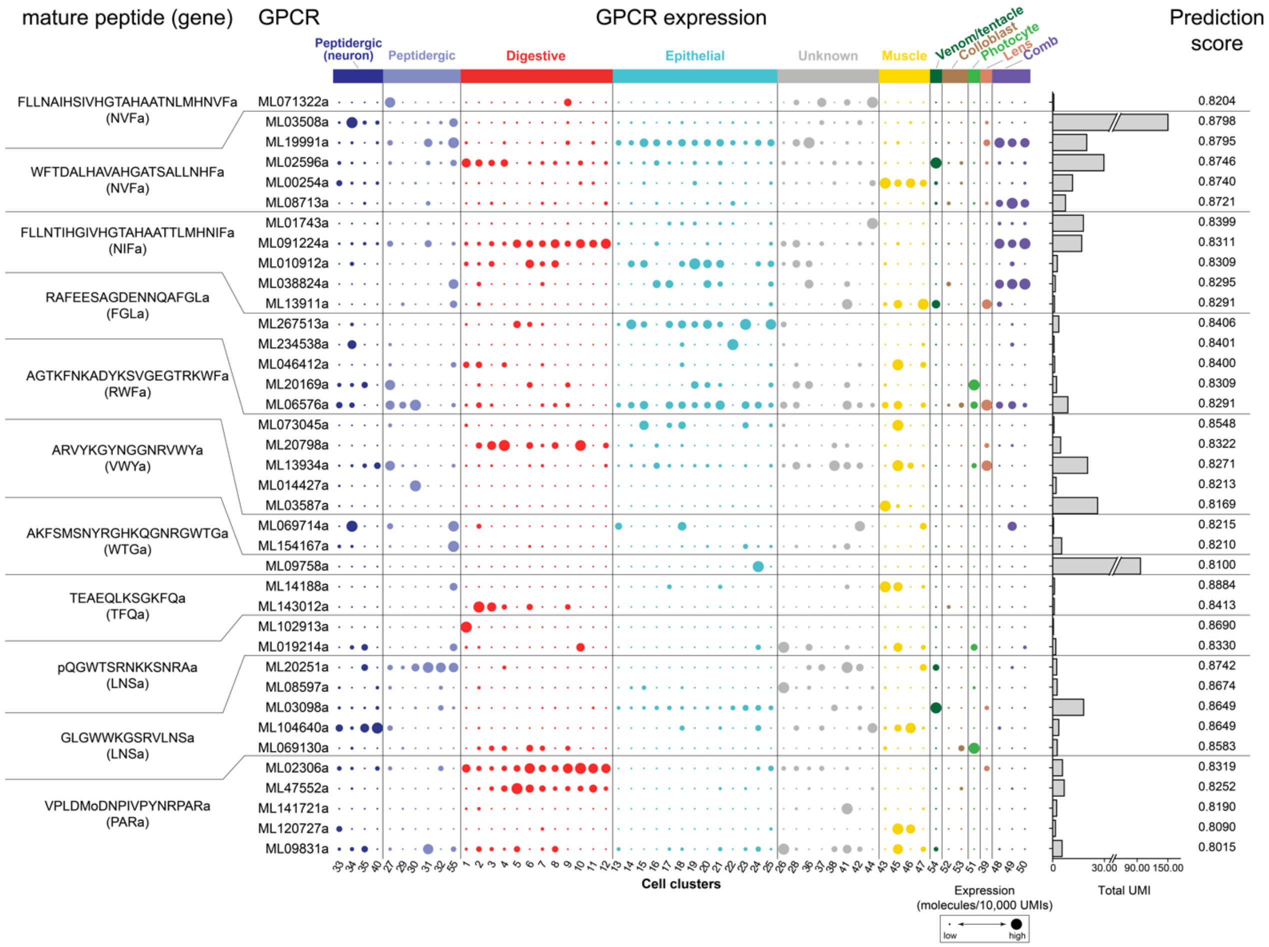

Supplementary Figure 5: Cell-cluster distribution of putative peptide GPCR receptor expression. The representative peptide sequences from each peptide gene (in parentheses) in *M. leidyi* are shown on the left. The dot plot shows the normalized expression values (molecules/10,000 UMIs) of each GPCR gene across the different cell clusters of adult *M. leidyi* scRNA-seq data[20] . The expressions are scaled by gene with dot size scales from smallest to largest, corresponding to lowest and highest expression, respectively. The bar plot shows the actual UMI values. The communication scores of each peptide-GPCR pair from the prediction model are indicated.

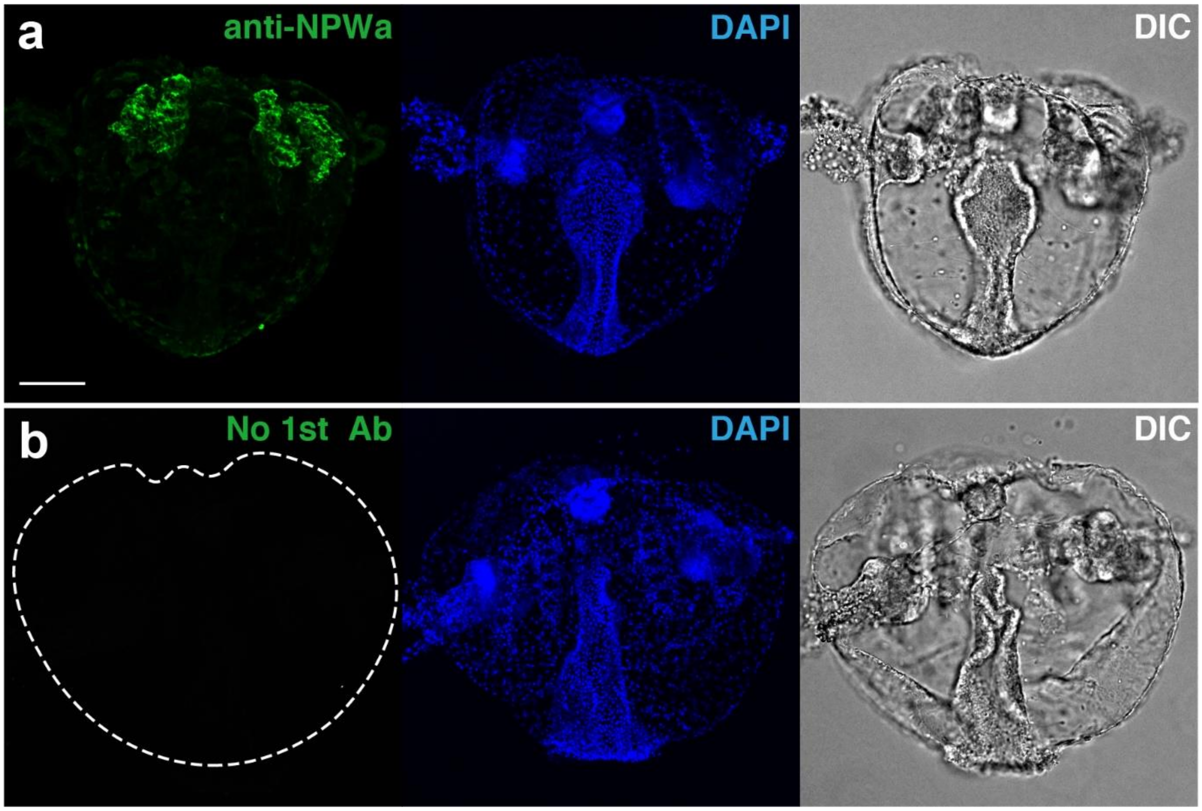

Supplementary Figure 6: Neuropeptide (NPWa) staining of *B. mikado* with or without the primary antibody. The images of immunostaining performed with (**a**) or without (**b**) anti-NPWa primary antibody. Neuropeptide (green), DAPI (blue), and DIC (bright field). The scale bar, 50 µm.

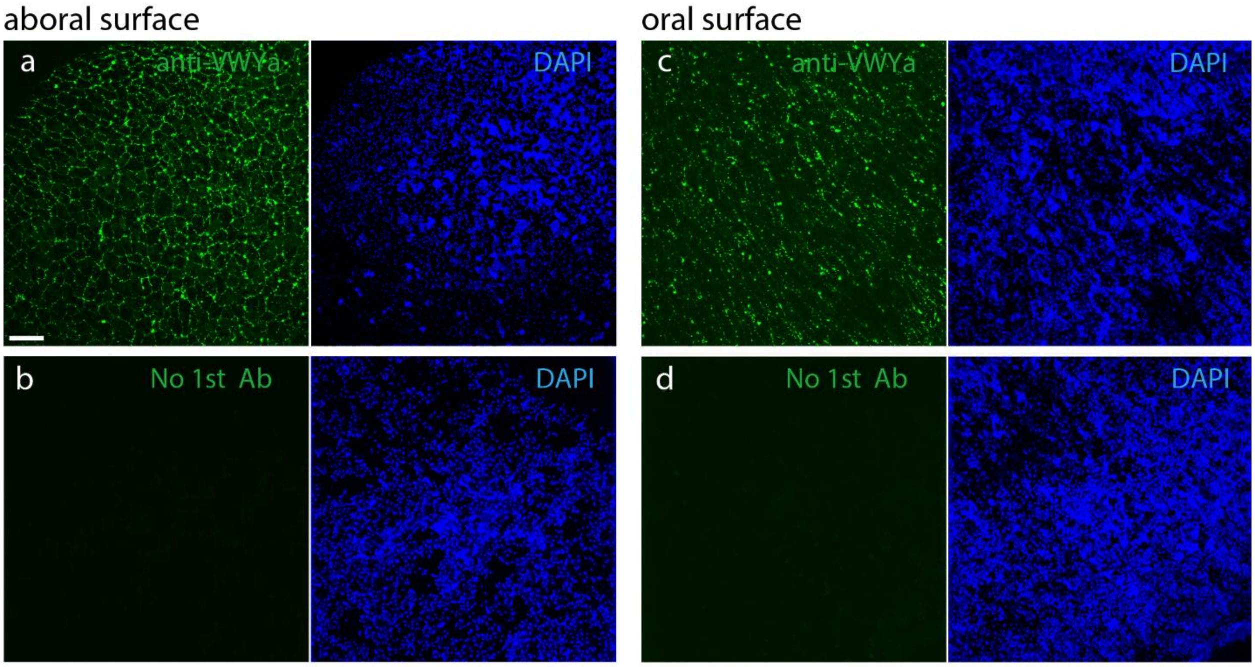

Supplementary Figure 7: Neuropeptide (VWYa) staining of *V. multiformis* with or without the primary antibody. Immunofluorescent staining was performed with (**a**, **c**) or without (**b**, **d**) anti-VWYa primary antibody. The images of **a**, **b** and **c**, **d** show aboral and oral surfaces, respectively. The images of Alexa488 (green) and DAPI (blue) fluorescence of the same view were indicated in each panel. Scale bar, 50 μm.

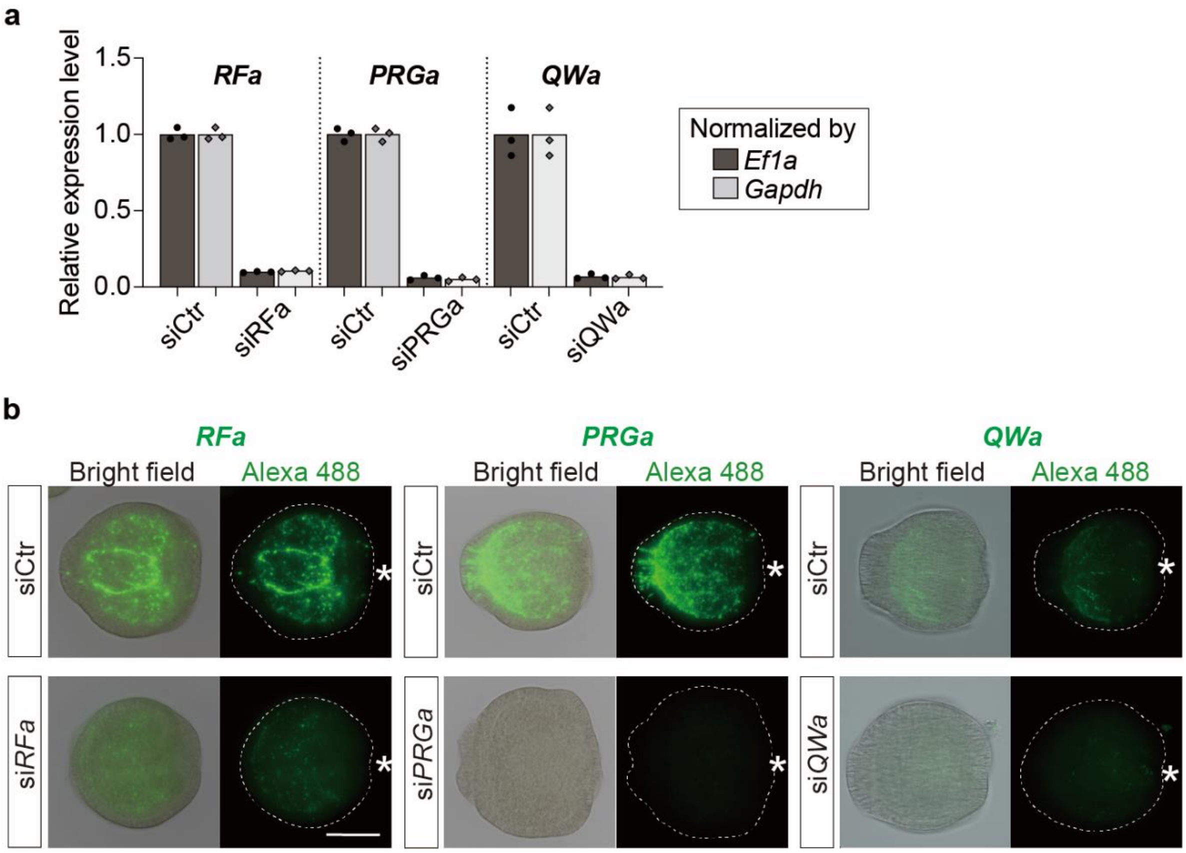

Supplementary Figure 8: siRNA-mediated neuropeptide gene knockdown demonstrates the antibody specificity of *N. vectensis* neuropeptides. **a**, qPCR data shows the relative expression level of neuropeptide mRNAs in 4dpf larvae transfected with siRNA for *RFamide*, *PRGamide*, or *QWamide* precursor mRNAs. Control siRNA (siCtr)-transfected samples were used as the control. The expression ratio normalized by *Ef1a* and *Gapdh* are indicated as dark and light grey bars, respectively. The data shown are from a single experiment and representative of at least two experiments with similar results.

b, Immunostaining of RFamide, PRGamide and QWamide neuropeptides in control siRNA-transfected (upper panels) or neuropeptide-specific siRNA-transfected (lower panels) larvae. Asterisk indicates the oral side. The scale bar, 100µm.
