# Supplementary material for: Mass spectrometry of short peptides reveals common features of metazoan peptidergic neurons
Source: Nat Ecol Evol. 2022 Aug 8;6(10):1438–48. doi: 10.1038/s41559-022-01835-7 (PMC9525235; doi:10.1038/s41559-022-01835-7)
Supplement: Supplementary file 2 — Reporting Summary [file 41559_2022_1835_MOESM2_ESM.pdf]

nature portfolio

Corresponding author(s): Hiroshi Watanabe

Last updated by author(s): Jun 8, 2022

Reporting Summary

Nature Portfolio wishes to improve the reproducibility of the work that we publish. This form provides structure for consistency and transparency in reporting. For further information on Nature Portfolio policies, see our Editorial Policies and the Editorial Policy Checklist.

Statistics

For all statistical analyses, confirm that the following items are present in the figure legend, table legend, main text, or Methods section.

n/a	Confirmed	
☐	☒	The exact sample size (n) for each experimental group/condition, given as a discrete number and unit of measurement
☐	☒	A statement on whether measurements were taken from distinct samples or whether the same sample was measured repeatedly
☐	☒	The statistical test(s) used AND whether they are one- or two-sided<br>*Only common tests should be described solely by name; describe more complex techniques in the Methods section.*
☒	☐	A description of all covariates tested
☐	☒	A description of any assumptions or corrections, such as tests of normality and adjustment for multiple comparisons
☐	☒	A full description of the statistical parameters including central tendency (e.g. means) or other basic estimates (e.g. regression coefficient) AND variation (e.g. standard deviation) or associated estimates of uncertainty (e.g. confidence intervals)
☐	☒	For null hypothesis testing, the test statistic (e.g. F, t, r) with confidence intervals, effect sizes, degrees of freedom and P value noted<br>*Give P values as exact values whenever suitable.*
☒	☐	For Bayesian analysis, information on the choice of priors and Markov chain Monte Carlo settings
☒	☐	For hierarchical and complex designs, identification of the appropriate level for tests and full reporting of outcomes
☒	☐	Estimates of effect sizes (e.g. Cohen's d, Pearson's r), indicating how they were calculated

Our web collection on statistics for biologists contains articles on many of the points above.

Software and code

Policy information about availability of computer code

Data collection	Fusion Lumos (Thermo Fisher Scientific) with Xcalibur (version 4.5, Thermo Fisher Scientific) was used for mass spectrometry.<br>RNA-seq was performed by using HiSeq 2500 (Illumina).<br>Fluorescent microscope images of N. vectensis was acquired using ECLIPSE Ni (Nikon) equipped with Ds-Ri2 (Nikon). Immunostaining images of B. Mikado were acquired using confocal microscopy (Olympus Fluoview FV10i). Immunostaining images of V. multiformis were recorded with a confocal microscopy system SD-OSR (Olympus) handled by the software Metamorph (Molecular devices, ver. 7.10.1.161).
Data analysis	ProteoWizard (package 3.0.20139) was used to convert mass spectrometry data. Peptide-to-spectrum matching was performed using PEAKS X software (PEAKS Studio version 10.0, Bioinformatics Solutions) and Mascot (version 2.7, Matrix Science).<br>Protein motif scan was performed using HMMER (version 3.2.1). Protein homology search was performed using BLAST blastp, version 2.11.0 +). Network visualization of neuropeptide precursor proteins were done using Cytoscape (version 3.5.1),<br>The transcriptome data was processed using libngs (https://github.com/sylvainforet/libngs), Trinity (v2.3.2), CD-HIT (v4.6.5) and TransDecoder (v3.0.1).<br>Sequence alignment was performed using MEGA (version 7.0.26). Codes used for gene expression profiling analysis: https://github.com/oist/scrna-counts.<br>Fluorescent microscope images of N. vectensis was processed with NIS-Elements AR (Version 5.02.03, Nikon). Image data were edited using ImageJ (ver. 2.5.30/1.53f).

For manuscripts utilizing custom algorithms or software that are central to the research but not yet described in published literature, software must be made available to editors and reviewers. We strongly encourage code deposition in a community repository (e.g. GitHub). See the Nature Portfolio guidelines for submitting code & software for further information.

Data

Policy information about availability of data

All manuscripts must include a data availability statement. This statement should provide the following information, where applicable:

- Accession codes, unique identifiers, or web links for publicly available datasets
- A description of any restrictions on data availability
- For clinical datasets or third party data, please ensure that the statement adheres to our policy

The mass spectrometry data ,AA sequence data used for peptide identification and other resources related to peptide identification have been deposited to the ProteomeXchange Consortium with the dataset identifier PXD030145 (https://repository.jpostdb.org/preview/194169514461ab01a6d09da, Access key : 1700).

Field-specific reporting

Please select the one below that is the best fit for your research. If you are not sure, read the appropriate sections before making your selection.

☒ Life sciences ☐ Behavioural & social sciences ☐ Ecological, evolutionary & environmental sciences

For a reference copy of the document with all sections, see nature.com/documents/nr-reporting-summary-flat.pdf

Life sciences study design

All studies must disclose on these points even when the disclosure is negative.

Sample size	For mass spectrometry analysis, we prepared four samples independently for each animal species. For peptide biological assays, we examined five biologically independent samples.
Data exclusions	No data were excluded.
Replication	For mass spectrometry, three samples for each animal species were directly subjected to LC-MS/MS analysis and one sample was further fractionated to reduce complexity. For peptide biological assays, we examined five biologically independent samples.
Randomization	For peptide biological assays, we picked up Cydippid larvae randomly from same batch of the larvae.
Blinding	All measurements in peptide biological assays were collected blind to the identify of peptide sequences and concentrations.

Reporting for specific materials, systems and methods

We require information from authors about some types of materials, experimental systems and methods used in many studies. Here, indicate whether each material, system or method listed is relevant to your study. If you are not sure if a list item applies to your research, read the appropriate section before selecting a response.

Materials & experimental systems

n/a	Involved in the study	
☐	☒	Antibodies
☒	☐	Eukaryotic cell lines
☒	☐	Palaeontology and archaeology
☐	☒	Animals and other organisms
☒	☐	Human research participants
☒	☐	Clinical data
☒	☐	Dual use research of concern

Methods

n/a	Involved in the study	
☒	☐	ChIP-seq
☒	☐	Flow cytometry
☒	☐	MRI-based neuroimaging

Antibodies

Antibodies used	All neuropeptide antibodies were generated by ourself.<br>The following commercial antibodies were used : Alexa488-conjugated anti-rabbit secondary antibody (1:500) (111-545-003, Jackson Immuno Research LABORATORIES), Alexa-488 conjugated goat anti-rabbit IgG (1:500) (Jackson ImmunoResearch), Alexa488 conjugated anti-rabbit secondary antibody (1:500) (A-11008, Thermo Fisher Scientific), anti-tyrosinated tubulin, (1:500) (T9028, Sigma-Aldrich).
Validation	Manufacturer validation statements can be found below for the corresponding antibodies.<br>Alexa488-conjugated anti-rabbit secondary antibody (111-545-003, Jackson Immuno Research LABORATORIES) : https://www.jacksonimmuno.com/catalog/products/111-545-003<br>Alexa488 conjugated anti-rabbit secondary antibody (A-11008, Thermo Fisher Scientific) : https://www.thermofisher.com/antibody/

	product/Goat-anti-Rabbit-IgG-H-L-Cross-Adsorbed-Secondary-Antibody-Polyclonal/A-11008<br>anti-tyrosinated tubulin (T9028, Sigma-Aldrich) : https://www.sigmaaldrich.com/product/sigma/t9028

Animals and other organisms

Policy information about studies involving animals; ARRIVE guidelines recommended for reporting animal research

Laboratory animals	laboratory culture of animals (Nematostella vectensis, Bolinopsis mikado, Vallicula multiformis, and Ephydatia fluviatilis were maintained at the Okinawa Institute of Science and Technology Graduate University. Bolinopsis mikado were cultured also at the Shimoda Marine Research Center, University of Tsukuba.
Wild animals	Bolinopsis mikado were collected at Kamo Bay (Oki island, Shimane), Hakkeijima (Yokohama, Kanagawa), Nanao Bay (Nanao, Ishikawa), and Tabira Bay (Hirado, Nagasaki) in Japan. Bolinopsis collections were done by gently surrounding a sample with a long (2-5 m) griped plastic cap. Neuroepetides were extracted from adult Bolinopsis. For neuropeptide assays, we didn't use wild animals. Vallicula multiformis were collected at a sea-grapes farm Kiyoshi-Hiroshi at Ginoza (Okinawa, Japan).
Field-collected samples	Bolinopsis mikado were maintained at 20 °C in a 60 l aquarium with slow water circulation. They were fed artemia in the morning and evening, with two to three feedings of frozen copepod (Pacific Trading and Kyorin) in between. Vallicula multiformis were maintained in the 1.5 l seawater at 25 °C with feeding freshly hatched artemia two times per week.
Ethics oversight	No ethics oversight was required due to the nature of the organisms.

Note that full information on the approval of the study protocol must also be provided in the manuscript.
