# Supplementary material for: Mass spectrometry of short peptides reveals common features of metazoan peptidergic neurons
Source: Nat Ecol Evol. 2022 Aug 8;6(10):1438–48. doi: 10.1038/s41559-022-01835-7 (PMC9525235; doi:10.1038/s41559-022-01835-7)
Supplement: Supplementary file 5 — Zip file containing four supplementary data files: (1) Supplementary_Data_1.docx, structures of B. mikado neuropeptide precursors and their homologues in Ctenophora; (2) Supplementary_Data_2.docx, structures of N. vectensis neuropeptide precursors and their homologues in Cnidaria; (3) Supplementary_Data_3.pdf, dotplots of gene homologues involved in neuropeptide signalling and (4) Supplementary_Data_4.fasta, AA sequences of neuropeptide precursors used for cluster analysis. [file 41559_2022_1835_MOESM5_ESM.zip › Supplementary Data 1.docx]

**Supplementary Data 1** Schematic representations of neuropeptide precursors identified in *B. mikado* and other Ctenophore species. Gray boxes indicate predicted signal peptides. Green letters show neuropeptide identified with mass spectrometry in *B. mikado*. For other Ctenophora species, green letters show predicted neuropeptide regions based on the similarity to *B. mikado*. Red, blue and yellow letters show acidic, basic cleavage sites and glycines as amide donor respectively. Underlines denote putative cleavage sites of Neprilysin endopeptidase.

**FGFa precursors**

*Bolinopsis mikado*

MKVLVFLALATVVLSQNW**RNKMKPFGFGKR**SDEYDDGDYVVVLVGEGTLFQVLVSSKLDSQGVCTECLLNHD

YDGDYINVISSMSFHGKMKRSEIEEEIVKRCGSGQVALQKRSTDELVEILKRCGDGLVIEKREEEEDIVDLE

TRGGRRTFFRRFEEKKKLAELEERKTKENDSLLQRILDEDEWME

**FGLa precursors**

*Bolinopsis mikado*

MAFTKMLFSLNLLLLVSS**RAYEESAGDENNQAFGLG**N**K**DAASPGDNLEDFAQLRKSSEFELAAEEDDQHFAVGTK

*Mertensiidae sp* (sb|563080|)

MMKFTKTLICLTLV**VLVTCRALEEDDQTIGWGR**AEAAEGSMDQGAVEMELAEELEDQHLGLGAGEEIF

*Mnemiopsis leidyi* (ML030511a)

MALTKILFSLSLILMVSS**RAFEESAGDENNQAFGLG**N**K**DGAVGDSLEEITQLGKVDEFKLAEAEDDQHFAVGT

*Vallicula multiformis* (sb|394883|)

MALTKILFSLSLILMVSS**RAFEESAGDENNQAFGLG**N**K**DGAVGDSLEEITQLGKVDEFKLAEAEDDQHFAVGT

**FQNa precursor**

*Bolinopsis mikado*

MLLVVLALFMALCSAAPNVEIANEVDQEMKRDLKEQLTAKEEEFLIPAIHNIIHGTAHAATTLMHNLLIGKE

KREAPEALTA**RDWLTRDNYRHFQNG**M**K**EIEKREAPEALTAKEEEFLIPVIHNIIHGTAHAATTLMHNLLGKE

KREAPEALTARDWVRLLKNETFLTES

**LNSa precursors**

*Bolinopsis mikado*

MKCFVISVLSLVVLAHVVLADDDGIEDDLTFDERQSLQRRMDSIQDNDESDVEKRE**RGWLKSSRVLNSG**Q**E**E

VLLKRLVDDLEKTAFKK**REQGWAVKNPRLKKGE**LLEKRMDEGEEEEESDVEK**REQGWAVKNARLRKGEE**LEK

RMDQAEEILDLE**KRQLNKPNPRFSKFG**QTRL

*Bolinopsis ashleyi* (comp19971_c0_seq8)

GWQNKNKKLRSGAQEELEMLVEKDESSFDAIDDDKELVASKVEQ**RDLEWRGRKTLLNSGEE**VLEKRLEESEE

DDDSGMEKREQELEKRLDNEFKEDDDSDVDK**RELGWRSKNHKLISG**N**E**EVLRLKKRQ**LNKPNRKFSRFGR**LR

EE

*Bolinopsis infundibulum* (comp67377_c0_seq1)

MSVIRYFNEENISIRRKKLKGAGGKMKKMQVAKLAKGRKGEVELEEELLVERADEFESWVKRLEDEIDNIQV

EEESDIDTEKR**E**Q**GWKTYGKIKKGEE**EVLELE**KRQMNPSNKGFGRFGDE**

*Mertensiidae sp.* (sb|568406|)

EDEIDNIQVEEESDIDTEK**REQGWKTYGKIKKGEE**EVLELE**KRQMNPSNKGFGRFGDE**

*Mnemiopsis leidyi* (ML218828a)

MKCFAVSVISLVVLIHVVRADDDGFEDDLTLDERQTLRRNKKIYRSNRRGRGRNGEVERDVKVLVKRDESLQ

RRMDDVEDDSQSDVAKRE**R**G**LGWWKGSRVLNSG**Q**E**EVLLKRLIDDLEKSAFEK**REQGWTSRNKKSNRAGEE**L

EKRMDEAQGEEESEVEK**REQNWASKNTRLNRGEE**LKKRMDEIQGEEEVEK**REQGWASKNQRLNRGEE**LEKRM

DEAEGEEESEVEK**REQSWASRNEKLNRGE**QLEKRMEETQQVRELE**KRQLNKPNRGFRRFG**QARL

*Pukia falcata* (comp47476_c0_seq1)

GNVELQKLEKRLDNEFKEDDDSDVDK**RELGWRSKNHKLNSG**NEEVLRLK**KRQLNKPNRKFSRFGR**LREE

*Vallicula multiformis* (sb|464413|)

MKCFAVSVISLVVLIHVVRADDDGFEDDLTLDERQSLQRRMDDVEDDSQSDVAKRE**R**G**LGWWKGSRVLNSG**Q

EEVLLKRLIDDLEKSAFEK**REQGWTSRNKKSNRAGEE**LEKRMDEAQGEEESEVEK**REQNWASKNTRLNRGEE**

LKKRMDEIQGEEEVEK**REQGWASKNQRLNRGEE**LEKRMDEAEGEEESEVEK**REQSWASRNEKLNRGE**QLEKR

MEETQQVRELE**KRQLNKPNRGFRRFG**QARL

**NPWa precursors**

*Bolinopsis mikado*

MKQRVTILLTILGVIVLAQSKSILVESEDVLLGEAEELEVDSNSVDLDE**E**L**IGSDIKLVPGAGGNPWGRK**

*Beroe abyssicola* (comp11338_c0_seq3)

MKLIVQIVLFLTVFFCLSQSKSIMSDTEAGVIQDSAEIEDSNSINLE**EELIGTDIKMSPNANMWGKK**

*Beroe sp.* (comp9034_c0_seq2)

MQVLLFVLGFFYLSQSKSIALESTVELRESAEIKETDNLNLEE**E**S**IGTDIKLSPNANPWGKK**

*Mnemiopsis leidyi* (ML215411a)

MKQRITILLTILGVIVLAQSKSILVESEDVLLGDAAELEVDSNSVDLDE**E**L**IGSDIKLVPGSGGNPWGRK**

*P. bachei-pileus* (comp42771_c0_seq1)

MKRVLCVFLLVATVMHFSNAKSVLTEDNDSVVERSAEEIINSVEDNMNLEEE**E**L**IGSDIKMSPNENMWAGRK**

*Vallicula multiformis* (sb|431473|)

MKQRITILLTILGVIVLAQSKSILVESEDVLLGDAAELEVDSNSVDLDE**E**L**IGSDIKLVPGSGGNPWGRK**

**NVFa and NIFa precursors**

*Bolinopsis mikado NVFa*

AKEEEFLLHAIHNIVHGTAHAATTLMHNVFGKRDTSEALTAKE**EEFLINAIHHIIHGTAHALTGVMHGVFGK**

**E**KRDAPEALTAKE**EEFLIGAIHNIIHGTAHAATNLMHNVFGKR**DTSEALTAKE**EEFLLHAIHNIVHGTAHAA**

**TNLMHNVFGKR**DTSEALTAKE**EEFLLNAIHGIIHGTAHAATNLMHNVFGKE**KREAPEALTAKE**EEFLLNAIH**

**GIVHGTAHAATTLMHNVFG**

*Bolinopsis mikado NIFa*

RDANQALSAKEEEFLLNTIHGIVH**GVAHTGTNLMHNIFGK**QKRDTEAVKAKEEDFLLNTIHGIVHGSAHAAT

NLMHNIFGKQKRDANQALSAKEEEFLLNTVHNLVHGIAHAGTNLMYNVLGKRDVSQALTAKEEEFLLNAVHN

LVHGVAHAGTHLMWDVMGKEKREAPEALSAKE**EEFLLNTIHGIVHGVAHTGTNLMHNIFGK**QKRDANEALSA

KE**EDFFRLIHGMVHGLFGK**VKRDANQALSAKEEEFLLNTIHGLVHGVAHTGTNLMHNIFGKRDASQALTAKE

EEFLLNAVHNVVHG**VAHAGTNLMHNIFGK**RDASQALTAKEEEFLLNAIHNVVHGAAHAGTHFMYDVLGN

*Bolinopsis_ashleyi* (comp4270_c0_seq1)

NAEYGETGEAL**SAKDEEFIFKALHNFFGKR**EAPEALSAKEEEFIIPTIHNIVHGAAHTATNLMHNLLLGK

*Coeloplana_astericola* (comp16168_c0_seq1)

KEKRDANQALSAKE**EEFLLNAIHSIVHGTAHAATNLMHNVFGKE**KRDANQALSAKEEEFLLNAIHSIVHGTA

HAATNLMHNVF

*Mnemiopsis leidyi NVFa/NIFa1* (ML40299a)

MLVVILALFVLVSARPNLDETNAEKLSTREEGSVWKAIKSGIAKAKNDSKKYEKYNKYHGYYYIKPQPVHHT

VYTVKVIGKMKREISQALQAKDE**E**W**FTDALHAVAHGATSALLNHFGK**QKRDAPEALSAKEE**E**W**FTDALHAVA**

**HGATSALLNHFGK**QKREAPEALSAKEE**E**W**FTDALHAVAHGATSALLNHFGK**QKRDAPEALSAKE**EEWLSGLI**

**HAAAHGATSALIDHFGE**QKRDASEALSAKEKSALLSKLNAEIAKEVEVVKRDAPENLSARE**EEFLLNTIHGI**

**VHGTAHAATTLMHNIFGKE**KRDTDQALSAKE**EEFLLNAIHSIVHGTAHAATNLMHNVFGKE**KRDANQALSAK

E**EEFLLNAIHSIVHGTAHAATNLMHNVFGKK**RETQTRLLVLKK**EEFLLNAIHNIVHGTAHAATNLMHNVFGK**

**E**KRDANQALSAKE**EEFLLNAIHNIVHGTAHAATNLMHNVFGKE**KRDANQALSAKEEEFLLNAIHNVIHGVAH

AGTHLMWNVLGKEE

*Mnemiopsis leidyi NVFa/NIFa2* (ML16906a)

MLVVVLALVSLASARPNLDQTNTEELSTREEGSIWKAVKSGIAAAKNDSKNYEKLYGYKKHFKYHGYYYIKA

PPLNHTVYQMKVIGKMKRDISEALLAKDEEWFTDALHAVAHGATSALISHFGKQKRDAPEALSA**KDEEWFTD**

**ALHAVAKGATSALINHFGK**QKRDAPEALSAK**DEEWFWHAVAKGATSALLNHFGK**QKRDTFEALSAKDEEFLL

DAIQKFTNKAARAN

*Vallicula_multiformis* (sb|500207|)

AKGATSALISHFGKQKRDAPEALSAK**DEEWFTDALHAVAKGATSALINHFGK**QKRDAPEALSAKDEEWFTDA

LHA

**NVRa precursors**

*Bolinopsis mikado*

MDSLHGTFIIIAYFVHFISG**SPVLKPLDDPVNVRGER**GVTSSHEVQLTSRNNDHVLCPGACSPGLKCAEYGHVALCY

*P. bachei pileus* (comp41957_c0_seq5)

MIKVVFLLLIVGLTVTGS**KEVSRSAPDILELLNAAGEE**NVDAQMKKRGSSSCPDACASGLRCAEYGHVALCY

**PARa precursors**

*Bolinopsis mikado*

RGGGLSEEMQMPSRGGGRSEEMMQTPLRGRNTEENLVPLRRLSEELNMDDLASQEMEEADK**DEVPLDLDSPM**

**VPYNRPARG**

*Bolinopsis infundibulum* (comp41996_c0_seq2)

MQMPSRGGGLSEEMQMPSRGGGLSEEMQTPLRGKNTEENFVPLRRMSEEVDMDDIALREMEAPW**ETYDVVPF**

**KSQASAKKRPARG**

*Mertensiidae sp.* (sb|534219|)

ITAGGGGLSEEMQMPSRGGGLSEEMQTPLRGKNTEENFVPLRRMSEEVDMDDIALREMEAPW**ETYDVVPFKS**

**QASAKKRPARG**

*Mnemiopsis leidyi* (comp5548_c0_seq1)

AITAGGLSEEMQMPSRGGGRSEEMMQTPLRGRNAEENFVPLRRMSEELNNDDLALQEMNEAAQ**DEVPLDMDN**

**PIVPYNRPARG**

**RWFa precursors**

*Bolinopsis mikado*

MKATLLVLTLMIICCNFVQSVPMSQEENLSDEEHRGLQ**KRSGTKFNKADYKSVGEGTRRWFG**

*Beroe abyssicola* (comp8740_c0_seq3)

VWLPYGTAKLSTEAIDSKAIDSKAKDTMALDE**RKGKAFKMKDYKVYREGYRRWTG**

*Coeloplana astericola* (comp56818_c0_seq1)

SDSESQIQ**KRAPKFKKSDYIGVAEGNRNWVG**

*Mnemiopsis leidyi* (ML233326a)

RLSFTE**KRAGTKFNKADYKSVGEGTRKWFG**

*Vallicula multiformis* (sb|377272|)

RLSFTE**KRAGTKFNKADYKSVGEGTRKWFG**

**TFQa precursors**

*Bolinopsis mikado*

MWRETLVVLCLVIFVSARSLSEEDIGHGFAL**D**N**AEEDQLKSGTFQG**LAEDDIGHEFKLDVEEEASIIGHGLSLENVRV

*Bolinopsis ashleyi* (comp19060_c0_seq8)

TQSTGSLKSENIGHGFVL**D**S**ADEVDVKSGTFQIG**LVEKEIGHGIDLHANEEAEIGHGLSLDNVRQF

*Mnemiopsis leidyi* (comp12939_c0_seq1)

MWCKTLVVMCLVFFVSARSLSKEDIGHGFAL**D**N**TEAERLKSGKFQGR**AENDIGHEFKLDANEEASVIGHGLSLENVRV

**VWYa precursors**

*Bolinopsis mikado*

MKCFVVLVALLVLSQSASLNSRESVDDVIMADNNNVELEDGALNAE**EEARVYKGYNGGNRVWYG**

*Beroe abyssicola* (comp15008_c0_seq1)

MKCFVVLVSLIVLSQSASLNSRESENVEDMMMEEDIMGDSADNVELAEE**EEARVYKGYNGGNRVWYG**

*Coeloplana astericola* (comp41350_c0_seq1)

WPLRPGFTLVVVSHSASVIERRDAAVESRSELSEESDDNALYDE**E**A**ARVYKGYNGGKRVWYG**

*Bolinopsis ashleyi* (comp18052_c0_seq5)

MKCFIVLVALLVLSQSASLNSRDSVEDVIIEEHETVDLAEGAMNSE**EEARVYKGYNGGNRVWYG**

*Mnemiopsis leidyi* (ML02736a)

MKCFVVLFALLALSQSASLNSLESVEDVIMADNDNVELEEGALNAE**EEARVYKGYNGGNRVWYG**

*Vallicula multiformis* (sb|434178|)

MKCFVVLFALLALSQSASLNSLESVEDVIMADNDNVELEEGALNAE**EEARVYKGYNGGNRVWYG**

**WTGa precursors**

*Bolinopsis mikado*

MKLFLFVLLGLVALISCEDVESELDTELSESEDASAMRV**KRAKFSMSNYRGHKQGNRGWTGG**AMQEEE

*Bolinopsis ashleyi* (comp19017_c0_seq4)

MKLFLFVLLGLVAAISCESVESELNSELSDSEDASAMRV**KRAKFSMSNYRGHKQGNRGWTGG**AMQEEE

*Mnemiopsis leidyi* (ML02212a)

MKLFLFVLLGLVALISCETVESEVDSELSESEDSNAMRV**KRAKFSMSNYRGHKQGNRGWTGG**AMQEEE

*Vallicula multiformis* (sb|384275|)

MKLFLFVLLGLVALISCETVESEVDSELSESEDSNAMRV**KRAKFSMSNYRGHKQGNRGWTGG**AMQEEE
