# Supplementary material for: Mass spectrometry of short peptides reveals common features of metazoan peptidergic neurons
Source: Nat Ecol Evol. 2022 Aug 8;6(10):1438–48. doi: 10.1038/s41559-022-01835-7 (PMC9525235; doi:10.1038/s41559-022-01835-7)
Supplement: Supplementary file 5 — Zip file containing four supplementary data files: (1) Supplementary_Data_1.docx, structures of B. mikado neuropeptide precursors and their homologues in Ctenophora; (2) Supplementary_Data_2.docx, structures of N. vectensis neuropeptide precursors and their homologues in Cnidaria; (3) Supplementary_Data_3.pdf, dotplots of gene homologues involved in neuropeptide signalling and (4) Supplementary_Data_4.fasta, AA sequences of neuropeptide precursors used for cluster analysis. [file 41559_2022_1835_MOESM5_ESM.zip › Supplementary Data 2.docx]

**Supplementary Data 2** Schematic representations of neuropeptide precursors identified *N. vectensis* and other Cnidarian species. Gray boxes indicate predicted signal peptides. Green letters show neuropeptide identified with mass spectrometry in *N. vectensis*. For other Cnidarian species, green letters show predicted neuropeptide regions based on the similarity to *N. vectensis*. Red, blue and yellow letters show acidic, basic cleavage sites and glycines as amide donor respectively. Underlines denote putative cleavage sites of Neprilysin endopeptidase.

**FRPa precursors**

*Nematostella vectensis* isoform 1

MYSCMGISLLLILCFKGSYGEESIDLEPGVDKVPEKVEHGEEGSAKYNINTVTLTGNKATGNKVTNDDKR**PP**

**WPPRPGKR**TF**RPQESADTPSIFRPGR**ANLHKR**QGLMFRPGRREDVPKPQIFRPGRREDIPSDDDQLMFRPGR**

**NEEQLFRPGRRSDVGDEQLLFRPGRRSDLEEDQLIFRPGRR**SDVAEEQLFRPGRRSDVPEAFL**DQWTSVRPG**

**R**GGYRMPWTYTGNSVNSHVSQKSHTFRQKAVEQEKKKREV

*Nematostella vectensis* isoform 2

MYSCMGISLLLILCFKGSYGEESIDLEPGVDKVPEKVEHGEEGSAKYNINTVTLTGNKATGNKVTNDDKR**PP**

**WPPRPGKR**TF**RPQESADTPSIFRPGR**SV**HEDQLLFRPGR**ANLH**KRQGLMFRPGRREDVPKPQIFRPGRREDI**

**PSDDDQLMFRPGRNEEQLFRPGRRSDVGDEQLLFRPGRRSDLEEDQLIFRPGRR**SDVAEEQLFRPGRRSDVP

EAFL**DQWTSVRPGR**GGYRMPWTYTGNSVNSHVSQKSHTFRQKAVEQEKKKREV

*Actinia tenebrosa* (XM_031706768.1)

LGLLCLFAVLIQGCFCEEDENVIPNEHNVPEKLEHGEKGTVHYNVDTVKLTGSK**HTKMDDNKNERPPWPPRP**

**GRRNIIPLKAKKALPTIIRPGRSILVSLTGKAKRSYQDRVLRPGR**G**VVPNGSPDQLLRPGREDVPEGPQMLY**

**RPGRDAIPQYLYRPGREVAGPPSLFRPGREDVPQNAFRLFRPGRD**DVPNGPPALFRP

*Anemonia viridis* (GGLT01105026.1)

MACPRKTGLGILCLFAILIQGCFCEEGENLVPNEQGSVPEKLVHGEK**GTVQYKVNTVKMTGNEHVKMDENKN**

**QRPPWPPRPGRRNIIPLSLRAKKTLPTVIRPGRSVIESLTSKAKRAYQDRLIRPGRDNVPKGPQYLYRPGRE**

**DVPEGPQFLLRPGRDVPERPQTLLRPGRGDVPEGPQNLLRPGRGDVPEGPQNLLRPGRGDVPEGPQNLLRPG**

**R**G

*Exaiptasia pallida* (XM_021057432.2)

FVLGILIQGCFVFSEDNIKPDVDRIPEKIEHGEKGKSEYKVNSIILTGNQ**HTQVDNNDEDMRPPSWPPRPGR**

**RSFISRIKSRSSGPTIMRPGRELNSEKSKRMSSPQFVLRPGRGIDQHLAGDERDDVPQHPNILRPGRDKIER**

**PPSLFRPGREDVPQISLLRPGREIKTRLSVLRPGREDVPQISIFRPG**

*Metridium senile* (GGGC01020455.1)

MDRKTSMANSLVIKAVFILVLYMQVSFVLGVEDLQPDSHRVPEKIEHAEKGGSEYNV**NTVTMTGNQHTKVDD**

**DIHVRPPPWPPRPG**R**RSFISIKARNSIPTIWRPG**R**AVKDSISSKAKRKSSPQVFRPNILRPG**R**DAIGDSHPI**

**IFRPG**R**EDVPKRPPILFRPG**RE**DVPQLSSVFKPG**RDK**VPSKPQQPFVLRPG**RE**DVPQLTLLRPG**RE**FARQSV**

**YLFRPG**R**DNVPDPFQHSVVRAG**RRNIRYNMPWTYVGSSVNSHVHQKSHTFHQKAQEELAKRELPQEMSDPSN

HQGLRDF

*Stichodactyla helianthus* (GGNY01029085.1)

MRILFPLGCFAEEDEVVIPNEHSVPEKLEHGEK**GTVQYKVNTIKLTGSQHQKSDEGIPQKPPWPPRPG**RR**NV**

**IPLKAKKALPTIIRPG**R**SILDSLINKVKRNHPQRILRPG**R**EDVPDGPPPRLYRPG**RED**VPGGPPVLLRPG**RE

**DVPQDYPSLLRPG**R**AAPPTLLRPG**RE**DVPQNHLLRPG**RN**DIPDQYLFRPG**RD**DVPDAPPSLLRPG**RE**DVPQN**

**YLFRPG**RNDIPNQFHSVRAGKRSTGYNMPWTYVGSSVNSNLQHNSHTFRQKALEETTKRTAQDDEQEMSDPN

SLLDEQQDLI

**GGYa precursors**

*Nematostella vectensis*

MKTLCLVAGICLLLAVTLLQAQGLSREYEEEIREGLEAAMRFPKSPFDAQAMMRPAKKS**DLPPRFGGYGRR**

*Edwardsiella carnea* (GGGB01050922.1)

MNRTFSLVLGILLVLSITLSTEGSPSYRVEERRRRQFDAAPRVPQEPFDFRSLFEAEKR**EEIPPRFGGMIGRR**

**GLWa precursors**

*Nematostella vectensis*

MALFGHTLVAVLFCLALCHAETKRKAADTTDTENELDASPNVNNDDDNDIKRMPTDTKRQAGAPGLWG**KRDA**

**GPPGLWGKR**DAGPPGLCRKRSPKPPGLWGKRQAGAPGLWGKRSAGPPGLWG**KRDAGPPGLWGKR**VAGPPGLW

GKRQAGAPGLWGREAGAPGLWGKRQAGAPGLWGKREAGAPGLWGR**E**A**NAPGLWGKRRAGAPGLWGKR**EANAP

GLWG**KRQAGPPGLWGKRDEDEDEDMDETNGDPLWGR**SA**DAGPPGLWGRK**KRAASP**QRDLYGIGLWGR**NAALM

TAEELDLSFKNEEQS

*Aurelia aurita* (KC767904.1)

GTWGKRENQPPGTWGKRES**QPPGTWRKREHQPPGTWGKRE**N**QPPGTWGKRENQPPGTWGKRE**N**QPPGTWGKR**

**ENQPPGTWGKRE**N**QPPGTWRKRENQPPGTWGKR**ENQPPGTWRKR**E**N**QPPGTWRKRENQPPGTWGKR**ENQPPG

TWRKRENQPPGTWRKRENQPPGTWRKR**E**N**QPPGTWRKRENQPPGTWGKRE**N**QPPGTWRKRENQPPGTWGKR**E

NQPPGTWRKR**E**N**QPPGTWGKRE**N**QPPGTWGKR**E

*Corynactis australis* (GELM01038038.1)

MSVSHDIVNELSRGNTERKKMPALKVTFFLLLAVLPLCEARHFRKTEDSKTNENLASGKKNEGTVPKTALNS

DEDGQIMHADIRGPGIKRQDDLPGMWGRSLSIQPHNLEPDEVDESSKSLVEQQVVSSSNKKREE**EDGPPGLW**

**GR**SLNKEESL**KDGPPGLWGRE**I**E**H**GPPGLWGRD**L**E**N**APPGPWGR**EL**E**N**GPPGLWGR**EL**K**N**GPPGLWGRE**LKN

**GPPGLWGRE**LKN**GPPGLWGRE**LKNGPP

*Dynamena pumila* (GHMC01023158.1)

MAKLTLLMVSMAVAVVSCIAVDKSTKTSQDQISTLDDENNQKRMLVEEIVKQLVESLHGELKKRSLAPP**KQR**

**PPGLWGRE**IGSSDVLG**RQIEKPPGLWG**V**EIEKPVGLWGREDQKPVGLWGREEQKPGGLWGREEQKGLWGRE**A

**QKGLWGRE**V**QKGLWGR**GA**QKPVGLWGREEQKPVGLWGRE**A**QKPVGLWGR**DAQKTVGLWIRHA**QKPFGVWGRE**

V**QKPVGLWGRD**A**QKPVGLWGREEQKPVGLWGR**QIGKPPGLLGREQRLAAPALWKRSKESTKTEGKPPGMWGK

DTIDERSSDATLKTKVKNDDDSNRTKM

*Hydractinia symbiolongicarpus* (GAWH01054632.1)

RPPGLWGREADFDNNRAHDSAQISDEKPPGIWAG**DAKPPGLWGRDAKPPGLWGRDAKPPGLWGRDAKPPGLW**

**GREAKPPGLWGRDVKPPGLWGRDAKPPGLWGRDAKPPGLWGRD**AKPPSLWSKDNNVIKS**QSEDAKPPGLWGR**

QVEDGPTKIWGDGFLDAERHIRLLKNDER

*Hydra oligactis* (GHUC01004966.1)

MGMFERKKFVLLVSLICVSQQAANIKDANTLSTSTELKVVKPQKRVTPVKDAEKLSILRTQDNSLDLNANRE

EVWNELTQDIPLEYIEGIYNELTRLAHNENRPKRLWGATAAINTENFNPEAENELENKKSEPVVEKFERPIG

LWH**KDIETENPENRLPLGLWGKDSEPLPIGLWGKDSEVNDELNKEPLPIGLWGKD**IDSTQ**EDNKPNPKGKLP**

**IGLWGKD**DAVTHDLR**KKNSGPPPGLWGKDKPIPGLWGKDNAPMPGLWGKKDSGPPPGLWGKKDQPPIGMWGR**

TGKK**D**S**NPYPGLWGKK**EEEIENLNREFNENILEEYPPCLLENPPCEIQVKRYKTE**K**S**GPPPGLWGKR**SEKNT

IN**KPPWRGGMWGR**SAILENSVHDSKQTNNVELKRAEKN

**GQIa precursors**

*Nematostella vectensis*

MQPLSVVIGVFVVLTITVLYTEGSPTGLDEREELYEAFMNARKAPLDIQSLFKLEDKRSAA**LPPRFGGQIGRR**R

*Edwardsiella carnea (GGGB01050922.1)*

MNRTFSLVLGILLVLSITLSTEGSPSYRVEERRRRQFDAAPRVPQEPFDFRSLFEAEKR**EEIPPRFGGMIGRR**

**GTEa precursor**

*Nematostella vectensis*

MFKYSIGLLLAMVMLTHGLPLQELVRDAAPKEAEKQPEKVEDKPEEPAKDEEKEEAATEAEAEDPKEDPEAA

EIPGGTEDLPEDEYSEDEAEM**EEAPATPEGGTEGDE**DALEGGTEEDENALEGGTEEDENALEGGTEGDEDAL

EGGTEGDDDAPEAFDEEEKPEENDMGGTEEENPDVPSDEPLTGGTEEDDEKEASEEEEGKPSLEDGTEDTPE

ANKTPEEPKEDEKVTEEETPKEETPKVEDAPEEVEAPKEEESPKEEDTPKEEDAPKEDTPKEEAKEEP**KEDT**

**KEEPKKIEDLEKLSDIRG**

**HIRa precursors**

*Nematostella vectensis*

MRLYLFVPVFTLVLAVEGASDEKRDS**KQPPIDLSPAAYFHIRGKR**THN**APPLDLSGPAYFHIRGKR**TA**KQPP**

**YLDLGEPSFFHIRGKR**T**EGPPYIDLTEPSFFHIRGKR**SS**EQPPLDLGPAYFHIRGKR**T**KNPPIDLGPAYFHI**

**RGKR**LSG**EQPPYLDLTPAYFHIRGKR**T**QQPPMIDLSEPAFFHIRGRR**AV**EQPPYLDLTPSYFHIRGKR**T**EYP**

**PFLELGQPSYFHIRGRR**AEKTTKD

*Actinia tenebrosa* (XM_031702732.1)

KKEEK**LPPIIDLGQPAYFHIRGKR**E**E**S**RPPLVDLTEPAFFHIRGKR**VA**KQPPFLGQPAFFHIRGKRKENPPY**

**EELIEPAFFHIRGKR**VA**KQPPFLGGPAYFHIRGKR**DV**LPPYVDLTEPAFFHIRGKR**VA**KQPPFLGGPAYFHI**

**RGKREEYPPYIDLTEPAFFHIRGKR**

*Anthopleura elegantissima* (GBXJ01068702.1)

MSRFSLHVLSFALLIAVVIATNEKKEA**KFPPIVDLGQPAYFHIRGKR**E**E**N**RPPLVDLTEPAFFHIRGKR**VAK

QPP

*Exaiptasia diaphana* (XM_021050628.2)

QPAFYHIRG**KRQFPPNVDLTAPAYYHIRGKR**VA**QQPPFVDLTQPAYYHIRGKR**

*Metridium senile* (GGGC01066484.1)

MKYGLWAVTVAMVIAVVIATKENEDA**KEQPPYVDLTQPAFFHIRGKR**VS**KQPPYVDLTAPAYYHIRGKR**AAR

QPPYVDLTQP

*Palythoa caribaeorum* (GESO01069612.1)

MTCGVAILLLTLITSCIGKSTTKTEKRADGLDY**KRQLLPGNVLNGGVAFGHIRGKKQDIPLESLLNGGVAYG**

**HIRGKKQEIPLASLLNGGISYGHIRGKKQEIPLAGLLNGGVAYGHIRGKKQEIPVESILNGDVSYGHIRGKR**

**R**S**YPPYDSILNSELAPFGHIRGKRQFTPGFLSDIQTLGHIRGRKRQFLPASYILGDGVSYGHIRGKRQFPAG**

**SILDGGVSFGHIRGKRQYPINELGGISYLGHIRGKR**TEKDVEE

*Protopalythoa variabilis* (GCVI01053040.1)

MTCGVAILLLTLITSCVGKSITKTLKRADGLDY**KRQLLPGNVLNGGVAFGHIRGKKQEIPLESLLNGGVAYG**

**HIRGKRQQIPLASLLNGGILYGHIRGKKQEIPLAGLLNGGVAYGHIRGKKQEIPVESILNGGVSYGHIRGKR**

**R**S**YPPYDSILNSELAPFGHIRGKRQFTPGLLSEIQTLGHIRGRKRQFLPASYILGDGVSYGHIRGKRQFPAG**

**SILDGGVSFGHIRGKRQYPINELGGISYLGHIRGKR**MEKDVEE

*Stichodactyla helianthus* (GGNY01149012.1)

MRSSLQILSFALLIAVVIATNEKKEAKA**QPIVDLAQPSYFHIRGKREEYPPLVDLTEPAFFHIRGKR**VA**KQP**

**PFLGQPAYFHIRGKRD**V**GPPYIDLTEPAFFHIRGKR**VA**KQPPYEEWGQPAYFHIRGKR**EEYPPLVDLT

*Zoanthus sp.* (GGTW01139254.1 )

**KQEIPLASLMKGNVAYGHIRGKKQEIPLQSILNGGVAYGHIRGKR**SH**LPPYDNRVAFGHIRGKRQNFGPGWL**

**NELQMLGHIRGRKRQFLKPNHFMGSGVAYGHIRGKRQLPFSQLMNSGVSFGHIRGRRQFPIDGLDGISYLGH**

**IRGKR**TEKSI

**IVLa precursors**

*Nematostella vectensis*

MVQRSVLVIVVLLYILVQVESYSWSVKANVNNPKGDSPDDKKIWRSLRESMGAVRRADERR**D**A**RPPQSGHLFEEDRIVLG**H**R**

**LRWa precursors**

*Nematostella vectensis*

MASKTLLVCLLVTFMVLSIYTQESSA**GPPQGLRWGKR**WENPSEKQVRENAEREVQDFKDYFKKKYNRDLDI

*Actinia tenebrosa* (XR_004196721.1)

MACKTYLIILLVSCLLVSMCIQQSNG**QQQGLRWGKK**SVDSLEQEINEEKAADELRRFKDYFKRKYNQD

*Anthopleura elegantissima* (GBYC01037783.1)

MASKTYLITLLVSCLLISVCIQHSNA**QQQGLRWGKK**SVDSMEQQINEEKAADELRRFKDYFKRKYNQDAIF

*Edwardsiella carnea* (GGGD01092893.1)

MASKTLLVCLLVSFMLVTIYTQQSNA**QGAQGLRWGKK**SVDSELEEDQKAEAELRRFKEYFKRKYNRDFTTM

*Exaiptasia pallida* (XR_002334434.2)

MASRTLIAFLLVGCMLVSVVLSQ**KDEWTGMRWGKK**SLQTKETHD

*Scolanthus callimorphus* (GGGE01334502.1)

MASKTLLVCLLISFMVISLYTEQTSA**QSQGLRWGKK**SVDSELEEDQKAEAELRKFRQYFKRKYHRDFAY

*Stichodactyla helianthus* (GGNY01010468.1)

MASKTYLITLLVSCLLISMCVEQISA**QKSALGLRWGKK**SVDSMEQQINEKAADELRRFRDYFKRKYHQDINF

**NQWa precursors**

*Nematostella vectensis*

KE**IPPQGFRFNQWGKKEIPPQGLRFNQWGKKEIPPQGFRFNQWGKKEIPPQGFRFNQWGKKEIPPQGFRFNQ**

**WGKKEIPPQGLRFNQWGKKEIPPQGLRFSQWGKKEIPPQGFRFNQWGKKEIPPQGLRFNQWGKKEIPPQGLR**

**FNQWGKKEIPPQGLRFSQWGKREIPPQGLRFNQWGKKEIPPQGLRFNQWGKKEIPPQGLRFSQWGKKEIPPQ**

**GLRFNQWGKK**EIPPQGLRFNQWGKRKLINQVMI

*Haliclystus auricula* (HAHA01038764.1)

QPPGVWG**K**M**ENQPPGVWGKRENQPPGVWGKRENQPPGVWGKRENQPPGVWGKRENQPPGVWGKRENKRENQP**

**PGVWGKRENQPPGVWGKR**EN

*Haliclystus sanjuanensis* (HAHB01030545.1)

R**ENQPPGVWGKRDNQPPGVWGKRENQPPGVWGKRDNQPPGVWGKRENQPPGVWGKRDNQPPGVWGKRENQPP**

**GVWGKRENKKENQPPGVWGKRENQPPGVWGKRENQPPGVWGKRENQPPGVWGKRENKRENQPPGVWGKRENQ**

**PPGVWGKRENKRENQPPGVWGKRENQPPGVWGR**

*Hydra vulgaris* (XM_002164712.2)

GLWGKDADVNDDLKK**EPLPIGLWGK**DTDSTRGDNKPNAYKGK**LPIGLWGK**DNALTNDLGKKNNGKDS**GPPPG**

**LWGKDSKPIPGLWGKDNGPMTGLWGKKDVGPPPGLWGKKDQPPIGMWGR**AGKR**DSNPYPGLWGKK**EEELENV

DKEIEEDSLEEFPACLLENPPCEIQEKRYNID**K**S**GPPPGLWGKR**SEKYQMN**KPPWRGGMWGR**SEI

**PRGa precursors**

*Nematostella vectensis*

MAYFKWTLCAFVLAVLCLSTNYVAGEKNEEKSEKSTLLQELSKKDFDKELAADEAIVLELLKEKADDLDEES

**LLDLLAPRGGRD**APRGGRSLLDAPRGGRSLLDLLAAPRGGRDAPRGGRSLTELLNAPRGG**RSLADLLDAPRG**

**GR**SLAELLDAPRGGRSIEAPRGGRSLAELLEAPRGGRSLIELLEAPRGGRSVEAPRGGRSILELLTAPRGGR

SAPRGGRSAEKSDAIHKEKKAPRGGRKR**R**S**LPEEEMDGPRGGR**SAVSGRSSEDGPRGGRAVYGPRGGRSFDG

SRGGRSYDGPRGGRSM**E**N**GPRGGRATEFGPRGGRSYEGSRGGREIDGPRGGRSFKEGPRGGR**AVEGPRGGRD

LYEEGPRGGREVYLEGPRGGRDLVLEGPRGGRDLYEEGPRGGREVNLE

*Acropora tenuis* (IADL01073014.1)

MTFNSLKLLLLASLFTVLMLKERRVLADGQDQKAVAKRDEVLDGNVESVKNSYSIEGDGKSRRSAEDEENQG

EINDDGMMYYGSFYPMDMNTKRYELYYPENPPYEEWYGPFEPP**EEYENQGEWYEGNGYYKRSLDFLGPRGGR**

**SVYYGPRGGRSLENEAGPPNGRSLNDEIMGPRGGRTVGTVQGPRGGRSVENGGPRGGRSVDTLAKGRLGVED**

**SEEKKSTTRTVKKSDTSSINGPRGGREIKADVGPRGGRGVSDSKTTDDVNYGTPGRRDARSASGPRGGRDAT**

**ERDSREERERSVLDFLGPRGGRSIEFGPRGGRSTIFESSRLWDERAFDYGPRGGRYIDYVLRRRSLETPPLG**

**RRDIVFGPRGGRGILSGSRGGRAIDYGPRGGRSLEGYCPHCARSINFAEYGPRGGRSIEMGPRGGRSVDFGP**

**RGGRSLSQGPRGGRSLLFNTFAFSGPRGGRSVNYDLYEGPRGGRAIDERELSRAFGNEGDVYYYGPRGGRSV**

**YVETGPRGGREVEYTGPRGGRAVIYGPRGGRAIFSGPRGGRDISGPRGGRNIESGLRGGSSTKSDFGPRGGR**

**SILSESAMWSKTGPRGGR**SLESSGSTVESSATEKREATSSVERQTTSNEKVRKERDTNVDMEKIDKKVTKSN

*Acropora millepora* (GHGM01068906.1)

AEDEENQGEITDDGMMYYGSFYPMDTNTKGYELYYPENPPYEEWYGPF**EPPEEYENQGEWYEGNGYYKRSLD**

**FLGPRGGRSVYYGPRGGRSLENEAGPRGGRSLNDEVMGSRGGRTIGNAQGPRGGRSVENGGPRGGRSVDTLA**

**KGRLGVEDSEENKSTTRTFKKSDTSSIKGQRRGG**

*Anemonia viridis* (GHCD01115618.1)

RGG**RSMEDGPRGGRSMEEDGPRGGRSMEDGPRGGRSMENDGPRGGR**SMEDGPRGIIHTSKEVIIVISSCFLG

QLVQKM

*Clytia hemisphaerica* (KX496947.1)

MERKILACLFLLIVLLNLNDGKNIAILIEPDDNLASELEWLGSDMTDSHSLNAGAW**PRPGDARSSHDAWPRP**

**GKREFYGNEMFEKRPFPGQQMQFSWPRPGKKETKEDTWPRPGKRESYSEGDMDSRSGALRRSEEKETNEDEK**

**LENAWPRPGKREFYASRKMDVRPRGGRDSKSHKISKRNSEAISNDEIDMMIREEAWPRPGKRDYHMLSATRP**

**RGGKDARPRGGKDSSRPRGGKNAKPRGGKDSVRPRGGKKDSWPRPGKDAFVKEINGSRPRGGKDASKWPRPG**

**KK**DLK

*Tripedalia cystophora* (MH423433.1)

EQPRSGKREL**SAQDYRPRAGREGDQDYRPRAGRQVLTRPRGGREYSERPRAGREYTIKIISSESQSYGRPRA**

**GRESAARPRAGRENLERPRAGRESLVRPRAGREDIERPRAGREDLERPRAGREDLERPRAGRQMVGRPRAGR**

**EFFERPRAGR**NVVLI

**PRPa precursors**

*Nematostella vectensis*

MKVLCVTLVLMALVCSVQSRNLRHKDYERELQDYRRDFARALSMV**EQDAFLPKPRPGRREQDSSNYEFPPGF**

**RPGKK**RSFKEEK

*Anemonia viridis* (GHCD01048248.1)

MASLNVVLFSALLALLVVTCYGFRLQKDLESEYEGNSKEYE**D**S**QKAIPVRPGKR**EFNRMLEDSPKAIPVRLS

KRGYPLHRMSE**D**S**QKAIPVRPGKR**EFNRMLEDS

*Edwardsiella carnea* (GGGB01122376.1)

MRSCNGLFCAAVAALLLVTTQCYRLQNVQADEFRTELSDEN**D**S**GPPISNIPGVRAGKR**GFQYSSSS**D**S**QGPP**

**IVYLPLRPGKR**

*Scolanthus callimorphus* (GGGE01181110.1)

MNYHKGFFVVLIATLLLATAQSYRLSSLEESEDGPPISNNNPVRLGRREFDPSEPQAPSMERIPIRNGKRRF

NLNDF**E**A**GLPMEKIDFRPGKR**FKSFLD**D**P**QSPPAQYGPVRPGRR**SFAPDVA**GPPMGNLNIRPGRR**DLAPKDV

V**GPPMGNFHIRPGRR**EFTY

**RFa precursors**

*Nematostella vectensis*

MAPRPGTLLLLGILIQVLICTAKSTYKKEIADLLDDNKDTPQFWKGRFSDPQFWKGRFSDPQFWKGRFSDPQ

FWKGRFSDPQFWKGRFSDPQFWKGRFSDPQFWKGRFSDPQFWKGRFADELLNGGHHKHHHEEGEWKRTAGPG

RFGR**EDQGRFGREDQGRFGREDQGRFGREDQGRFGREDQGRFGREDQGRFGREDQGRFGREDQGRFGREDQG**

**RFGREDQGRFGRED**QGRFGRNKIARVIDLDQGRFGRTMDTATKKDTVAS**MPEQDANPQTRFDGKK**RQAAKER

SIEKKSTISSDAKASDAKQS

*Actinia tenebrosa* (XM_031703761.1)

KRELSSTLKEPSDDEPQFWKGRFSDPQYWKGRFSDPQFWKGRFSDPQYWKGRFSDPQFWKGRFSDGTKRENS

PQYWKGRFSRSFNGRPDSDPQYWKGRFSRGSVPGRYGREQGRFGR**E**L**QGRFGREFQGRFGREDQGRFGREDQ**

**GRFGREDQGRFGREDQGRFGRE**L**QGRFGRDFQGRFGREDQGRFGREDQGRFGREDQGRFGREDQGRFGREDQ**

**GRFGREDQGRFGRE**L**QGRFGRELQGRFGRE**L**QGRFGREFQGRFGRE**D**QGRFGREDQGRFGRED**Q**GRFGREDQ**

**GRFGRE**N**QGRFGREDQGRFGR**QDLSEKEQGSFGSEELAEKDQGSFDENAAEEKKKTIDVI**D**I**ESDPKPQTRF**

**RDGKE**TQEKRKVEKKDKIE

*Anthopleura elegantissima* (M99170.1)

SDAKATNNKRELSSGLKERSLSDDAPQFWKGRFSRSEEDPQFWKGRFSDPQFWKGRFSDPQFWKGRFSDPQF

WKGRFSDPQFWKGRFSDPQFWKGRFSDPQFWKGRFSDGTKRENDPQYWKGRFSRSFEDQPDSEAQFWKGRFA

RTSTGEKREPQYWKGRFSRDSVPGRYGR**E**L**QGRFGRELQGRFGRE**A**QGRFGRELQGRFGRE**F**QGRFGREDQG**

**RFGREDQGRFGREDQGRFGREDQGRFGREDQGRFGREDQGRFGRELQGRFGREDQGRFGREDQGRFGREDQG**

**RFGRELQGRFGREDQGRFGREDQGRFGREDLAKEDQGRFGREDLAKEDQGRFGREDIAKEDQGRFGR**NAAAA

AKKRTIDVI**DIESDPKPQTRFRDGKD**MQEKRKVEKKDKIE

*Calliactis parasitica* (M59166.1)

INAKTVTKRAKETNLEDDEPQYWRGRFAKDVVPQFWKGRFSDPQFWKGRFSDPQFWKGRFSSHGNKRRYVPG

RYGREFQGRFGR**E**F**QGRFGREQGRFGR**E**EDQGRFGREEDQGRFGREEQGRFGREEDQGRFGR**E**EDQGRFGRE**

**EDQGRFGR**E**EEQGRFGREEDQGRFGR**E**EEQGRFGREEDQGRFGR**E**EDQGRFGREEEQGRFGKR**D**EDQGRFGK**

**REDQGRFGKR**D**EDQGRFGKRDEDQGRFGKREDQGRFGKREDQGRFGRE**LLAKLNKRTTSIQEDPQTRFRDVQ

MTRRNVAKKDKIEESNDEEAN

*Exaiptasia pallida* (XM_021056601.2)

MTTASYVTILVTLLFHILAINAKDTKREPEDDQPQFWKGRFARGAVPQYWQGRFSDPQFWKGRFADPQFWKG

RFADPQFWKGRFADPQFWKGRFSDPQYWKGRFSDDDKRSNDPQYWKGRFSRSMKTPDDDLPQFWKGRFSRDS

LPGRFGR**E**L**QGRFGREQGRFGREEQGRFGREEQGRFGREEQGRFGREEQGRFGREEQGRFGREEQGRFGREE**

**QGRFGREEQGRFGREEQGRFGREEQGRFGREQGRFGREEQGRFGREDQGRFGREEDQGRFGR**E**E**A**DQGRFGR**

**EEEDQGRFGR**EK**EDQGRFGREEADQGRFGKR**DLKAKSNSKRTIEVQSDAMPQTRFRDSFKRNLVKKEKKSAD

ASSKAT

*Pocillopora damicornis* (XM_027195762.1)

CHLLLVHAKALEDSTKEAADENDVPAFAEGKFTRSLENDPQYWKGRFSDIVGELSDPQYWKGRFSHDQYWQG

RFADTGSEMDKREPQYWKGRFSRGE**EEQQLRSAVPGRFGR**NF**QGRFGRNFQGRFGR**NF**QGRFGRELQGRFGR**

**DEIQGRFGREDLQGRFGRE**DMQ**GRFGREEEDQGRFGRD**FI**QGRFGREDQGRFGR**E**DDQGRFGRDSIQGRFGR**

EEL**DQGRFGREELDQGRFGRDEIVEDEDQGRFGRE**E

**VRHa precursors**

*Nematostella vectensis*

MKITPVMSCLVLASALLVTAEC**YRITDPGDLEKPQENDEPGPPMIKIPVRHGKR**EFNDDLEDTTLLHYPFRV

AR**KRELFNAGGPPPIYLPVRPGKR**GYYRDNIQETRQYLDPRPGKRSRNH

*Anemonia viridis* (GHCD01048248.1)

MASLNVVLFSALLALLVVTC**YGFRLQKDLESEYEGNSKEYEDSQKAIPVRPGKR**EFNRMLEDSPKAIPVRLS

**KRGYPLHRMSEDSQKAIPVRPGKR**EFNRMLEDS

*Edwardsiella carnea* (GGGB01122376.1)

MRSCNGLFCAAVAALLLVTTQC**YRLQNVQADEFRTELSDENDSGPPISNIPGVRAGKR**GF**QYSSSSDSQGPP**

**IVYLPLRPGKR**

*Scolanthus callimorphus* (GGGE01181110.1)

VVLIATLLLATAQS**YRLSSLEESEDGPPISNNNPVRLGRREFDPSEPQAPSMERIPIRNGKR**RFNLNDFEAG

LPMEKIDFRPGKRF**KSFLDDPQSPPAQYGPVRPGRR**SFAPDVAGPPMGNLNIRPGRR

**WFSa precursors**

*Nematostella vectensis*

MSNYFLHWVTALAFILCSGLANG**KIPKVPIKEVENAPPSTGEGEAGFIPWFSGKREFNPKNALKNDNAPPGN**

**EEGEAGFIPWFSGKREFEPKNALQNDNAPPGNGEGEAGFIPWFSGKREFEPKNALQNDNAPPGNGEGEAGFI**

**PWFSGKR**DTTKAAV**QNDLVPPSKGAGEQGFIPWFSGKRTVNRLKEINTENDADANKAPPSGHNEAGFIPWFA**

**GKR**KV

*Edwardsiella carnea* (GGGB01072687.1)

MRNFNVLSLLAFVIALFFLMSLSFMRVNGAD**LKRRAPPGQGEGEARFIPHFSGKR**ELGEKSAERKDEMRGYQ

AYTRDDAEFPWQRQ
