# Supplementary material for: Mass spectrometry of short peptides reveals common features of metazoan peptidergic neurons
Source: Nat Ecol Evol. 2022 Aug 8;6(10):1438–48. doi: 10.1038/s41559-022-01835-7 (PMC9525235; doi:10.1038/s41559-022-01835-7)

Neuropeptides

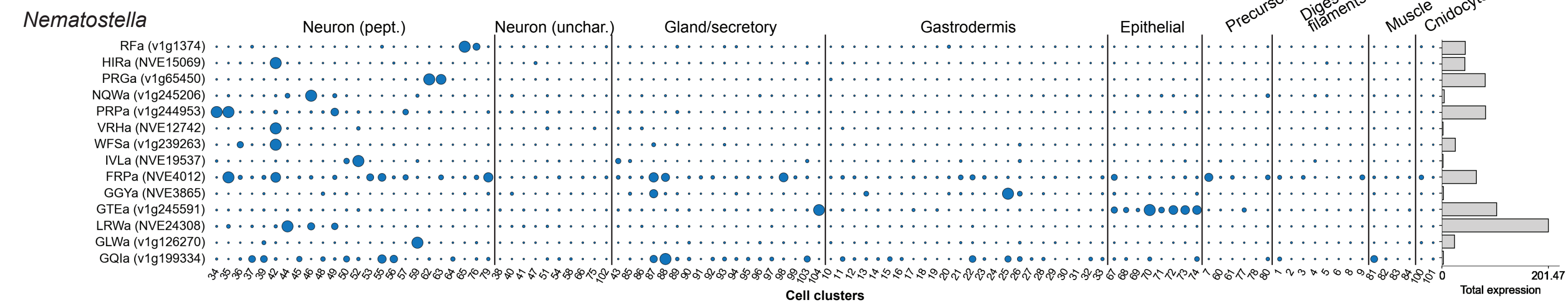

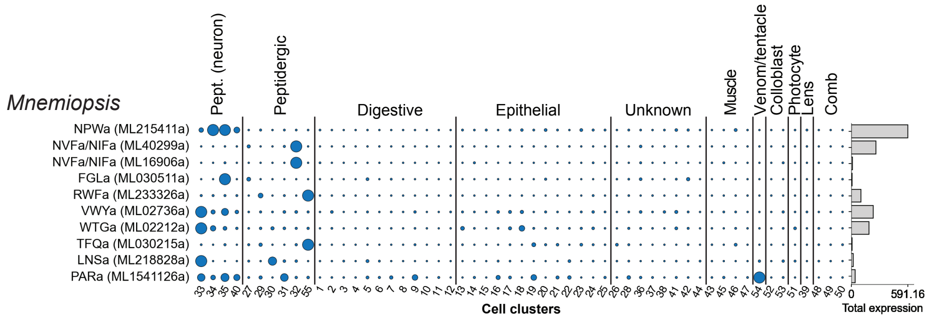

Peptide processing (PC1/2/3/Furin-like proteins)

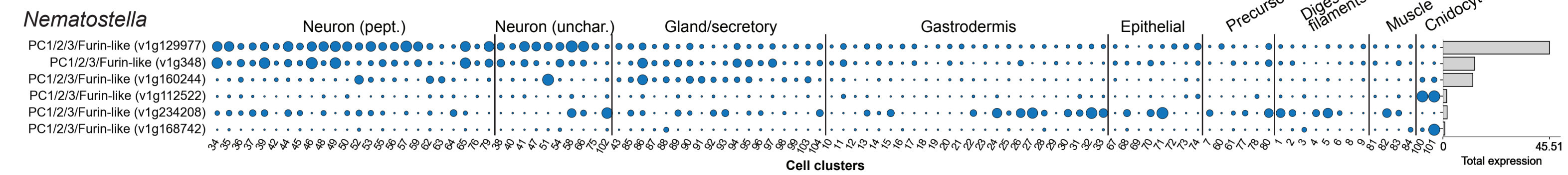

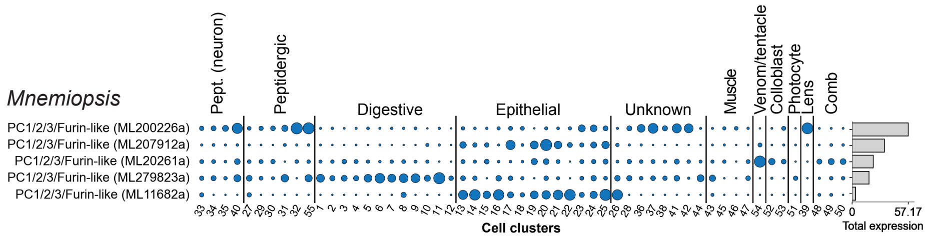

Peptide processing (Carboxypeptidases)

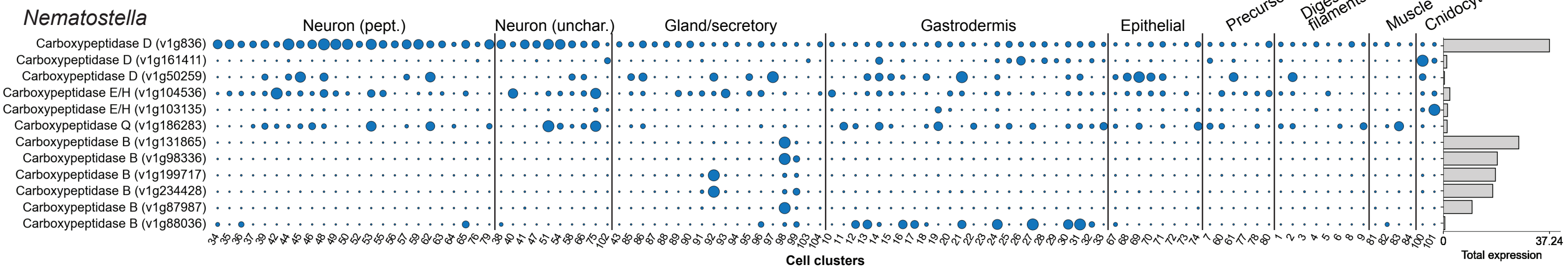

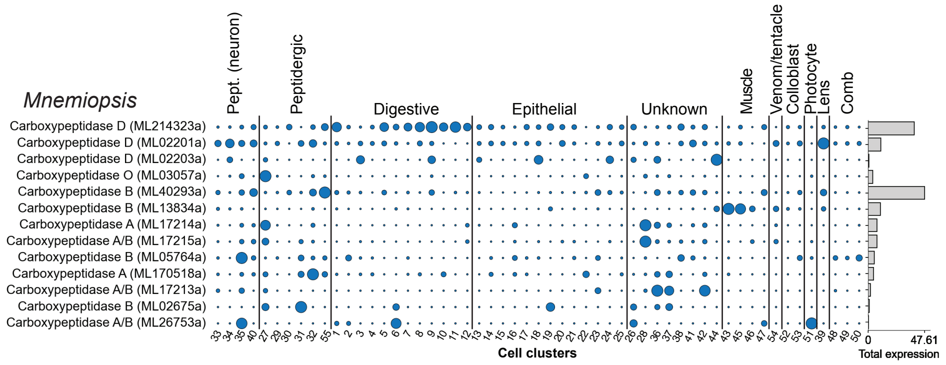

Peptide processing (APN/EAP)

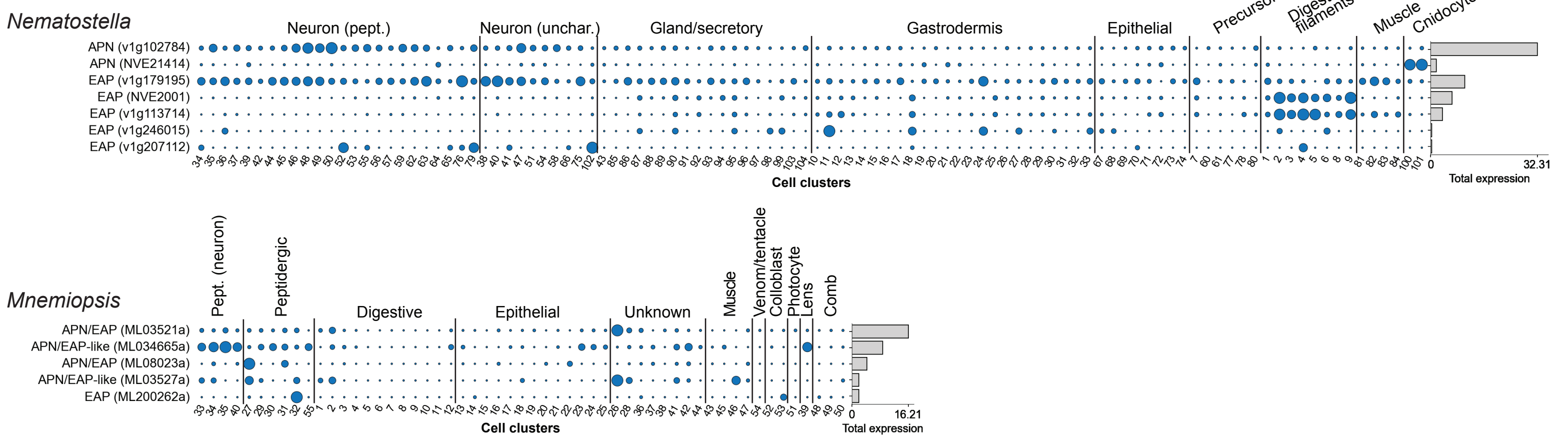

Peptide processing (GCP2, QC)

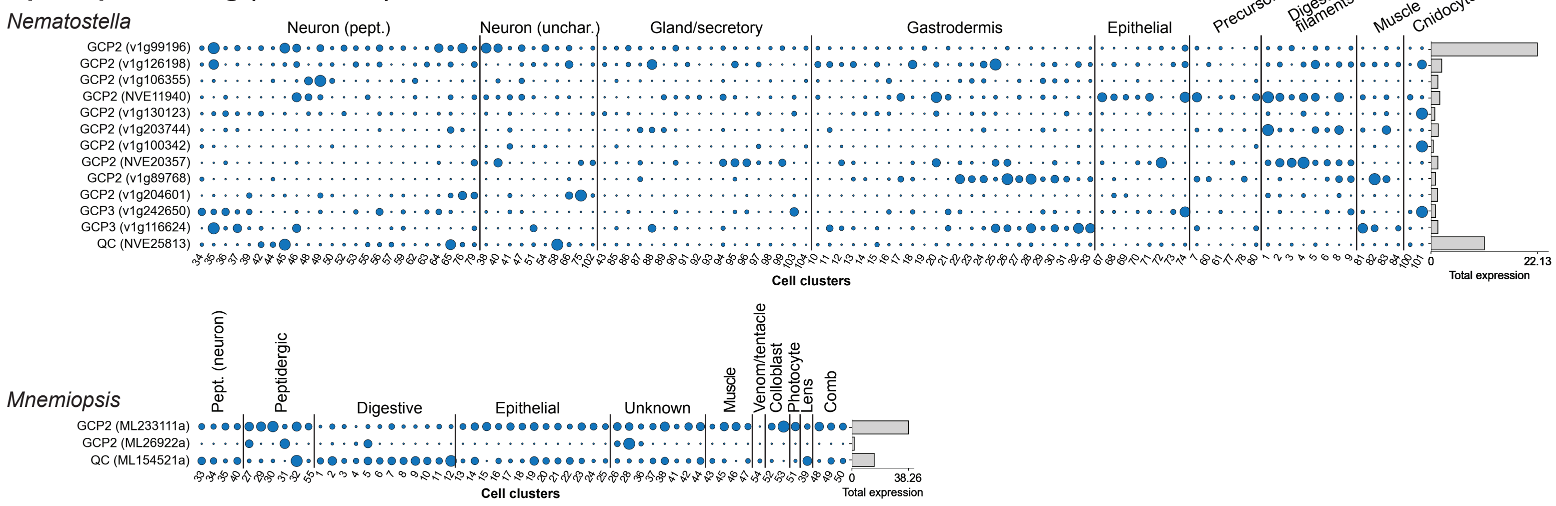

Peptide processing (PAM)

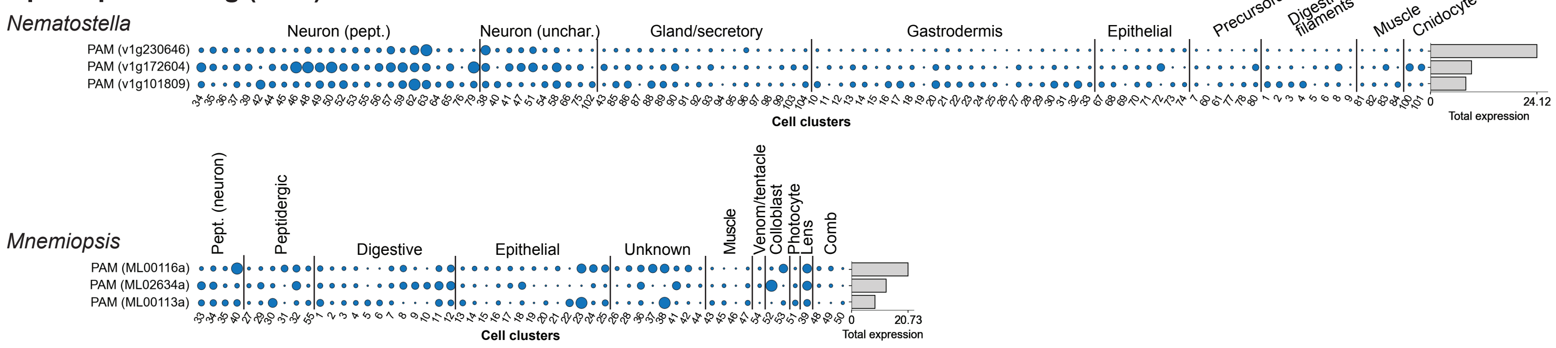

Peptide processing (Neprilysin)

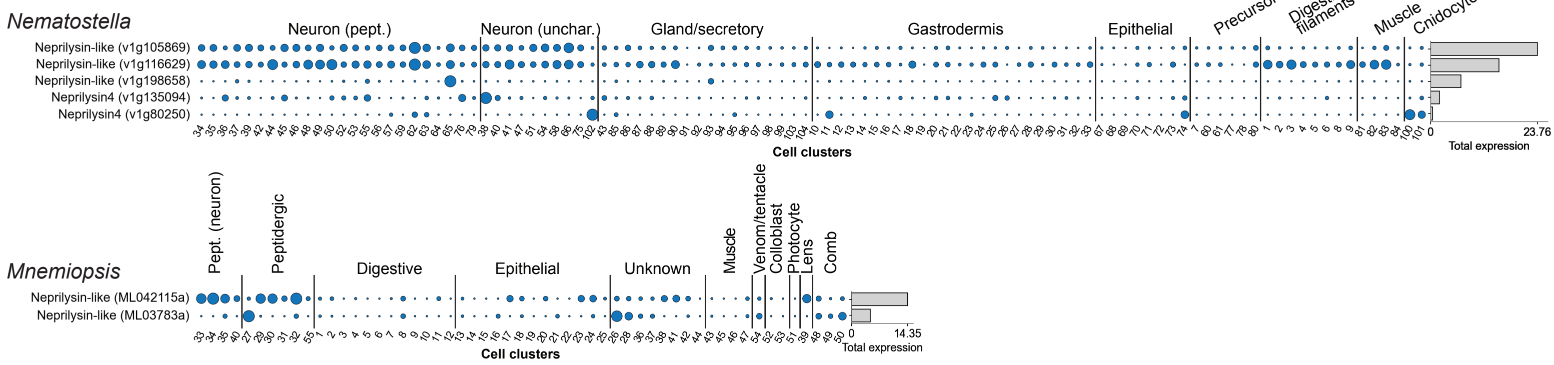

Peptide processing (vATPase)

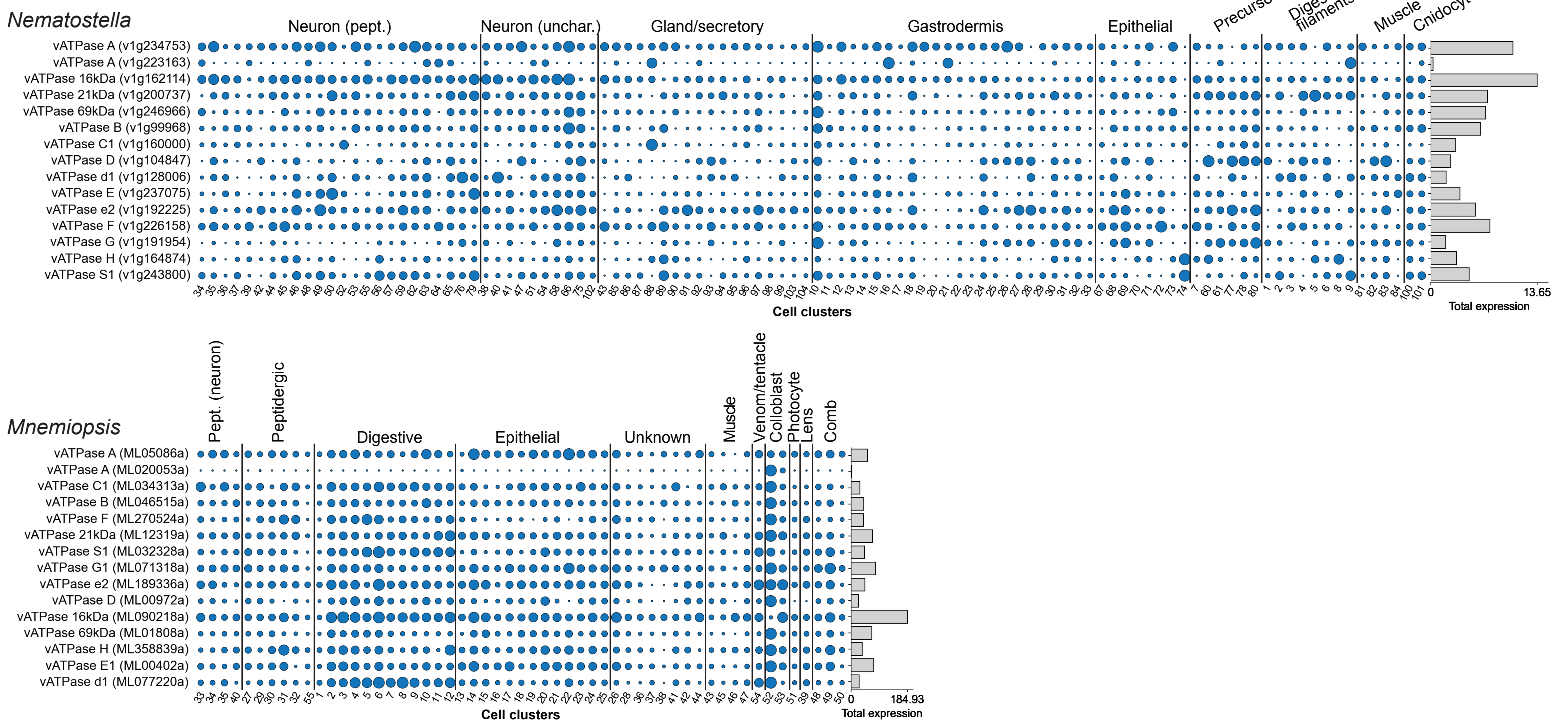

Nociception (ASIC/ENaC-like)

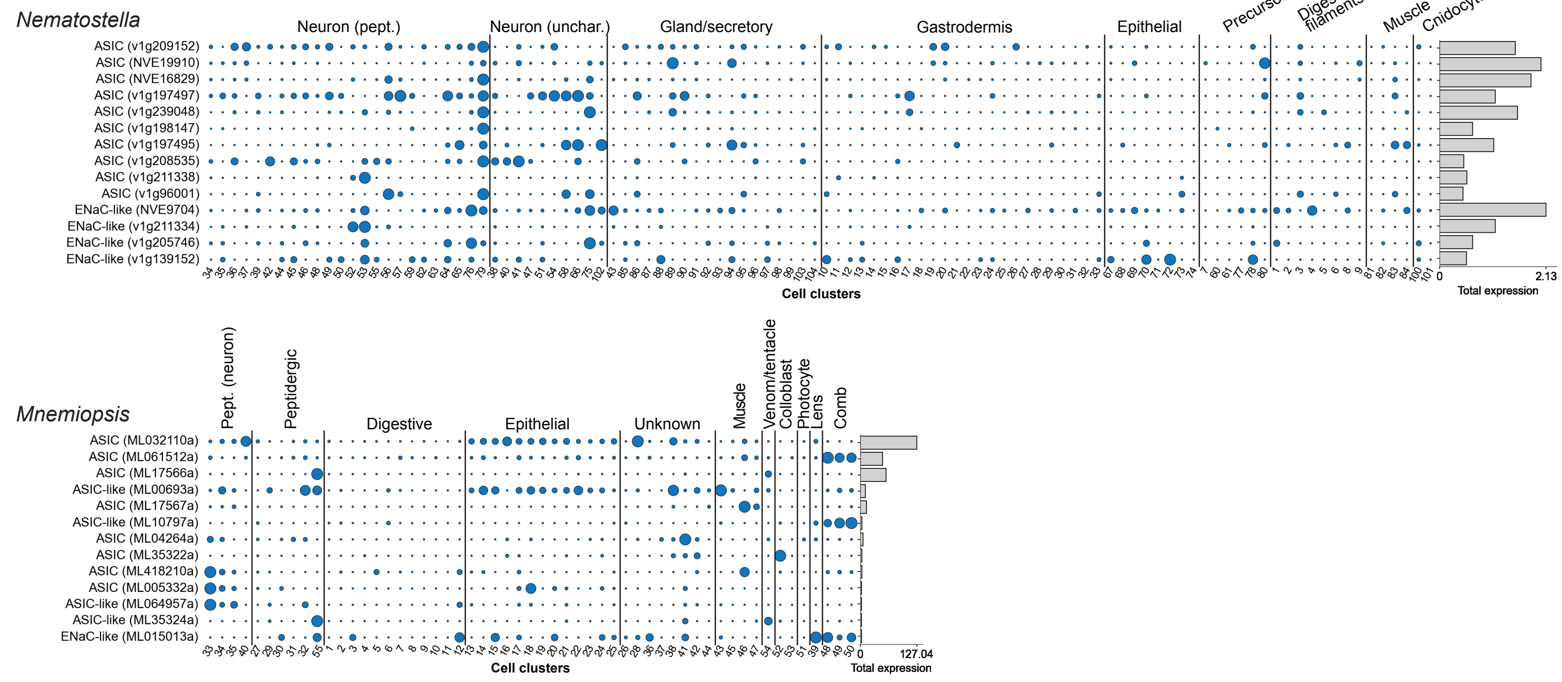

Nociception (TASK/KP2)

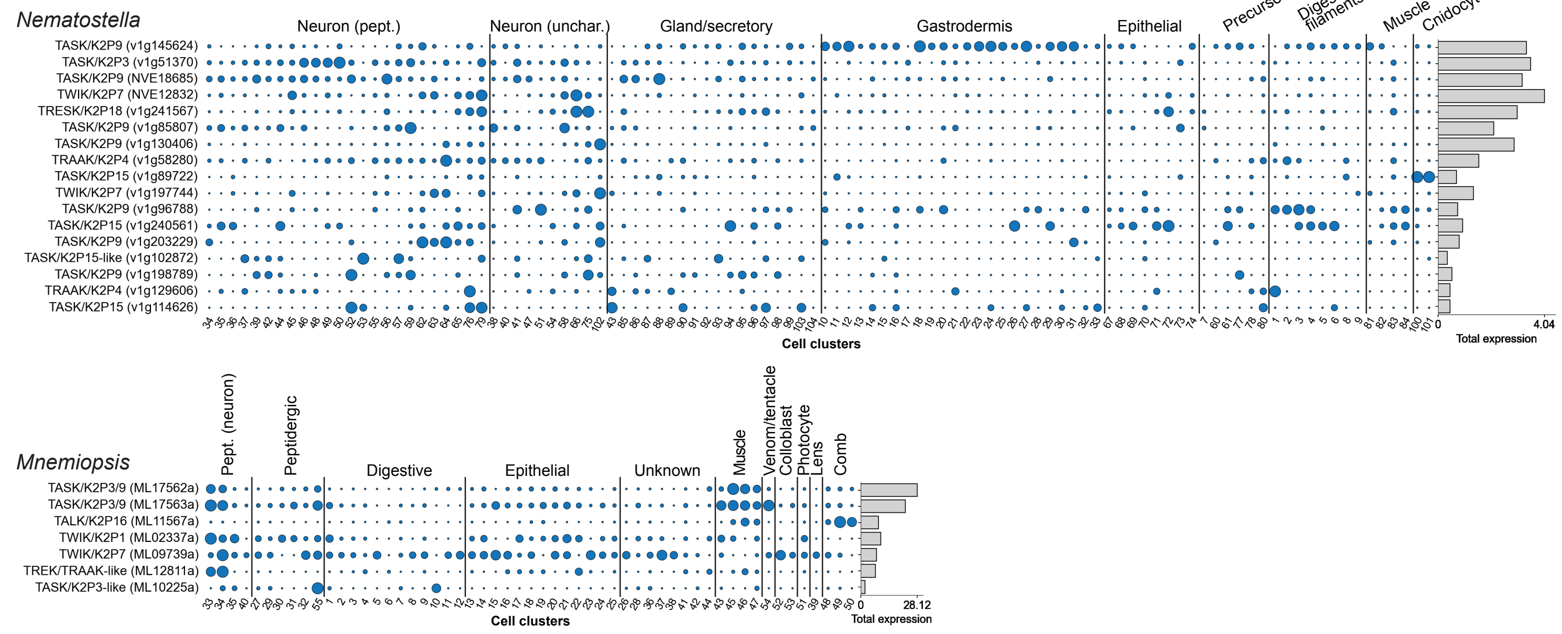

Channel (K(v))

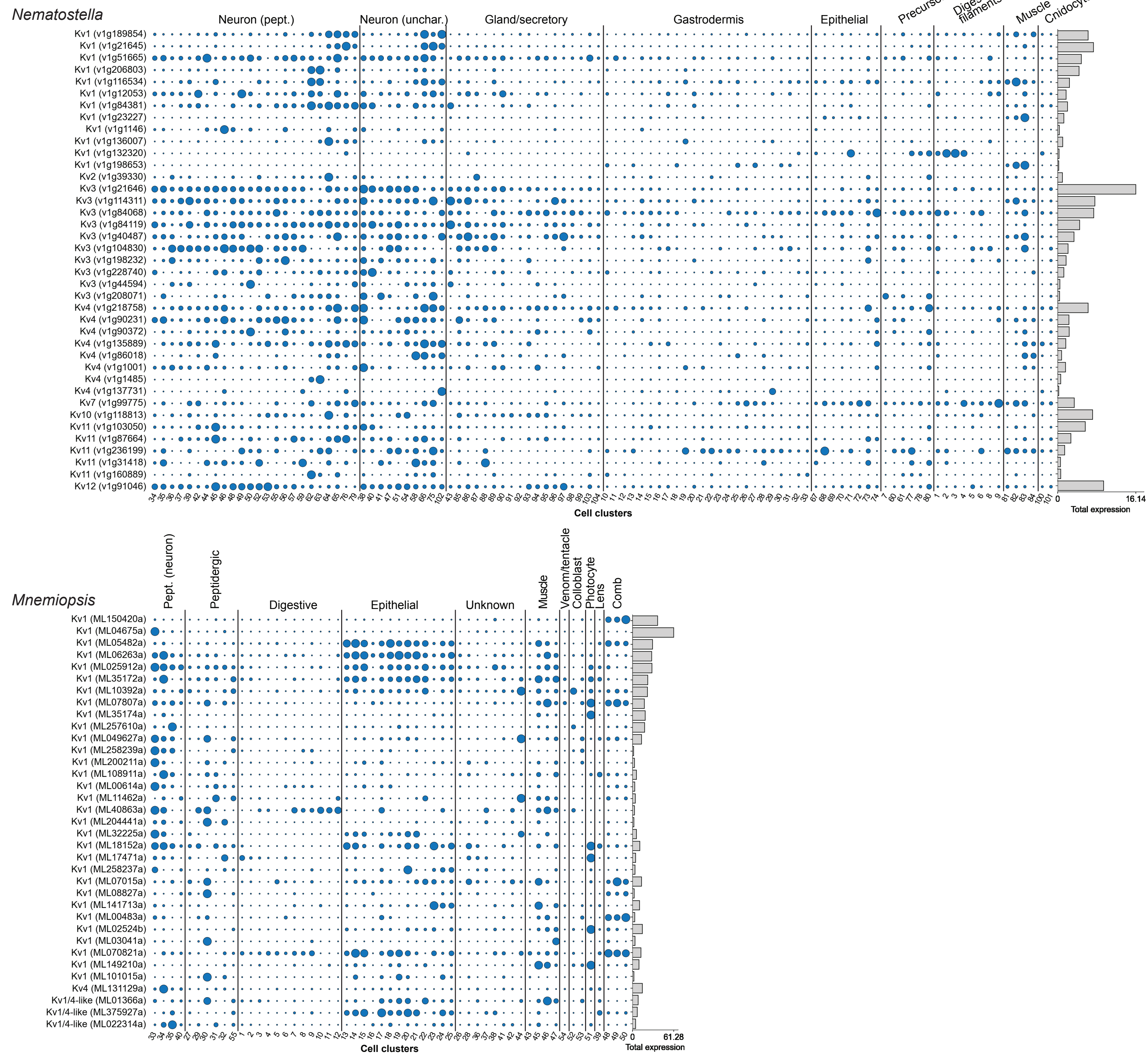

Nematostella
Neuron (pept.)
Neuron (unchar.)
Gland/secretory
Gastrodermis
Epithelial
Precursors
Digestive filaments
Muscle
Cnidocyte
Kv1 (v1g189854)
Kv1 (v1g21645)
Kv1 (v1g51665)
Kv1 (v1g206803)
Kv1 (v1g116534)
Kv1 (v1g12053)
Kv1 (v1g84381)
Kv1 (v1g23227)
Kv1 (v1g1146)
Kv1 (v1g136007)
Kv1 (v1g132320)
Kv1 (v1g198653)
Kv2 (v1g39330)
Kv3 (v1g21646)
Kv3 (v1g114311)
Kv3 (v1g84068)
Kv3 (v1g84119)
Kv3 (v1g40487)
Kv3 (v1g104830)
Kv3 (v1g198232)
Kv3 (v1g228740)
Kv3 (v1g44594)
Kv3 (v1g208071)
Kv4 (v1g218758)
Kv4 (v1g90231)
Kv4 (v1g90372)
Kv4 (v1g135889)
Kv4 (v1g86018)
Kv4 (v1g1001)
Kv4 (v1g1485)
Kv4 (v1g137731)
Kv7 (v1g99775)
Kv10 (v1g118813)
Kv11 (v1g103050)
Kv11 (v1g87664)
Kv11 (v1g236199)
Kv11 (v1g31418)
Kv11 (v1g160889)
Kv12 (v1g91046)
Cell clusters
0
16.14
Total expression
Mnemiopsis
Pept. (neuron)
Peptidergic
Digestive
Epithelial
Unknown
Muscle
Venom/tentacle
Colloblast
Photocyte
Lens
Comb
Kv1 (ML150420a)
Kv1 (ML04675a)
Kv1 (ML05482a)
Kv1 (ML06263a)
Kv1 (ML025912a)
Kv1 (ML35172a)
Kv1 (ML10392a)
Kv1 (ML07807a)
Kv1 (ML35174a)
Kv1 (ML257610a)
Kv1 (ML049627a)
Kv1 (ML258239a)
Kv1 (ML200211a)
Kv1 (ML108911a)
Kv1 (ML00614a)
Kv1 (ML11462a)
Kv1 (ML40863a)
Kv1 (ML204441a)
Kv1 (ML32225a)
Kv1 (ML18152a)
Kv1 (ML17471a)
Kv1 (ML258237a)
Kv1 (ML07015a)
Kv1 (ML08827a)
Kv1 (ML141713a)
Kv1 (ML00483a)
Kv1 (ML02524b)
Kv1 (ML03041a)
Kv1 (ML070821a)
Kv1 (ML149210a)
Kv1 (ML101015a)
Kv4 (ML131129a)
Kv1/4-like (ML01366a)
Kv1/4-like (ML375927a)
Kv1/4-like (ML022314a)
Cell clusters
0
61.28
Total expression

Channel (K(Ca))

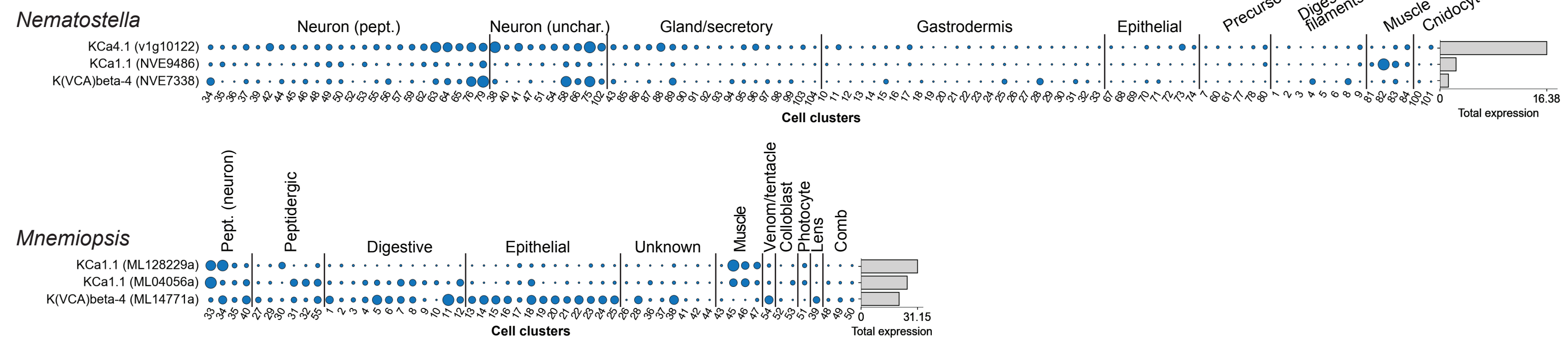

Channel (Ca(v), Na(v))

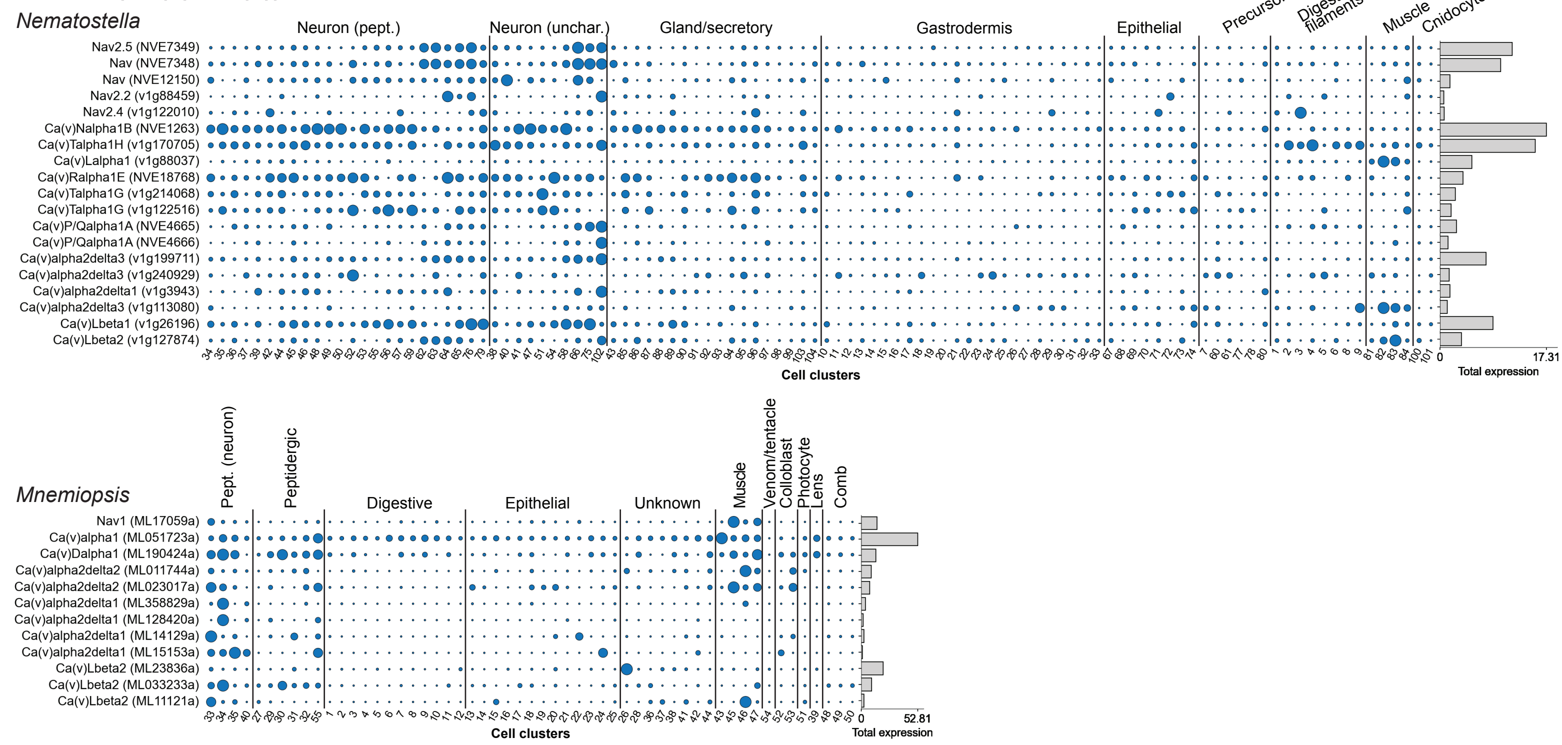

Ca signaling (Calmodulin)

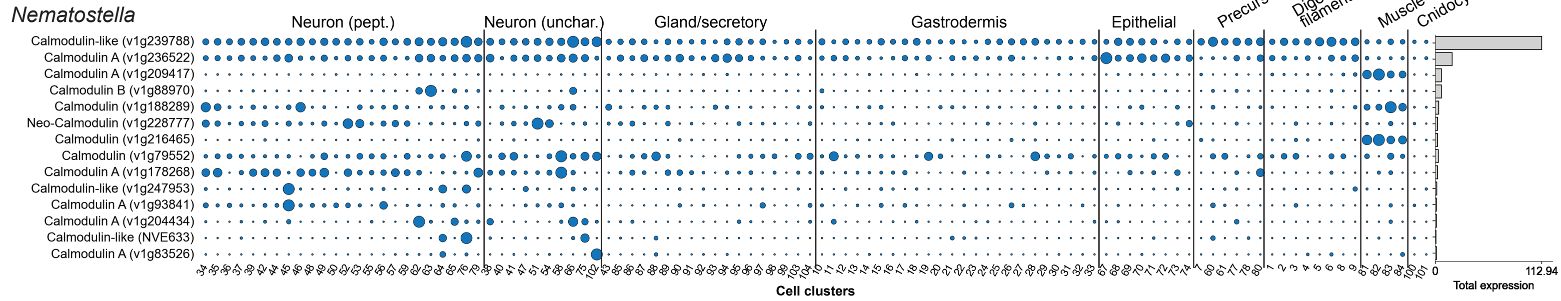

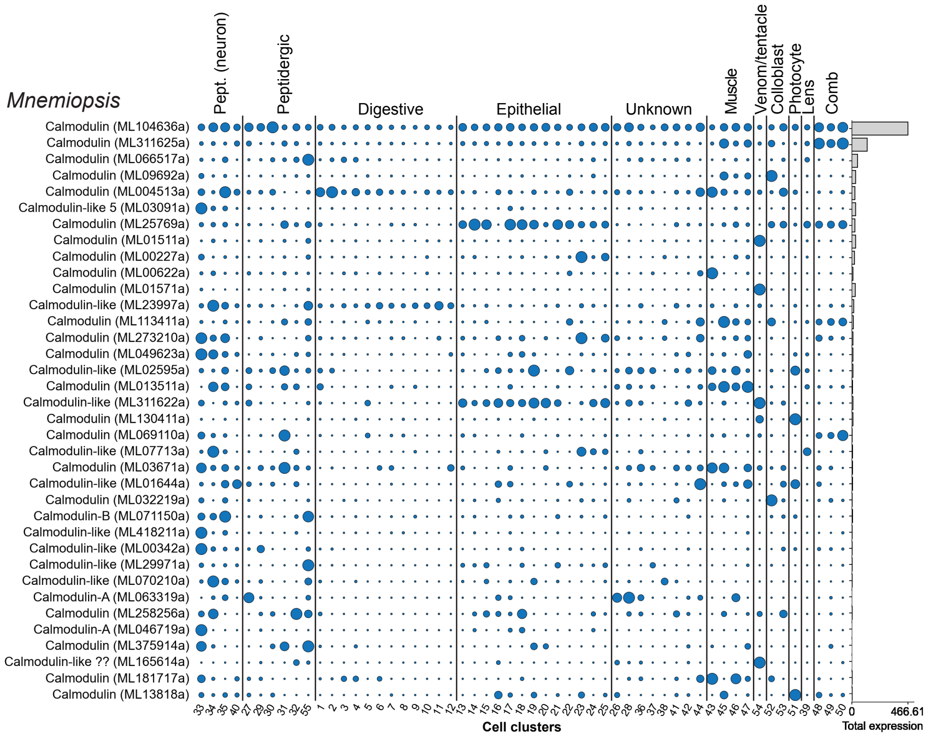

Ca signaling (CaM Kinase, CaM-KK)

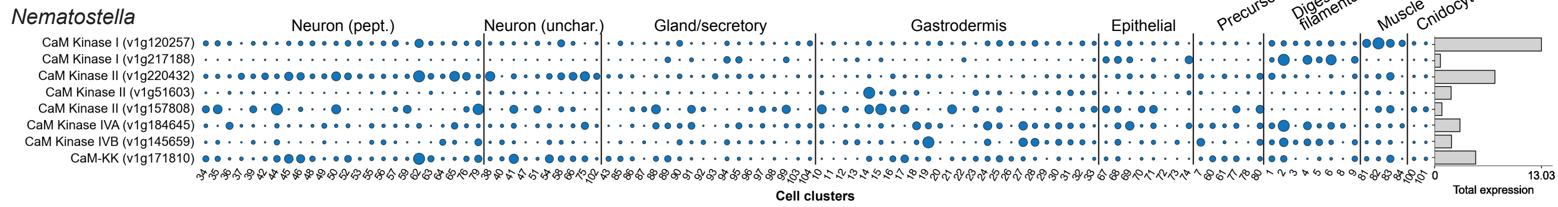

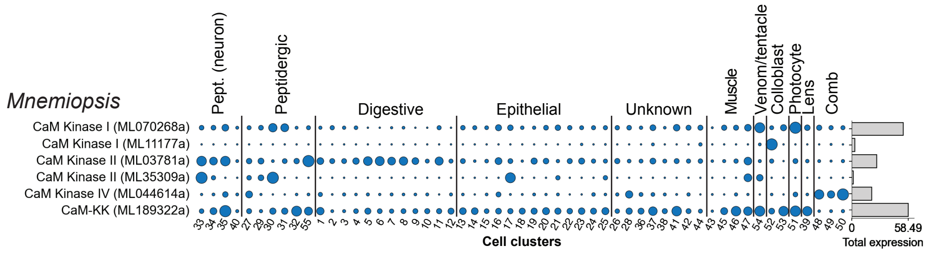

Ca signaling (NCS1)

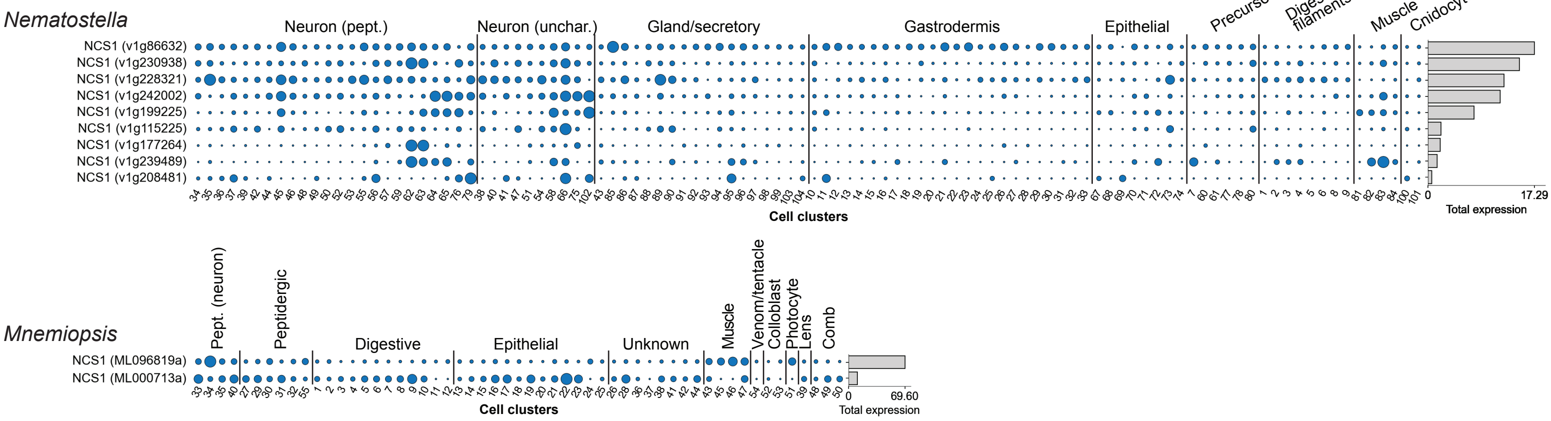

Vesicle transport (Rab)

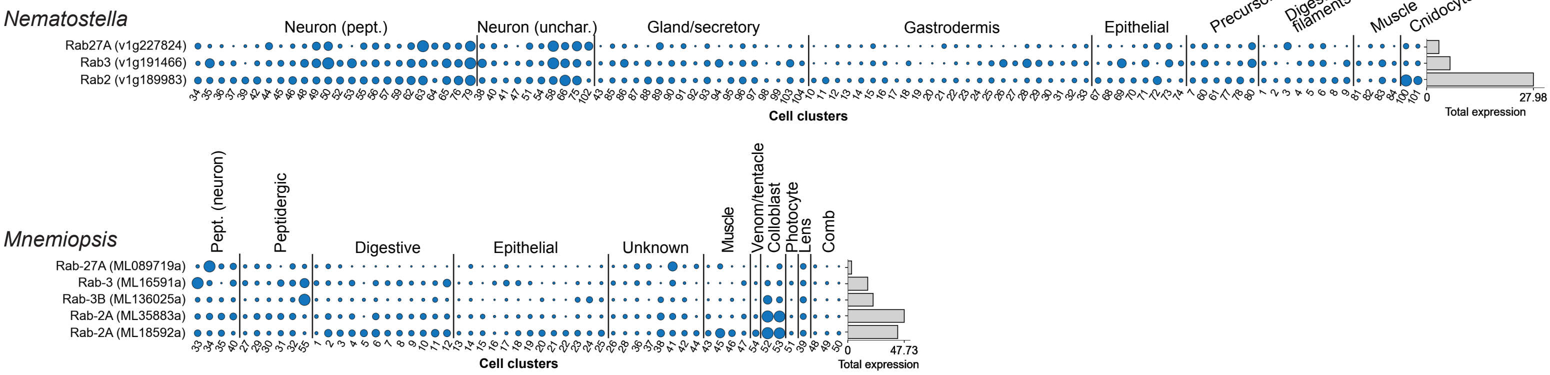

Vesicle transport (Kinesin)

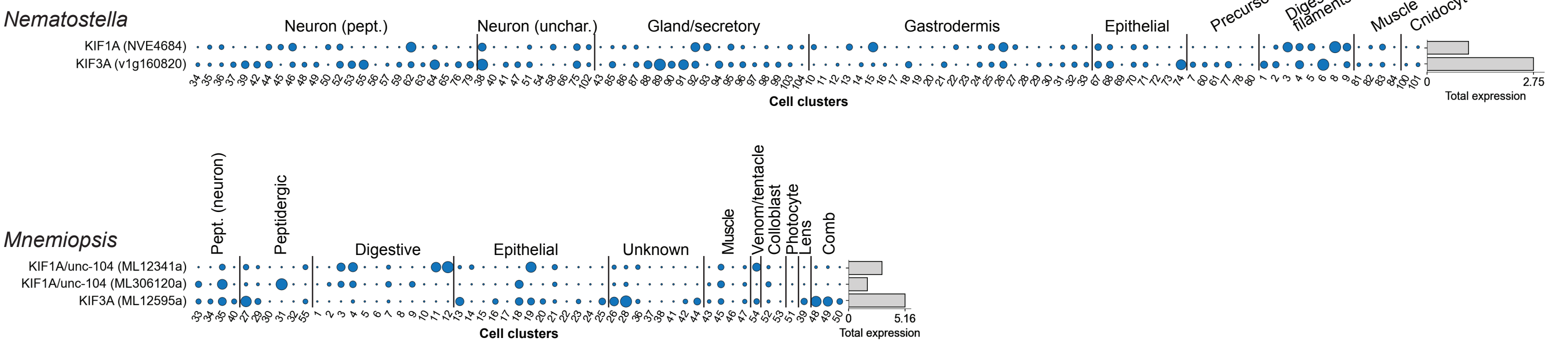

Vesicle transport (Dynactin, JIP, Huntingtin, TRAK2/HAP1, Reticulon)

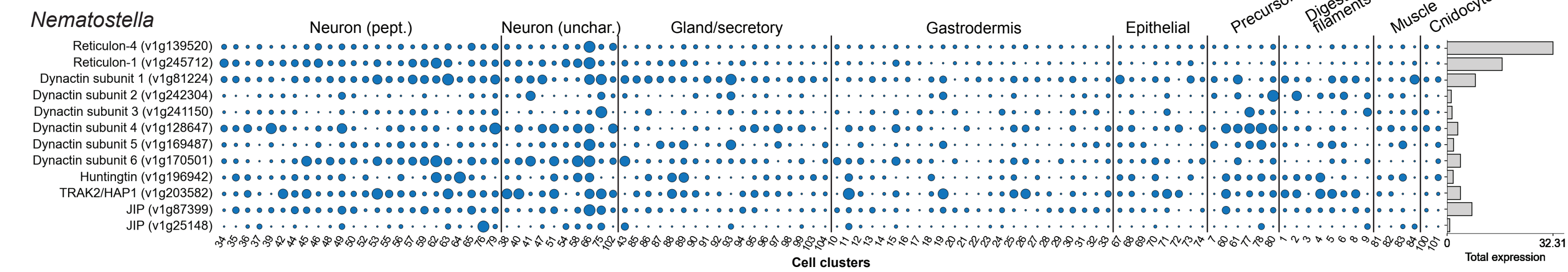

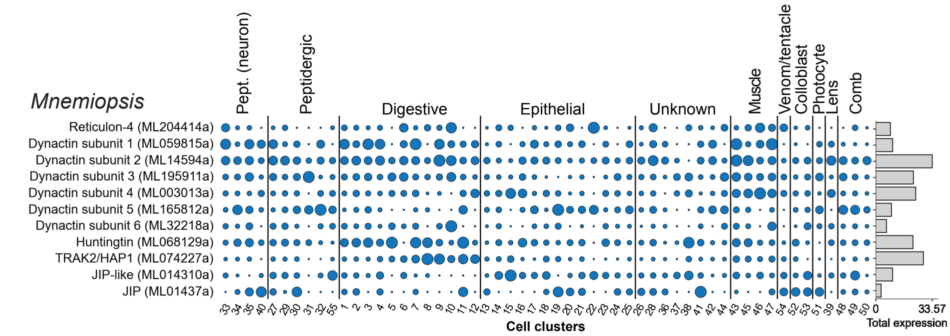

Vesicle transport (Sec, Phogrin, AP180)

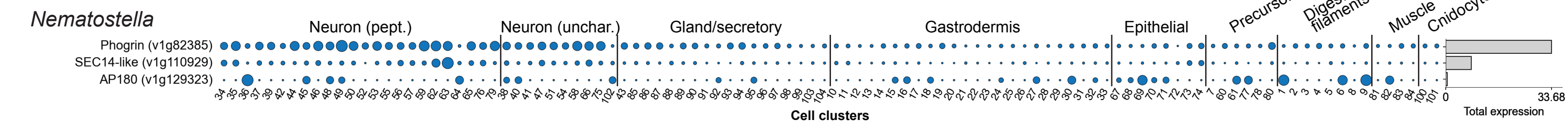

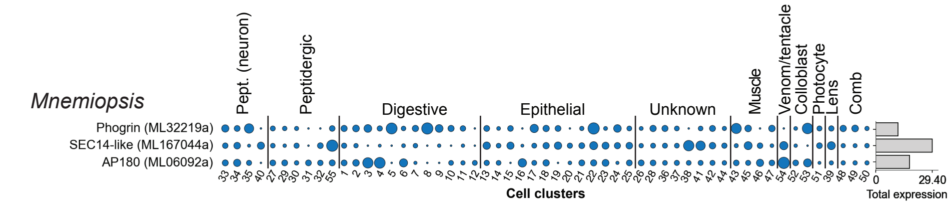

Vesicle release (Rabphilin, MCTP2, NOC)

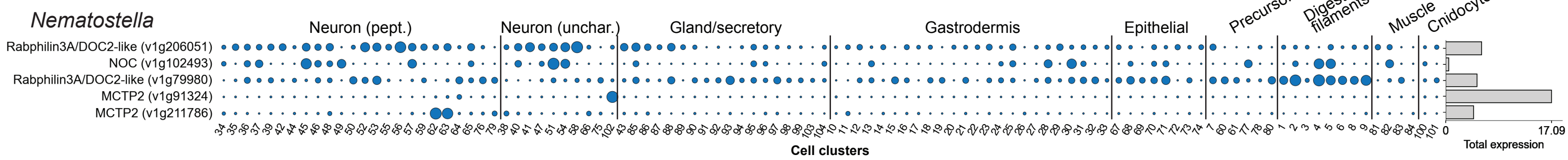

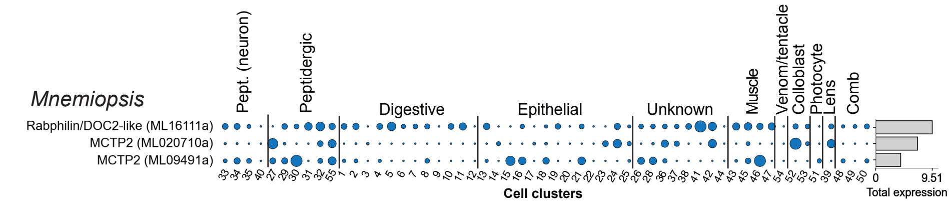

Vesicle release (Synaptotagmin)

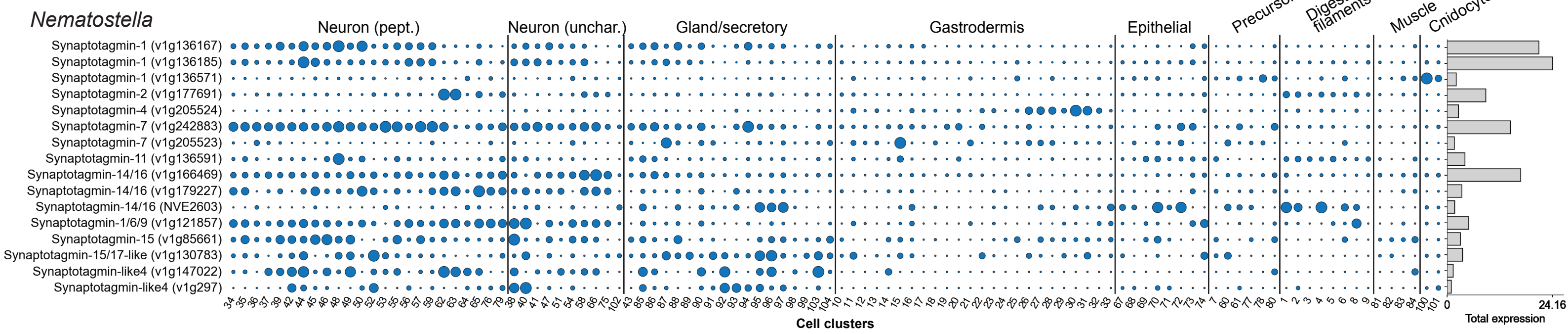

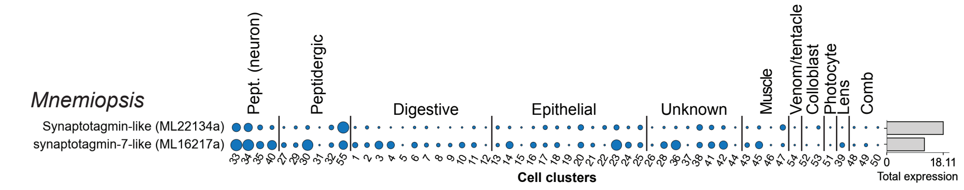

Vesicle release (Syntaxin, VAMP, SNAP25, YKT6, Synapsin)

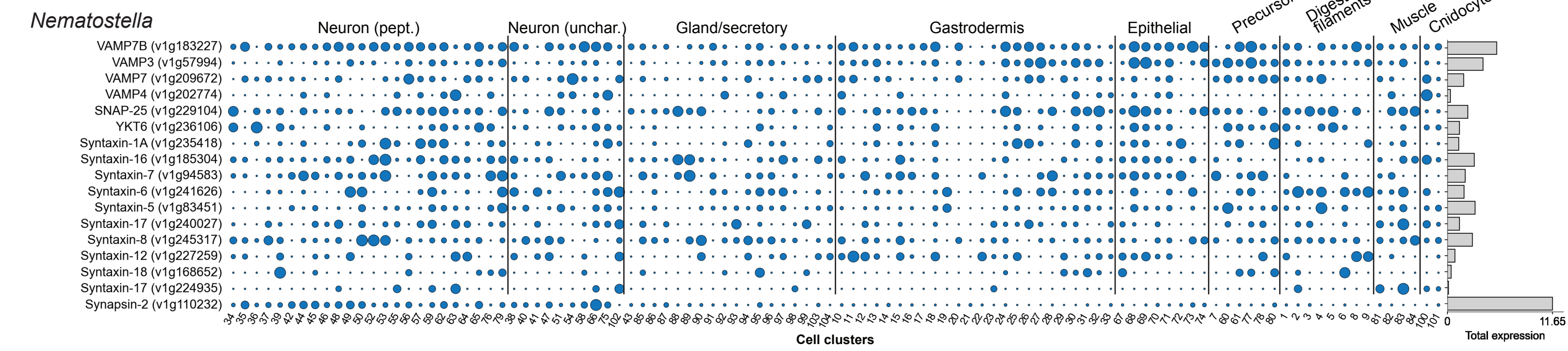

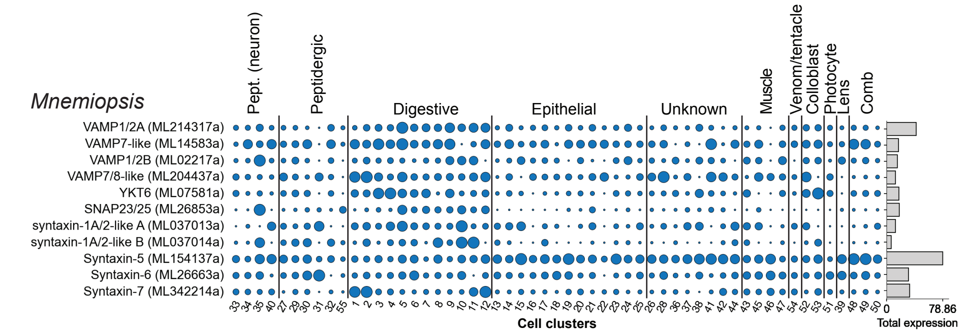

Vesicle release, Tethering (Munc13, STXBP, Sly1p, CAPS1, BAIAP3, RIMS, RIMBP, Liprin-alpha, CASK, Veli, HID1, Tomosyn, Secretagogin, Calbindin-32, TANC1)

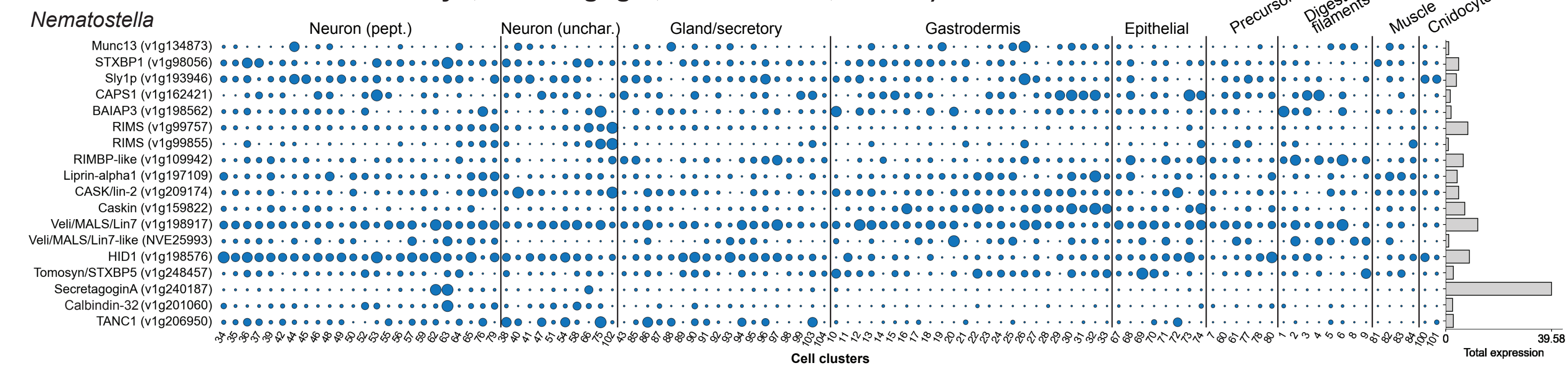

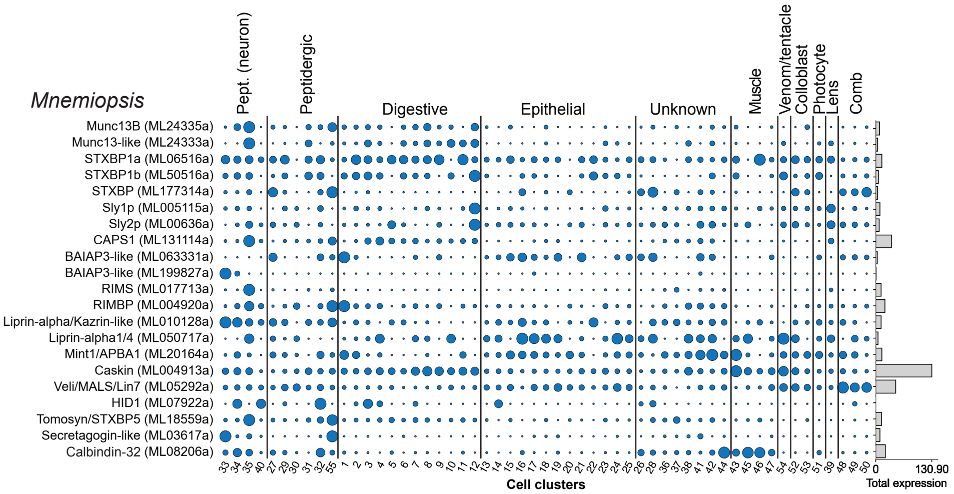

Synapse (Neurexin, PSD95, MAGUK)

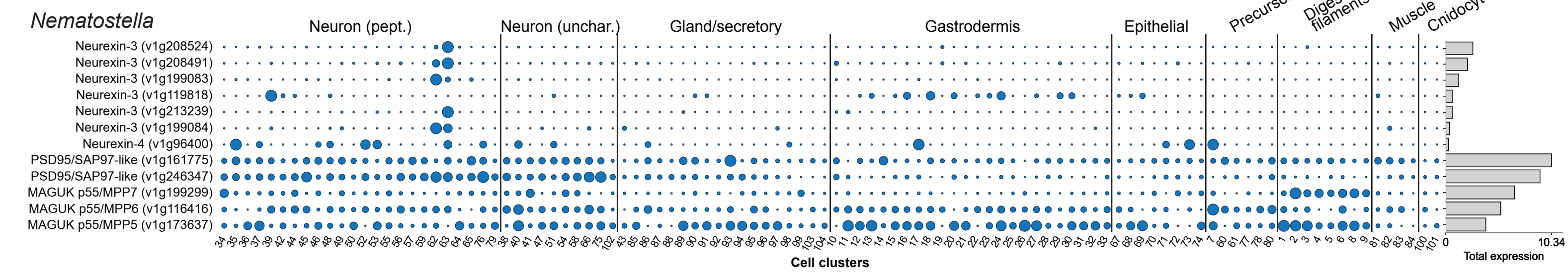

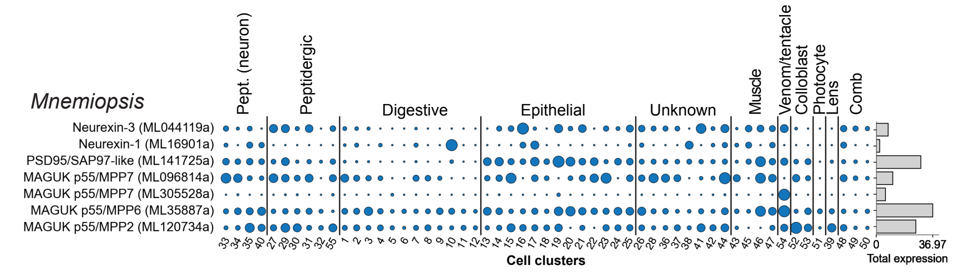

Endocytic recyling (Synaptojanin, Inpp5, SAC, OCRL, PPIP)

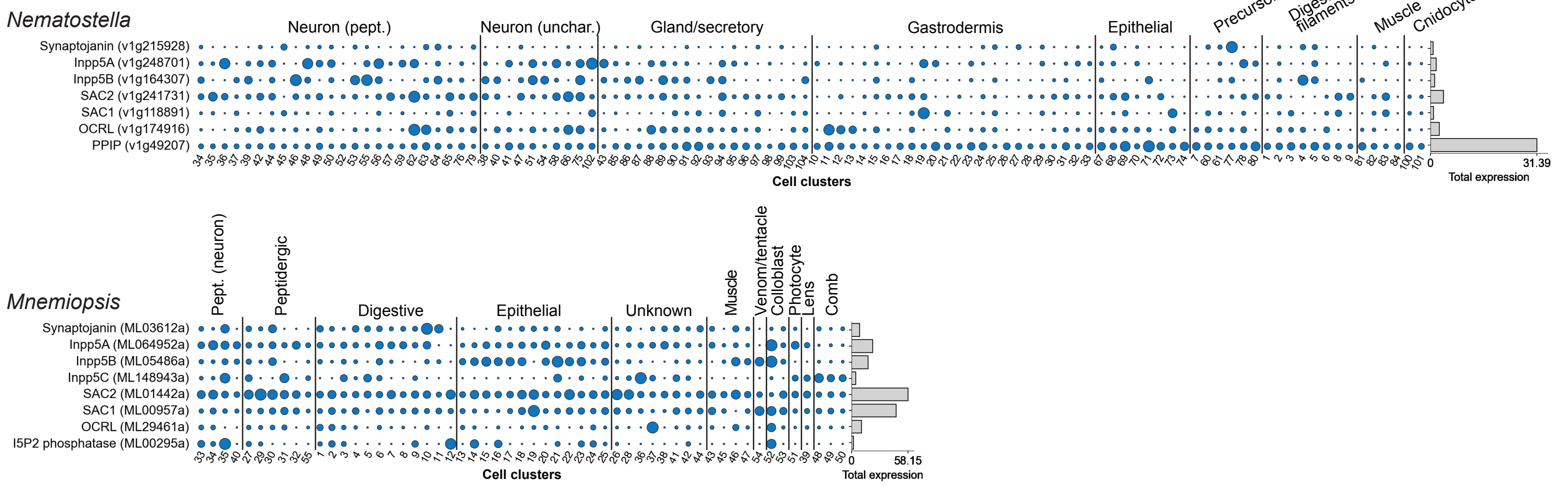

Gene expression (ELAV, Musashi)

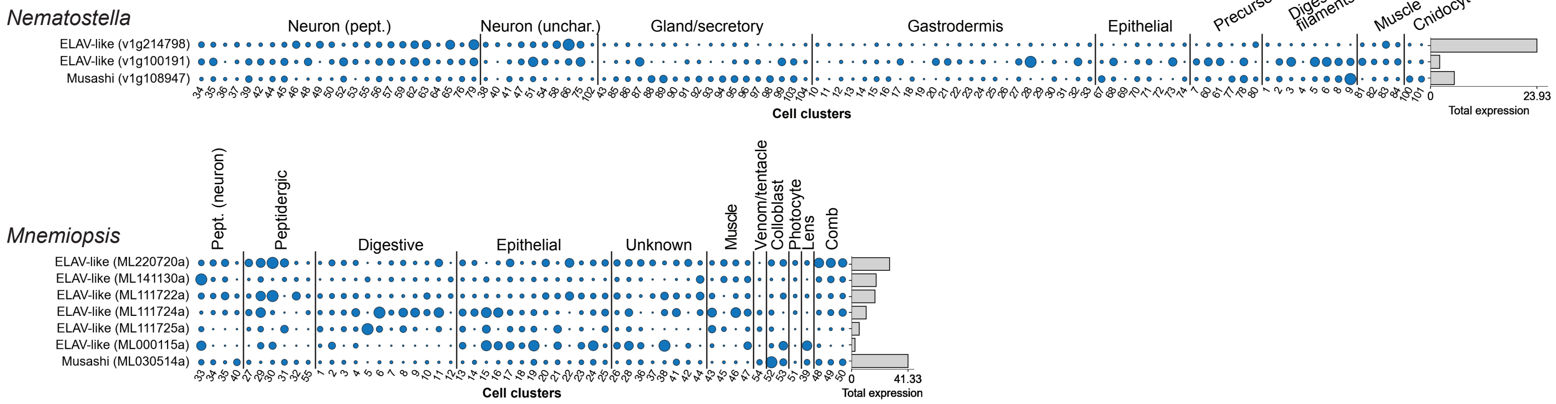

Gene expression (Sox)

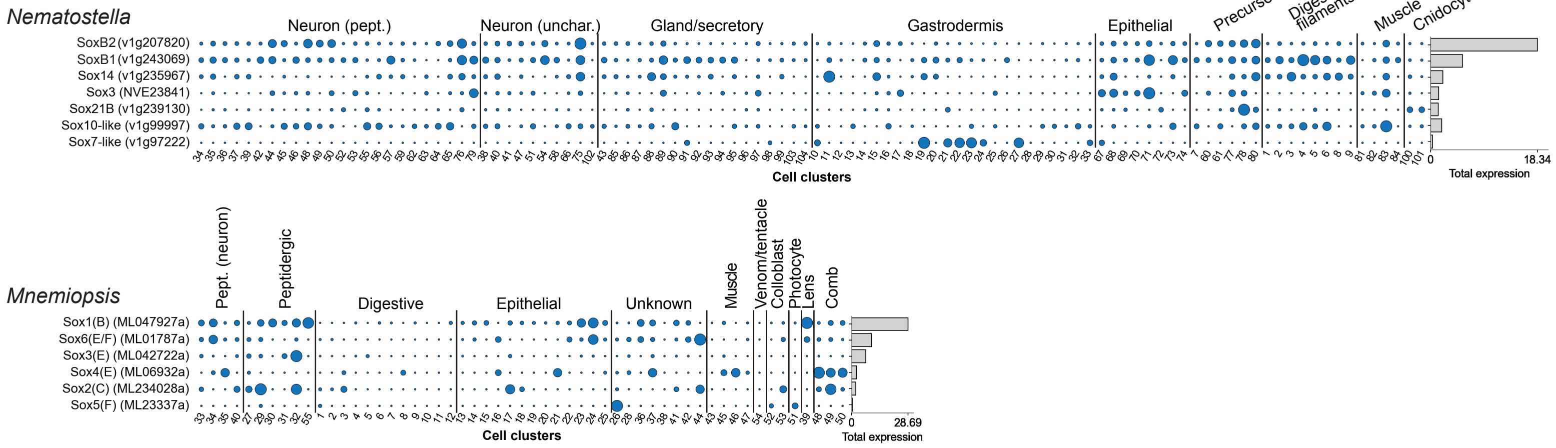

Gene expression (bHLH)

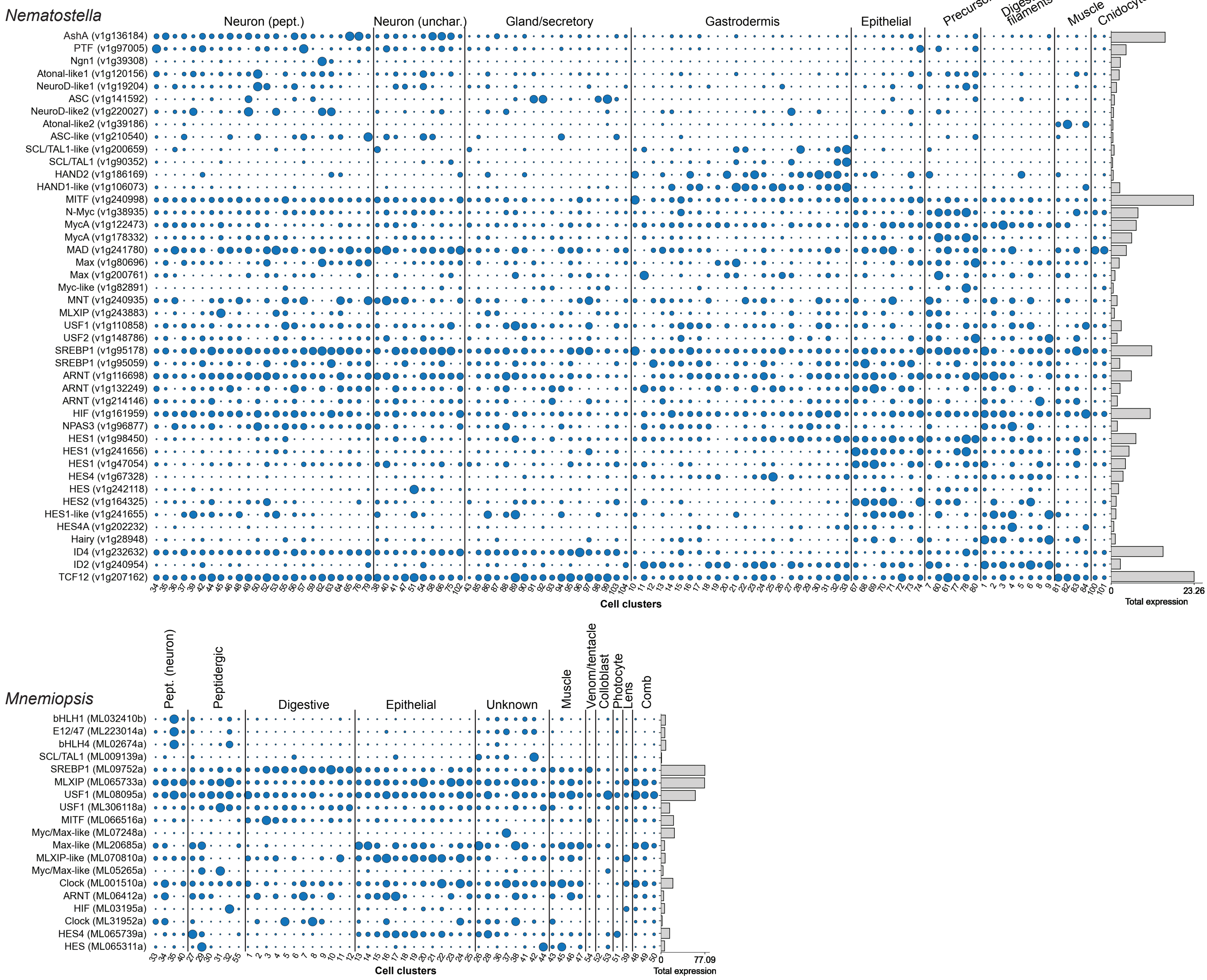

Gene expression (Forkhead box)

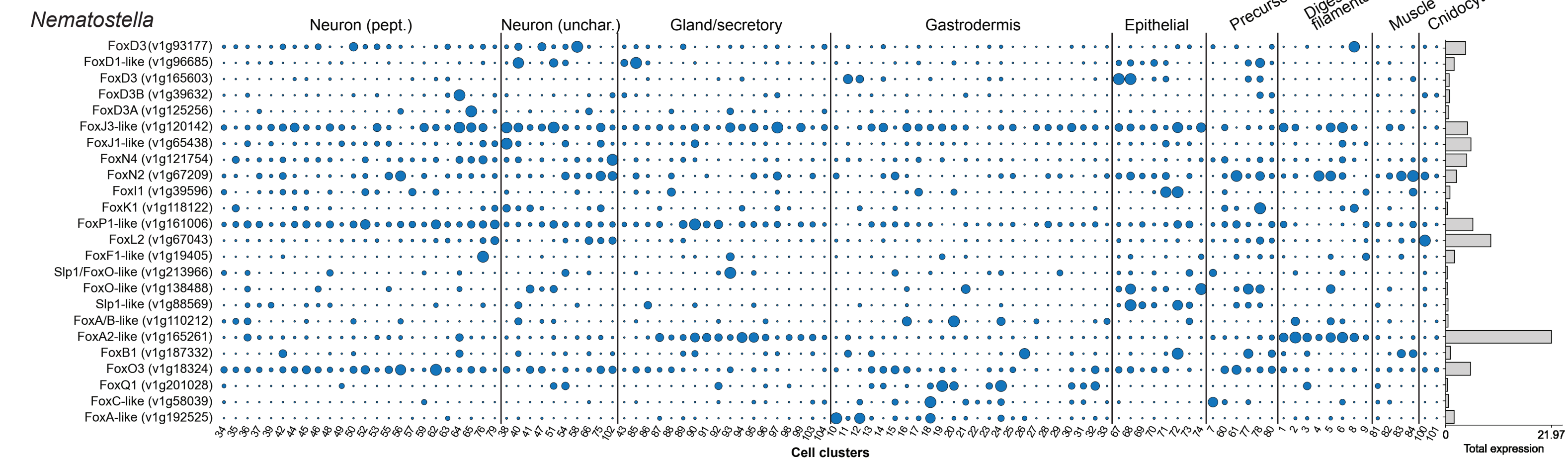

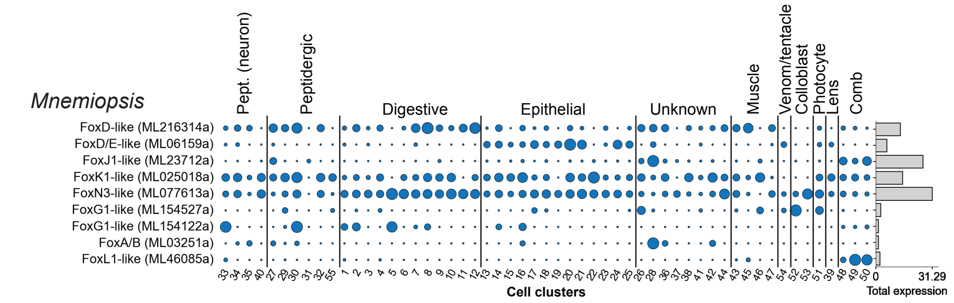

Gene expression (Lim class homeobox)

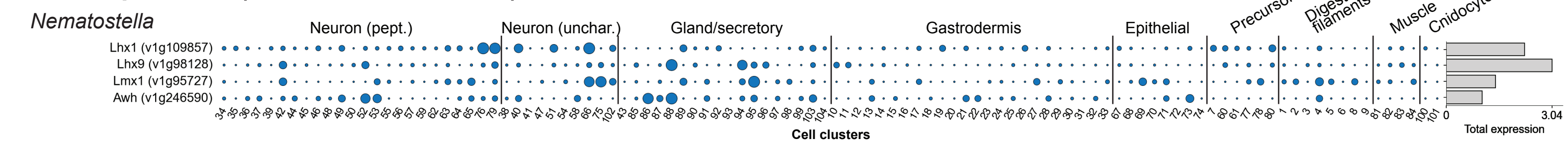

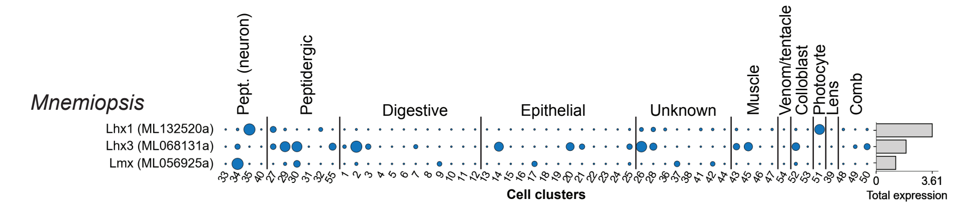

Gene expression (PRD homeobox)

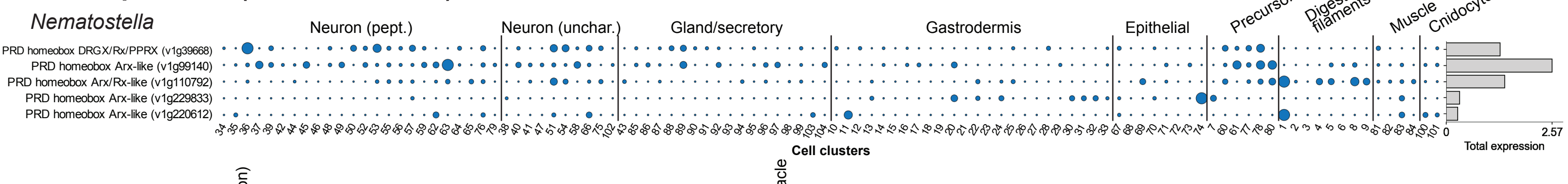

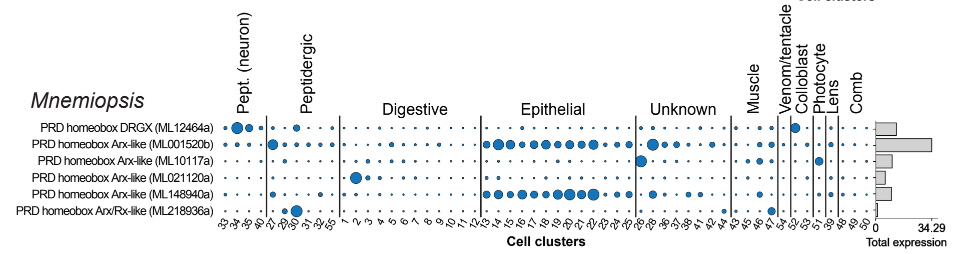

Gene expression (ANTP class homeobox)

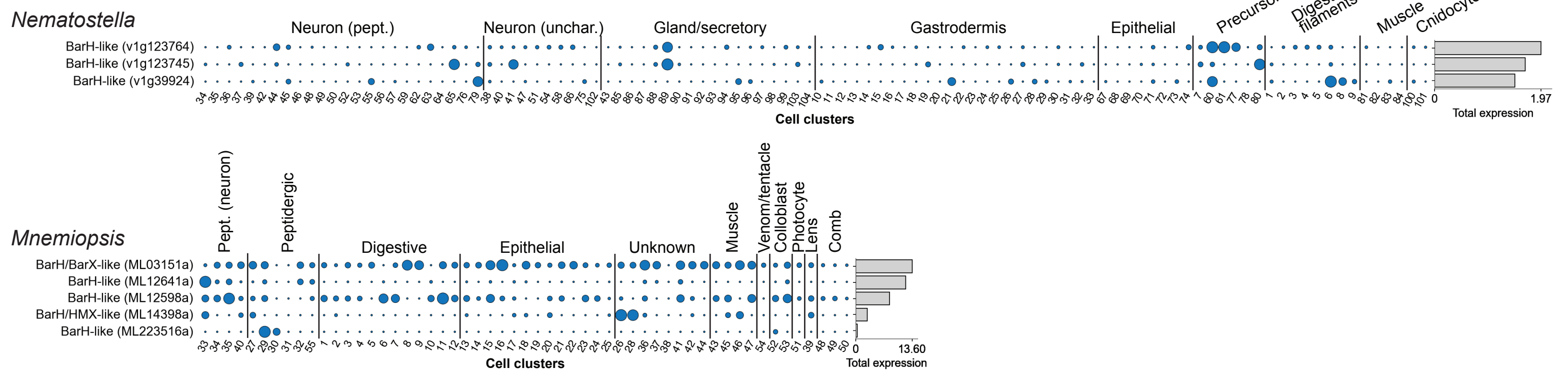

Gene expression (POU class homeobox)

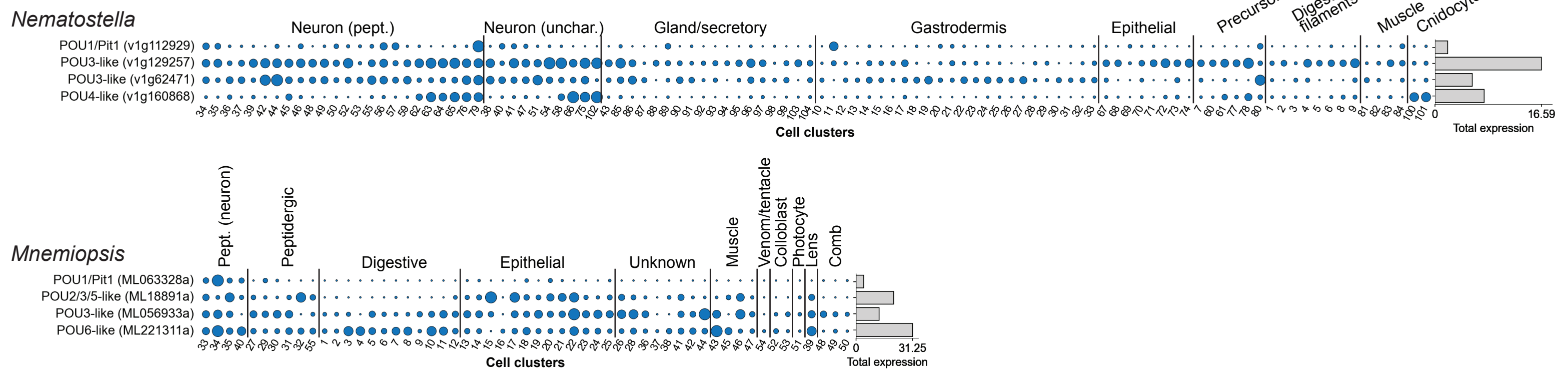

Gene expression (Six class homeobox)

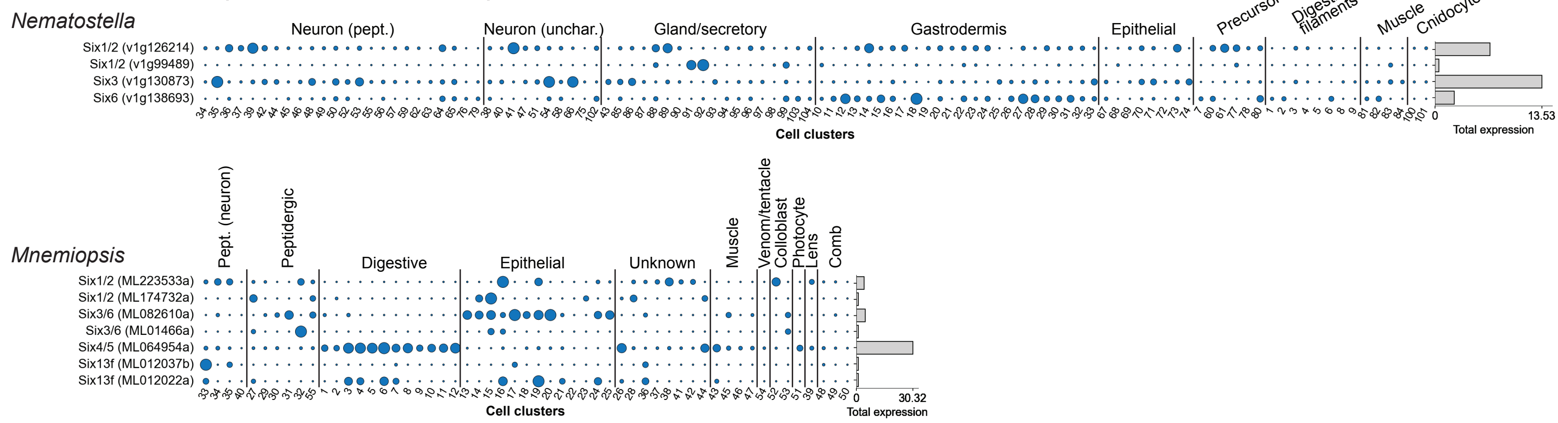

Gene expression (Drmt, CAMTA)

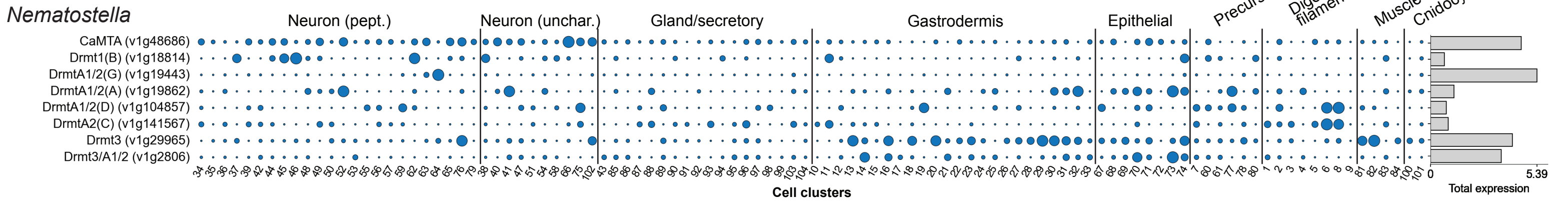

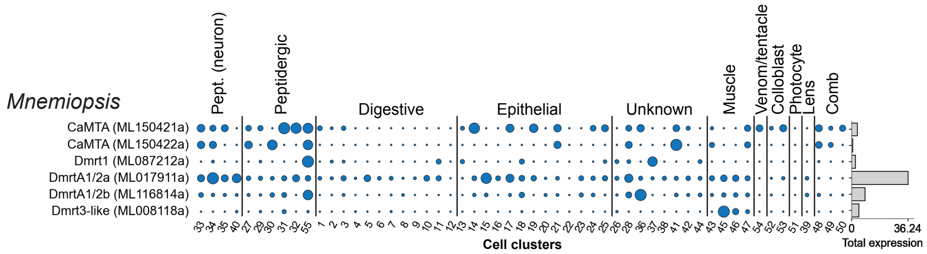

Transporters (vGluT/Sialin)

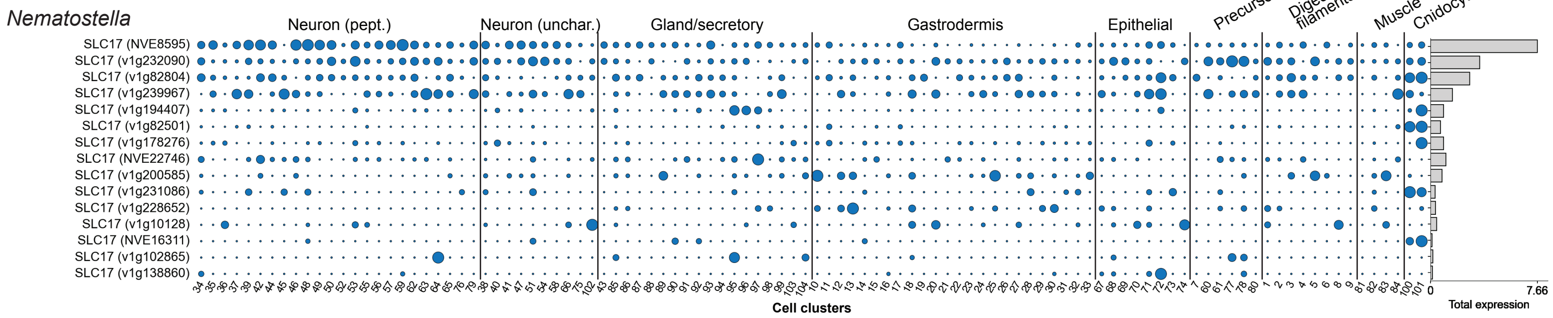

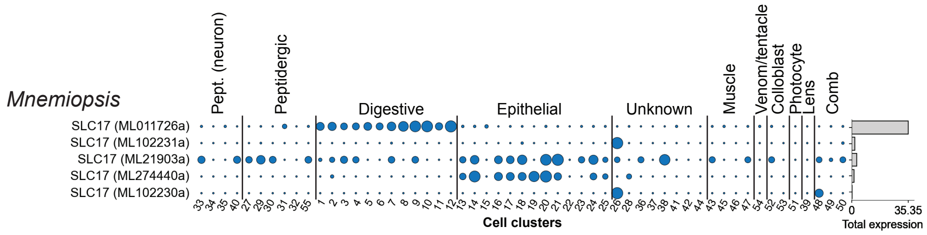

Transporters (vMonoamine/Ach)

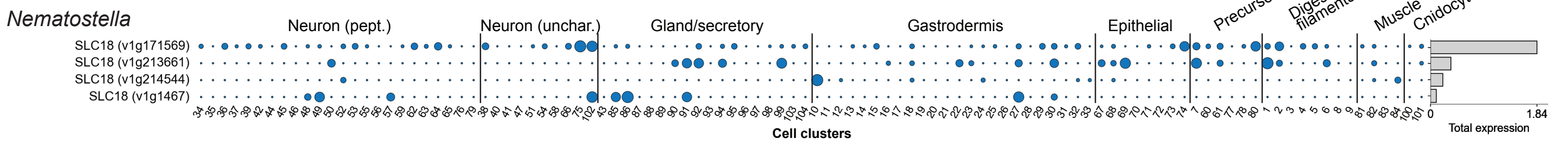

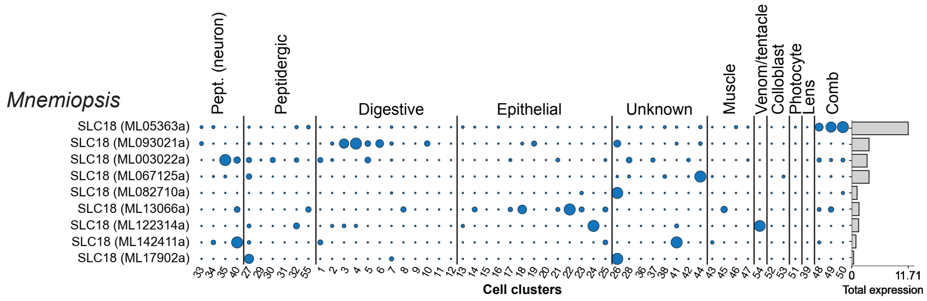

Transporters (vGABA/Gly)

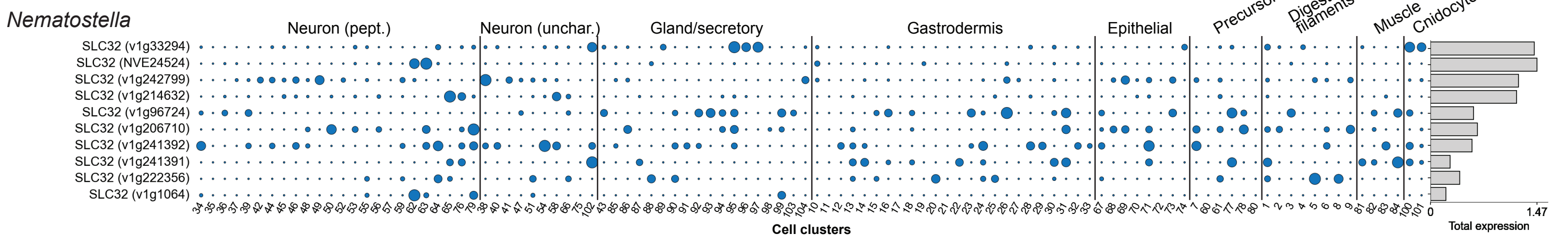

Transporters (EAAT/Glast)

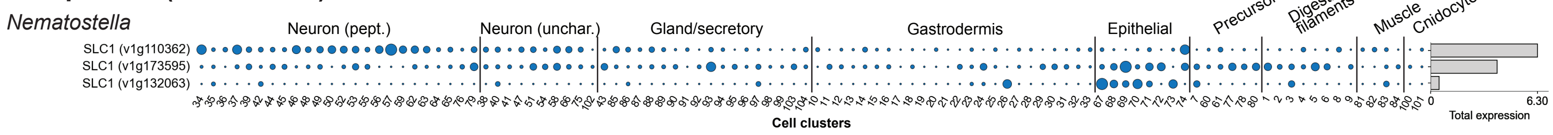

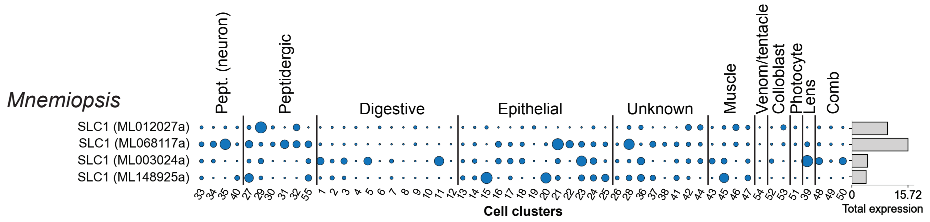

Transporters (GABA/Dopamine/Serotonin/Noradre)

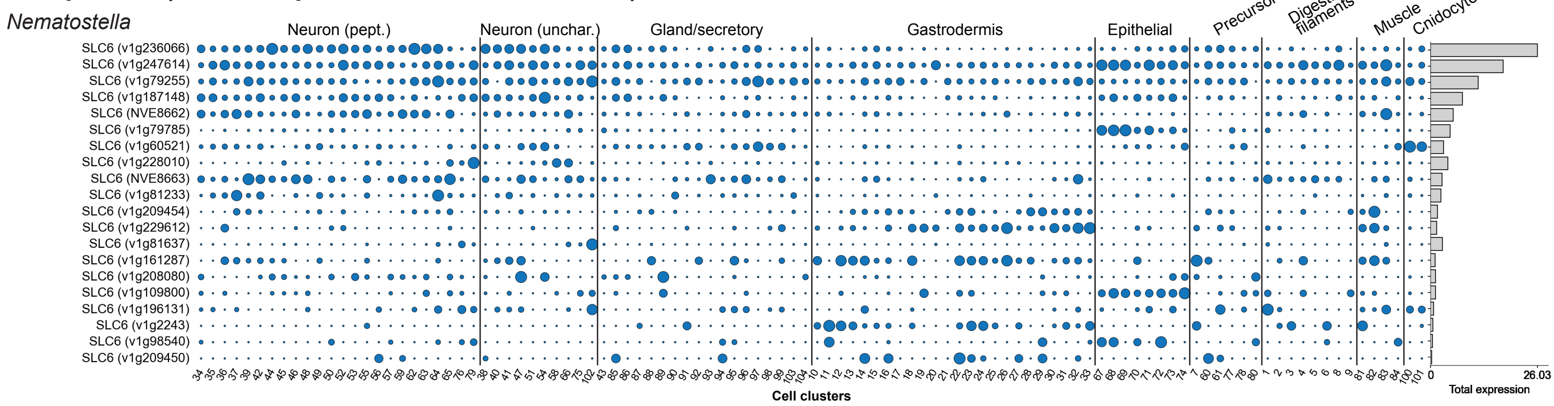

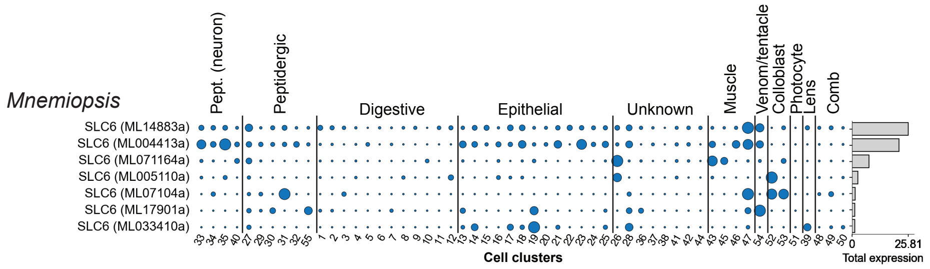

Receptors (Acetylcholine)

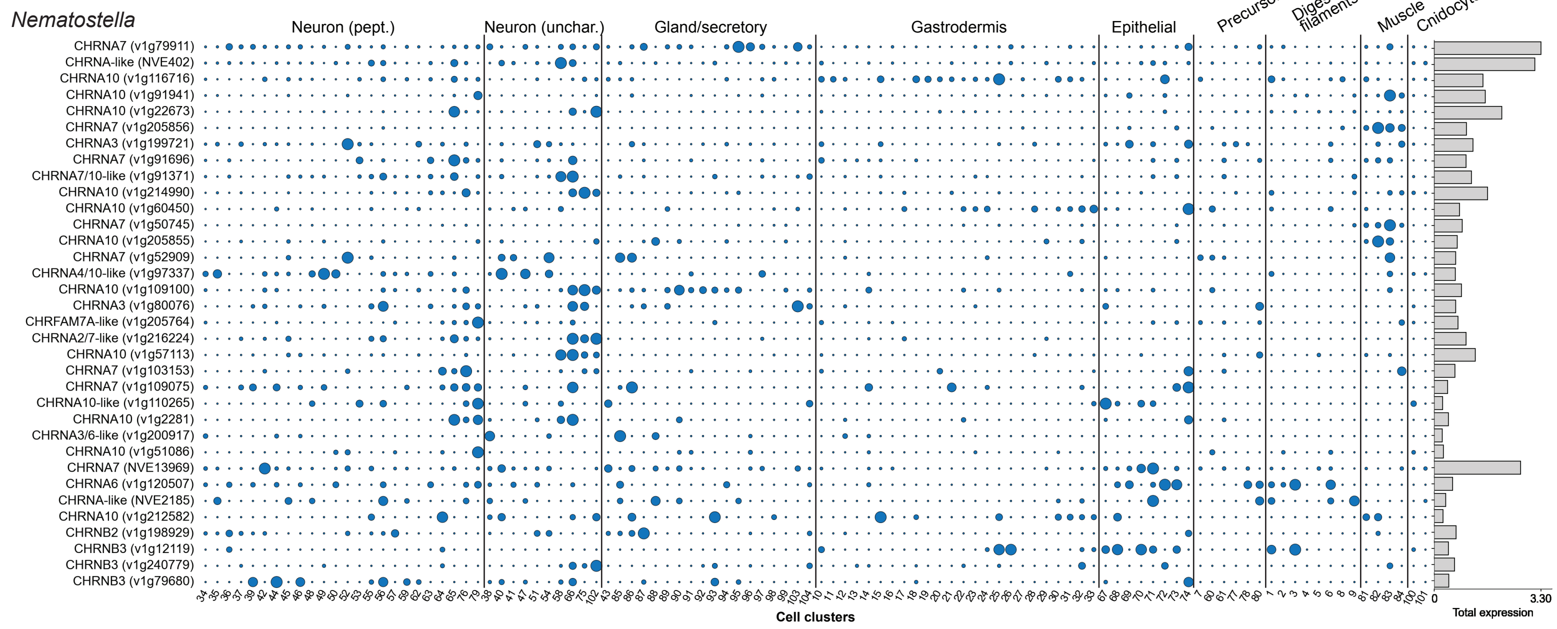

Receptors (iGluR)

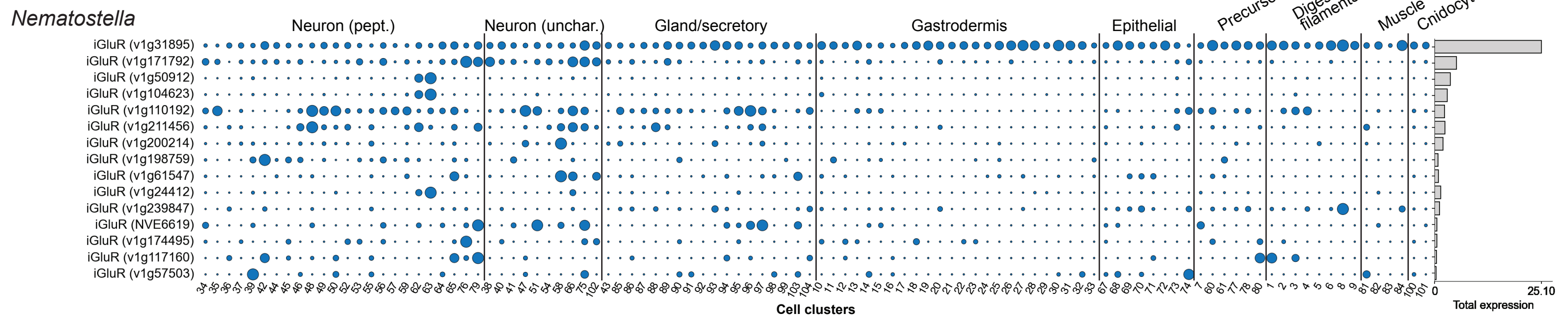

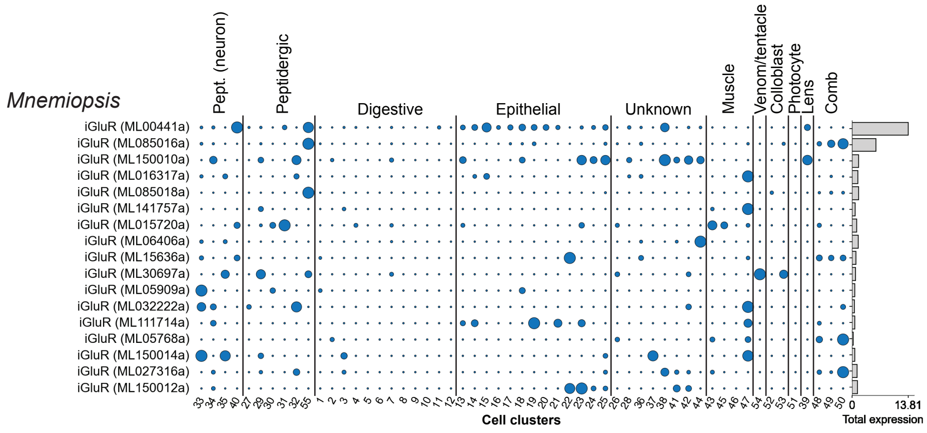

Receptors (mGluR)

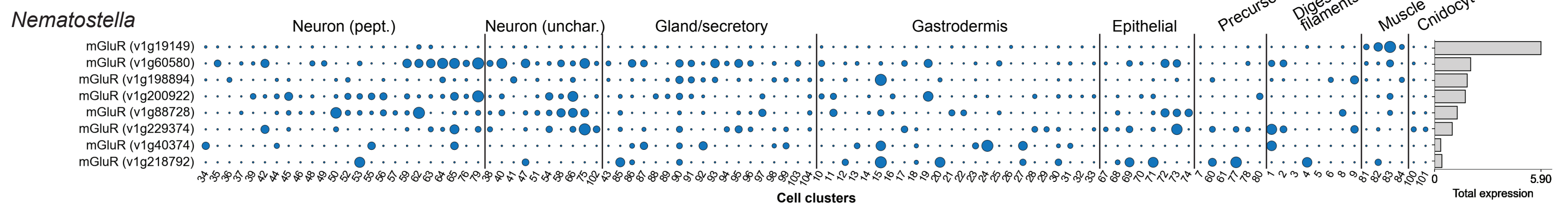

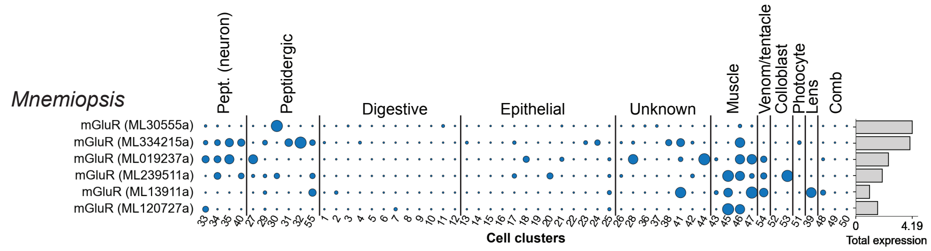

Receptors (Cl channel - Glycine)

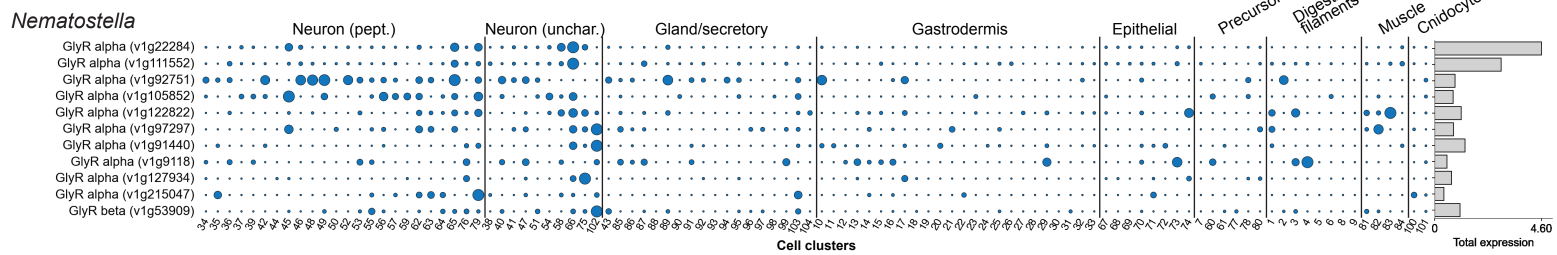

Receptors (Cl channel - GABA(A))

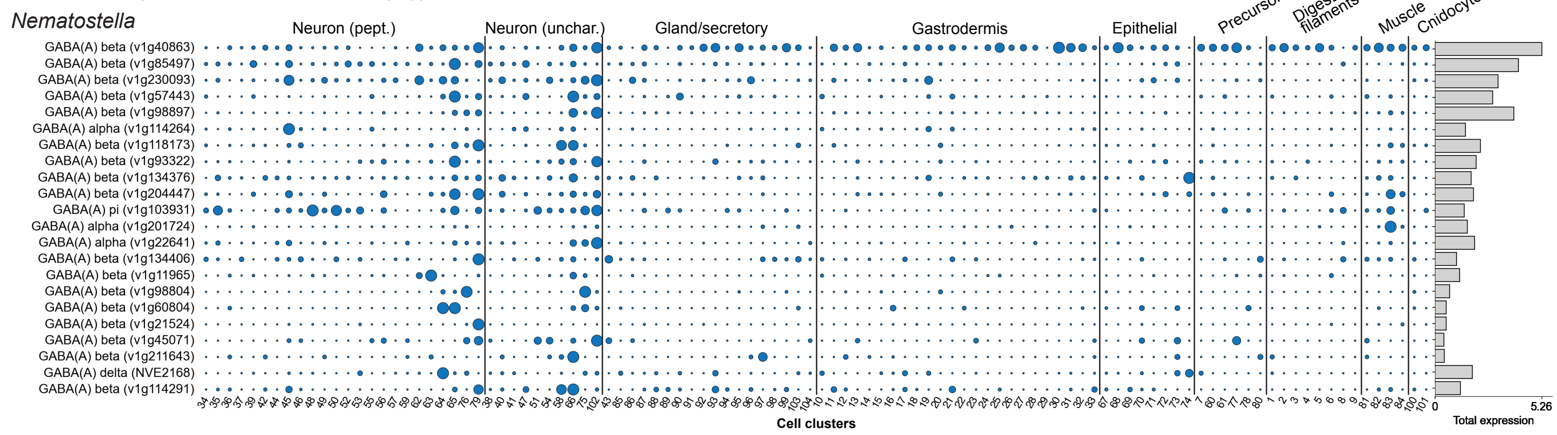

Chemical neurotransmitters

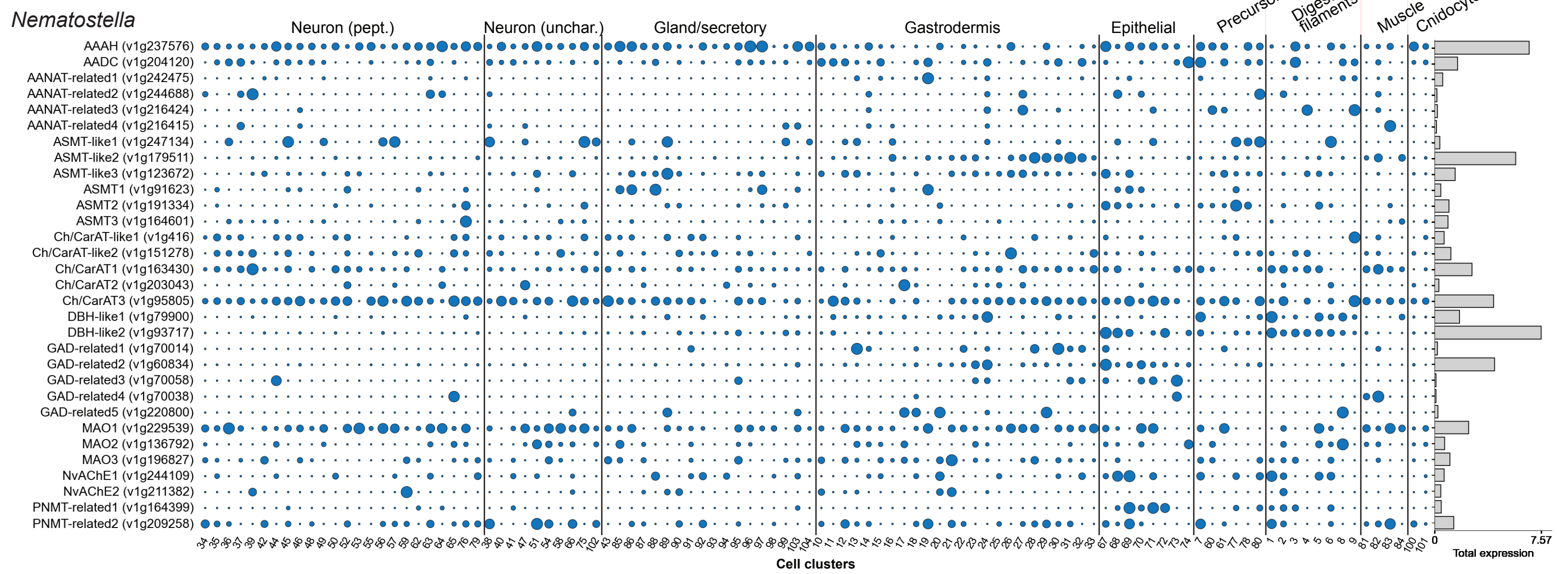

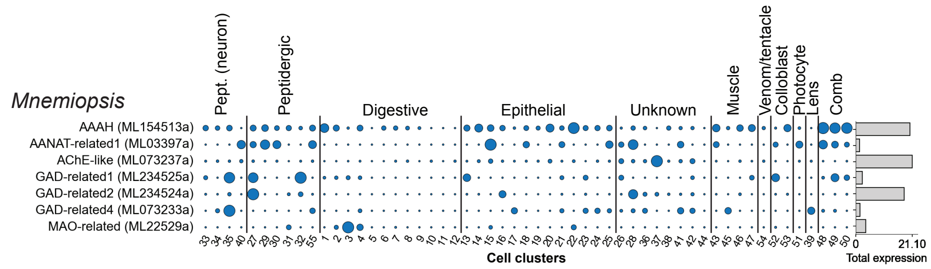

Supplement: Supplementary file 5 — Zip file containing four supplementary data files: (1) Supplementary_Data_1.docx, structures of B. mikado neuropeptide precursors and their homologues in Ctenophora; (2) Supplementary_Data_2.docx, structures of N. vectensis neuropeptide precursors and their homologues in Cnidaria; (3) Supplementary_Data_3.pdf, dotplots of gene homologues involved in neuropeptide signalling and (4) Supplementary_Data_4.fasta, AA sequences of neuropeptide precursors used for cluster analysis. [file 41559_2022_1835_MOESM5_ESM.zip › Supplementary Data 3.pdf]
